# The benefits of international volunteering in a low-resource setting: development of a core outcome set

**DOI:** 10.1186/s12960-018-0333-5

**Published:** 2018-12-20

**Authors:** Natasha Tyler, John Chatwin, Ged Byrne, Jo Hart, Lucie Byrne-Davis

**Affiliations:** 1 University of Nottingham, Nottingham, UK; 2 Health Education England, Leeds, United Kingdom; 3 University of Manchester, Manchester, United Kingdom

**Keywords:** Systematic review, Delphi, Core outcomes, International volunteering, International placements, Health professional education

## Abstract

**Background:**

Qualitative narrative analysis and case studies form the majority of the current peer-reviewed literature about the benefits of professional volunteering or international placements for healthcare professionals. These often describe generalised outcomes that are difficult to define or have multiple meanings (such as ‘communication skills’ or ‘leadership’) and are therefore difficult to measure. However, there is an interest from employers, professional groups and individual volunteers in generating metrics for monitoring personal and professional development of volunteers and comparing different volunteering experiences in terms of their impact on the volunteers. In this paper, we describe two studies in which we (a) consolidated qualitative research and individual accounts into a core outcome set and (b) tested the core outcome set in a large group of global health stakeholders.

**Method:**

We conducted a systematic review and meta-synthesis of literature to extract outcomes of international placements and variables that may affect these outcomes. We presented these outcomes to 58 stakeholders in global health, employing a Delphi method to reach consensus about which were ‘core’ and which were likely to be developed through international volunteering.

**Results:**

The systematic review of 55 papers generated 133 unique outcomes and 34 potential variables. One hundred fifty-six statements were then presented to the Delphi stakeholders, of which they agreed 116 were core to a wide variety of healthcare professional practice and likely to be developed through international experiences. The core outcomes (COs) were both negative and positive and included skills, knowledge, attitudes and outcomes for healthcare organisations.

**Conclusions:**

We summarised existing literature and stakeholder opinion into a core outcome set of 116 items that are core to healthcare professional practice and likely to be developed through international experiences. We identified, in the literature, a set of variables that could affect learning outcomes. The core outcome set will be used in a future study to develop a psychometric assessment tool.

**Electronic supplementary material:**

The online version of this article (10.1186/s12960-018-0333-5) contains supplementary material, which is available to authorized users.

## Background

Volunteering, or temporarily working in low-resource settings, is often seen solely as a means of helping those in poorer economies [1]. Many professionals find it difficult to obtain support to volunteer and report lack of recognition upon return, which is disincentive to volunteerism [2]. Furthermore, health professionals that volunteer abroad predominantly do so using annual leave, rather than recognised study leave for continued professional development [3, 4]. The notion that those from high-income countries (HICs) are altruistically offering ‘help’ to those in low- and middle-income countries (LMICs) can also lead to a distortion of the partnership relationship between high- and low-income partners in health partnerships. The low-income partners can be seen as beneficiaries and the high-income partners seen as donors [5–7]. Furthermore, a tension often exists between UK healthcare professionals and local international staff, as the intentions or role of healthcare professionals and students is often not explicit to the teams with whom they are working. However, the donor-recipient relationship is becoming increasingly contested in recent literature and policy and mutual benefits realised [8, 9].

There is an imperative, therefore, to fully understand the learning outcomes that are possible for HIC health professionals working in low-resource settings and, in particular, to help recognise these activities as educational development [2, 3]. Understanding ‘what’ is gained would allow specification of intended learning outcomes for training and continuing professional development and to make the gain for the HIC more explicit. Understanding under what circumstances learning outcomes occur would result in an understanding of how to maximise that gain.

Literature that explores what and how healthcare professionals learn from temporarily working or volunteering in a low-resource setting tends to report anecdotes or single reports, which provide a lower level of evidence [4, 10]. Furthermore, benefits are detailed in broad categories, with ‘leadership’, ‘communication’ and ‘cultural awareness’ being frequently reported [3, 11–13], with a focus on one of these skill sets in depth or a list of outcomes under umbrella terms, such as communication or leadership skills [3, 14]. These broad labels make an assessment of the learning outcomes difficult as they might contain multiple underpinning knowledge, skills, practice and attitudes. Self-assessment of broad terms is not well associated with objective performance [15]; individuals struggle to assess themselves in relation to ambiguous or ill-defined traits [16, 17]. Specifying learning at this broad level means that the more granular levels remain unspecified. A higher-level group might contain a wide range of lower-level outcomes and might not contain others, which would reduce the content validity of an assessment.

Understanding the metrics of health professional volunteerism would have a significant impact on current continued professional development (CPD) policy because international experiences could be evidenced as beneficial to personal and professional development. Numerous policy documents about future health workforce highlight the importance of skills such as leadership, communication and adaptability [18, 19]. Such skills have been described as key outcomes of international placements in LMICs, but have yet to be quantified to enable comparison with other learning opportunities [3].

In a systematic review of the evidence of the benefits to the United Kingdom of health partnership work, Jones et al. reported 40 individual benefits grouped within seven key domains (communication and teamwork, clinical skills, management skills, patient experience and dignity, policy, academic skills and personal satisfaction and interest). There were a number of features of this review that makes it insufficient for the purposes of measuring learning outcomes from international volunteering. Firstly, this review focused only on health partnerships, a specific type of health link, and not all types of volunteering or international placements. Secondly, the findings were categorised broadly, with the difficulties of broad measurement specified above. Thirdly, the professions in their search terms were only doctors and nurses. Finally, it did not extract factors that may affect learning outcomes. For the purpose of measurement, we needed to include literature from a broad range of experiences, extract outcomes at a granular level, include all healthcare staff groups and extract variables that may affect these outcomes.

The outcomes for health professionals are not always positive and the costs of international placements in literature have included health consequences, skills degradation and financial cost [3, 20, 21], reputational, health and opportunity [3]. Research has explored the costs and benefits of international placements [13, 20, 22] and barriers to volunteering, but no research has yet listed all reported negative outcome [23].

Many aspects of LMIC placements are different from working in a HIC. Relationships between outcomes and these aspects have been proposed, for example that individuals learnt from the opportunity to interact with more patients or conditions than in the United Kingdom [10, 24] and that longer stays may be more beneficial than shorter stays [25, 26]. These variables have not been systematically reported.

This current paper presents two studies: a meta-synthesis and a Delphi. The meta-synthesis aimed to (a) detail the personal and professional development outcomes of international work, at a granular level, i.e. ‘knowledge about procedures rarely conducted in the United Kingdom’ (rather than at a too broad level, i.e. clinical skills or too specific level, i.e. experience conducting vesico-vaginal fistula surgery); (b) to report the variables that influence these personal and professional development outcomes; and (c) to explore if the review including all types of UK health professional placement and all cadres of staff found the same categories as the most recent review of Jones et al. [3]. The Delphi study aimed to gather consensus from those with knowledge and expertise in international health professional learning and development, to refine a set of agreed core outcomes.

## Method

### Study 1: Meta-synthesis study design and sample

The systematic review of peer-reviewed literature, published in academic journals, was conducted between September and November 2014. Inclusion criteria included that (1) participants must not be in receipt of their full UK salary (a stipend or living allowance was permissible), thus excluding those in permanent employment overseas; (2) health professionals or health professional students (students were included, as much research has been conducted about educational outcomes in students); (3) activities must be health-focused to ensure outcomes were related to clinical work; (4) some participants must have departed from the United Kingdom and be UK citizens (papers that included a partial UK sample were included); (5) some participants must only have travelled to a LMIC; and (6) the paper must reference something that is perceived as a benefit, cost or potential variable, (7) there were no date restrictions. Guidelines for inclusion were used to ensure consistency.

Each paper was screened by one team member (NT) to ensure that it met the inclusion criteria. A second team member (JC) independently checked the first 20% of the included papers to ensure agreement of implementation of inclusion criteria. This was then discussed in a meeting. Disagreements would have been resolved using discussion and refining inclusion criteria for greater specificity; however, the reviewers agreed on all of the papers for inclusion (Table 1).

**Table 1:** Inclusion criteria

The inclusion criteria for the systematic review were peer-reviewed literature, where: 1) Individuals are either volunteers (i.e. not in receipt of full salary) or students on international placements. 2) Activities have a health focus 3) The individuals must be from the UK travelling to a lower income or lower-middle income country 4) There is reference to (individual, institutional or national) benefits or costs or the variables that moderate/mediate outcomes 5) English Language only	

### Data sources and study selection

A standard set of terms were used to search 11 databases for peer-reviewed literature between the earliest date indexed and the time of the review. This included five columns of synonyms relating to outcomes and variables, international volunteering placements, health professionals, the United Kingdom and LMICs (see Additional file 1). The databases were medical and generic databases: Cochrane Economic Evaluations, Health Management Information Consortium, Health Business Elite, Web of Knowledge/Social Sciences Citation Index, PsycINFO, CINAHL, AMED, International Bibliography of Social Sciences, Social Services Abstracts and Sociological Abstracts, Global Health and JSTOR.

The abstracts and titles of each result of the electronic database search were screened, papers that did not meet inclusion criteria were removed and retained papers were rescreened to confirm inclusion.

### Citation mapping

Reference lists of all included papers were assessed. Any papers that were of relevance were assessed against the inclusion criteria.

#### Quality assessment

We chose to include papers that were peer-reviewed but did not present empirical findings; therefore, the Cochrane risk of bias tool was not applicable to this research [27]. We categorised the papers using a quality framework [28].

### Data extraction

We took a thematic synthesis approach to data extraction [29], which consists of three stages: line-by-line coding of text, development of descriptive themes and generation of analytical themes. We did not undertake the third stage as our purpose was the extract outcomes as a low level and the third stage has been criticised for being open to the judgement of the researcher [29, 30].

Each study that met the inclusion criteria was read, and any text (related to variables or positive/negative outcomes, at an individual, national or institutional level) was coded according to both content (explicitly stated in the papers) and meaning (inferred by the researcher). Outcomes were defined as anything that happens to UK health professionals as a result of volunteering/international placements (at an individual, national or institutional level), both positive and negative. Variables were any factors that reported influence outcomes, both implicitly and explicitly.

Using Nvivo, a node was created at a ranked level for each component of descriptive theme. For example, the outcome experience conducting ‘vesico-vaginal fistula surgery’ was coded within the second-order theme of ‘greater knowledge of procedures not used in the United Kingdom’ within the higher-order theme of ‘Increased awareness of and knowledge about conditions and procedures rarely encountered in the United Kingdom’. We decided that the lowest level of specificity would be applicable to all/most professions and generalisable across situations. As each paper was coded, the nodes were adapted, developed and generated. Two researchers (NT, JC) independently reviewed the first 20% of papers and then met to develop a coding framework together. There were no disagreements as we were not looking to categorise, but rather develop a matrix of emerging codes; therefore, any differences in extraction occurred only when one reviewer had overlooked an outcome cost or variable. The second reviewer verified the extraction of the data from a further 20% of papers.

### Study 2: Stakeholder Delphi

#### Design

We used the Delphi method, an iterative process of rounds in which data are collected and condensed into a group consensus [31]. A series of virtual questionnaires record participant’s agreement with statements concerning a particular topic. Delphi is often used to develop COS in health research [32, 33]. As we were creating a core outcome set, this stage of the process only included the outcomes extracted in study 1; variables were not included.

In round 1, we held a face-to-face discussion group with stakeholders to generate outcomes. Subsequent rounds were online (with paper version emailed if there were technical difficulties). Participants were asked to indicate to what extent they agreed or disagreed each outcome was a *core outcome* of *international placements and volunteering*.

#### Participants

Participants were people who were volunteering health professionals; coordinators of international health professional volunteers, responsible for intended learning outcomes (ILOs) for health professionals; coordinators of health partnerships; study health professional education and international development; educational commissioners and NHS stakeholders. Participants were recruited for an initial workshop from a global health network, to ensure that participants from each of the stakeholder groups were invited and represented. Non-attendees were invited to participate online. After this event, a snow-ball sampling technique was used to reach further stakeholders from each group for online rounds; participants were asked to recommend interested individuals.

#### Instrumentation round 1: Stakeholder face-to-face discussion and pilot

In order to generate a list of outcomes, any new data generated from round 1 was added to the existing coding framework (see Additional file 1). Outcomes were then generated by presenting the highest-order theme as the outcome and any relevant lower-order themes as examples within brackets to add context. We input outcomes from the meta-synthesis and any additional outcomes from round 1 of the Delphi, into the hosting software. We piloted round 2 with seven members of the research team, who commented on structure, grammar, wording, level of specificity and technical issues. With the addition of items from the Delphi round 1 and comments from the pilot (and separation of some outcomes into two unique outcomes), the 133 outcomes from the meta-synthesis were converted into a list comprising of 156 outcomes to go forward to round 2.

#### Rounds 2–4: The online rounds of the Delphi

Two team members divided the 156 outcomes into three categories (see Table 2): knowledge, skills and attitudes (*n* = 115); organisational outcomes (*n* = 8); and negative outcomes (*n* = 33). Statements were presented alongside a 7-point Likert-type scale, regarding agreement as to whether each statement should be “considered a ‘core outcome’ of international placements that should be measured in a toolkit”. The scale used the following numbers to represent agreement: 1 = strongly disagree, 2 = disagree, 3 = slightly disagree, 4 = no preference, 5 = slightly agree and 6 = agree, 7 = strongly agree. For emphasis, the phrase ‘core outcome’ was presented in bold and the definition was repeated in numerous emails, instructions and synopsis. A core outcome was defined in the following way:

A core outcome is something that is common, important and applicable across a wide range of settings. It can be a benefit or cost, but it must be something that would be more likely to happen to an individual on international placement rather than somebody working in the UK.

**Table 2:** The three questions presented to stakeholders

1) KNOWLEDGE, SKILLS AND ATTITUDES: to what extent do you believe the following is a CORE outcome of international placements (that should be measured in a toolkit)? 2) ORGANISATIONAL OUTCOMES: to what extent do you believe the following is a CORE outcome of international placements (that should be measured in a toolkit)? 3) NEGATIVE OUTCOMES: To what extent do you believe the following is a CORE outcome of international placements (that should be measured in a toolkit)?	

For each round, participants had 14 days to respond. Email reminders were sent to invitees frequently. However, as the initial questionnaire was particularly long, some participants requested an extension of the deadline by 10 days and 2 days at round 3. In round 4, participants who had not responded in round 3 (but had in round 2) were invited to re-join the study; many stakeholders worked internationally and had limited internet access at certain periods. In round 4, the expressions of some statements were changed in light of the comments from previous rounds to improve clarity.

The statements with at least 70% consensus in the previous round were retained and not re-presented to the group. Therefore, by round 4, a much smaller group of non-consensus statements were presented. In rounds 3 and 4, participants were asked to use the same Likert scale and reconsider their answers from the previous round (displayed) in light of the group median and any anonymised comment gathered in the previous round.

## Results

### Study 1: Meta-synthesis

#### Data sources

The search of the electronic databases generated 521 hits including duplicates, i.e. 384 unique papers. Twenty-two papers met inclusion criteria. Citation mapping revealed a further 33 papers which were included. Therefore, the total number of papers from which data was extracted was 55. The main reasons for exclusions of papers were (1) not concerning the subject of interest, (2) non-British populations, (3) no health focus, (4) only placements in HIC, 5) only including paid/permanent staff and (6) reporting no benefits, outcomes or costs.

No papers included fell within the top two quality categories proposed by Benzies et al.: randomised controlled trials [28]. Some papers included qualitative or quantitative data (23/55, 42%), but the majority of papers reported no primary data.

**Fig. 1:**
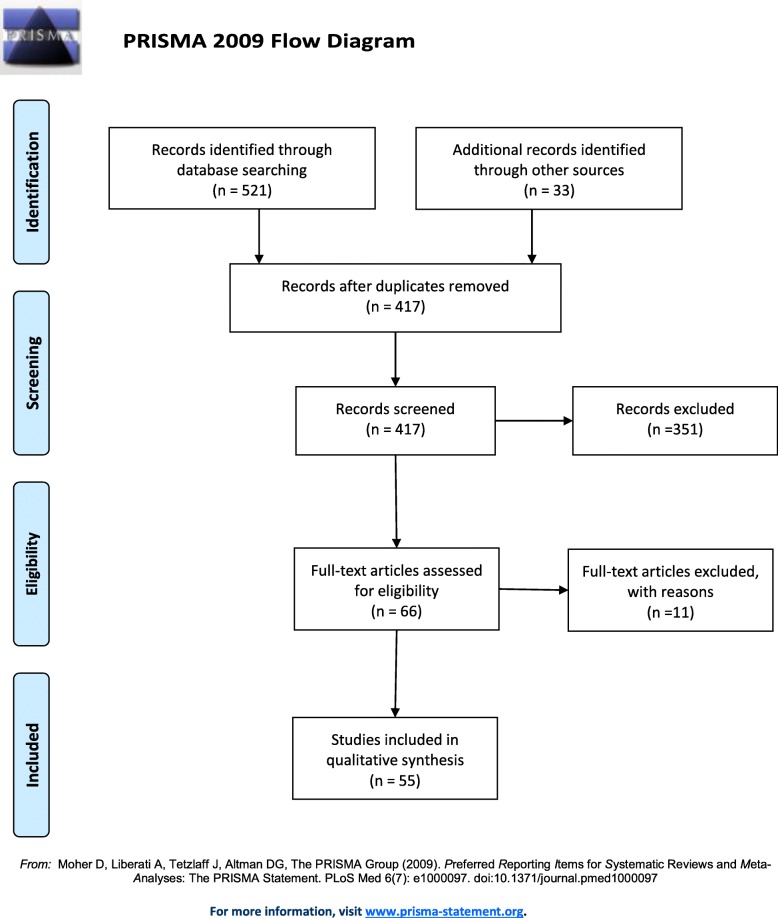
PRISMA flow chart to show number of papers included and excluded

Positive outcomes were extracted from 96% (53/55) of the papers, whilst negative outcomes were extracted from only 49% (27/55). Potential variables that could affect these outcomes were extracted from 90.91% (50/55) of papers. None of the papers explicitly reported or explored how variables were thought to affect outcomes (Fig. 1 and Table 3).

**Table 3:** Factors which influence outcomes

Higher order themes	Lower order Components	Examples from data
External Variables		
Ethics	Are local patients informed of the risk? Corporate and social responsibility Do patients come first? Levels of standards Health and Safety	“For example, it was not uncommon at first for an anaesthesiologist to encounter a complex paediatric patient having major surgery in the operating theatre where she was expected to proceed with anaesthesia without question and without preparation of adequate drugs or equipment.” (Kinnear, 2013) “I just think the really important thing in the drawbacks is the health and safety issues-I think we have that as the biggest drawback-on both sides really; the volunteers and the patients in host countries” (Workshop Participant)
Funding	Consistency of funding for project Finance plan for project Funding from a charity or grant Volunteer funded by sending organisation Volunteer fundraising Support of a health link partnership Self-funding Specific funding for training	“The period of external funding is drawing to a close and the link needs more regular and predictable funding to ensure sustainability.” (Baillie, 2009) “All international experiences are financed by the students either by assistance from grant awarding bodies, fund raising activities or personal finance.” (Thompson, 2000)
Decision of host countries needs	Needs Assessment by both parties High income party decides Host country decides	“In South Africa, for example, the government tries to fill all clinical posts with local doctors. Only when a post has not been filled by a local doctor does the government seek external applications for which UK GP trainees can apply.” (Kiernan, 2014)
Healthcare facility factors	Does the environment favour flexibility Does management allow people to become multi-skilled Level of organisational support Use of specific activities/sessions for learning Volunteer exposure to numerous systems Opportunities for exposure to culture outside of hospital Differences in protocols Licensing and professional regulations Level of corruption Are volunteer skills best utilised? Encouragement and motivation of volunteers Financial and human resources Criticism of project/volunteers Mobility of local staff Existence of local role models Number of times volunteers and local professionals engage	“This support is, by necessity, mostly provided by the host supervisor, and home medical schools in effect delegate their duty of care to the host.” (Lumb, 2014) “Students should be exposed to a variety of nursing experiences within the host country. This would give them a broad spectrum for comparisons between cultures, nursing practice and health care delivery in those cultures” (Button, 2005)
Benefits for host organisation	Donations Material/financial benefits Payment for supervision	“In order to transform a process favouring the trainee into an equitable exchange, each trainee must recognise the need for reciprocity when a community contributes to his or her education. This might manifest through the provision of resources, such as books and surgical supplies, of teaching and new ideas, or of money, which could be reallocated to meet local need.” (Banatlava, 1998)
Income of host country	Low Middle High	“They therefore concluded that there was no significant difference in level of knowledge and skill gained by going to a developed or developing country” (Button, 2005)
Commitment of local staff to project	Staff time pressures Empowerment of local staff Involvement of hospital leaders Project use local experts Local perceptions of volunteers Value of volunteer opinions	“It was reported that some overseas staff are wary of offering constructive criticism, not wishing to appear ungrateful. There is a move among many links to address this problem through structured appraisal and evaluation for each visit. One had begun to use anonymous feedback forms to learn from visits and improve the quality and effectiveness of health links.” (Baguley, 2006) “As this host explains, two prominent negative aspects are insufficient input and time” (Pearson, 2014)
Difference between host and origin country	Cultural distance between host and origin country Level of cultural immersion Severity of communication difficulties Shared values and cultural fit	“The greater the cultural differences of the international placement, the greater the impact.” (Thompson, 2000) “One of the main weaknesses has been difficulties with communication between the two partners in the link, exacerbated by problems with access to email in Uganda, intermittent exchange visits and an excessive reliance on communication through the two link coordinators. “(Longstaff, 2012)
NHS and UK Factors	Accreditation Existence of returner schemes Bureaucracy Political Climate in UK Recognition of benefits by NHS/UK organisation Trust, deaneries and PCT’s support and influence Support of UK colleagues	“This placement is recognized by the (UK) Royal College of Anaesthetists to count towards training, and these trainees will all have completed their Royal College examinations before the trip.” (Button 2005) “Many forward-thinking NHS trusts actively support relationships with overseas organisations but barriers remain.” (Dean, 2013)
Relationship between host and sending organisation	Dependence on one-another Quality of communication Collaboration Differing expectations Equality of input Ground rules and protocol How the link is set up Multi-departmental partnerships Registered links i.e. THET Sensitivity to local contexts Sustainability of relationship Length of relationship Uni-professional or multi-disciplinary	“Links are not properly established until a visit has given collaborators time to become familiar with each other and to plan the first year, at least, of their work together.” (Parry, 1998) “Links forged as trainees on these initial UROLINK visits have often been strengthened, and centres where these trainees have become consultants are now ‘twinning’ to continue the two-way exchange of experience.” (Gujral, 2002)
Level of supervision and support	Mentor in UK Support in UK Supervision from western staff residing in host country Linking of senior and junior volunteers Supervision from local people Support structure in host country Access to HR	“less support from organisational structure, developed skills as a result’ (workshop participant) ‘the supervision styles of host supervisors as the major challenges faced ‘(Horton, 2009)
Existence of other similar project in areas	Over-crowding of volunteers in hospitals Support from others volunteers in another project	“specialises in delivering high-quality primary health care in very hard to reach communities, where government service provision is non-existent and where there are very few other NGO projects” (Nunns 2011)
Focus of project	Agreement of focus Focus on mutual benefit Alignment of project with host country health plans Capacity building focus Service delivery focus Developmental focus Sustainability focus Training focus	‘For IMV placements to work, both host and volunteer need to have realistic goals and a common understanding of the aims of the placement.” (Elnaway, 2013) ‘The most commonly-reported roles overall were clinical service delivery in a non-emergency setting’ (Seo, 2012)
Practical Factors	Travel Accommodation Use of travel agent Documentation	some students plan their electives in groups, all travelling to a particular destination. This process often involves students planning a travel experience rather than a learning experience. (Miranda, 2005)
Structure of the programme	Aims developed by volunteers themselves Informed by other similar projects Informed by literature Coercion Continuation of project by other volunteers Involvement of local governments Countrywide initiatives Do volunteers have a project? How project is managed (i.e., well run) Existence of guidelines and frameworks Commitment/time allocation/number of UK admin staff Programme tailored to volunteer needs Spread of volunteers throughout the year Quality control of services provided by volunteers	‘undertaking project work, particularly if beneficial to the host.’ (Lumb, 2014) “It may have been helpful to obtain more input from similar programs at an earlier stage of planning, and it would be helpful in the future to establish formal links between programs or a forum for discussion” (Kinnear, 2013) ‘degree of developing country ownership’ (Smith, 2012)
Length of placement	Long term Short term Adjustment Short re-occurring trips	‘the average time out being 12 months, you really have time to get to grips with trusting people when you are volunteering that it takes that long before you can kind of be comfortable with it.’ (workshop participant)
Project evaluations	Evaluations during placement Post-placement longitudinal evaluation	‘The collection and application of feedback from hosts and volunteers, as well as the assessment of impact of such placements, are vital for ensuring that potential harms are mitigated and beneficial outcomes maximised (Elnaway, 2013)
Project retention and recruitment of volunteers	Volunteer drop out How are volunteers recruited	‘Retention of staff’ (workshop participant)
Assessment and Education	Existence of set learning outcomes and objectives Use of assessment Use of model to facilitate contextual understanding	‘it’s all about gaining global health knowledge, so that’s their basic outcome, there’s no assessment, its quite fluid’ (workshop participant)
Time of programme arrangement	In advance In country	‘Communications between Hereford and Muheza are difficult so details of each programme are arranged on arrival’ (Wood, 1994)
Training and preparation	Appropriate training and preparation before placement Contact with previous volunteers Debriefing Encouraging people to share experience Set training and preparation events Health monitoring Meeting in UK Training and preparation in country Volunteer involvement in planning	‘the intensity of the learning experience and pretrip preparation had a greater influence’ (Button, 2005) ‘subsequently question the actual benefit of their placement. Of note, this was despite the fact that all had received comprehensive pre-placement briefings and documents, and had had contact with previous volunteers’ (Elnawaway, 2013)
Type of organisation	Health Partnership Existing organisations Commercial involvement DIY/self-organised Remote or physical volunteering	‘Links forged as trainees on these initial UROLINK visits have often been strengthened, and centres where these trainees have become consultants are now ‘twinning’ to continue the two-way exchange of experience.’ (Gujral, 2002)
Transferability of skills learnt	Non-transferable skills Skills latency period Context dependency of skills	‘Areas in which responders were most easily able to transfer competencies to the UK to a moderate or significant degree were personal qualities (such as self-awareness and integrity)’ (Young, 2014)
Volunteer dynamics within project	Different disciplines of volunteers in project Number of volunteers in the project Social support from other volunteers in country Planned travel to destination as a group	‘Thus a broad range of departments become involved and a variety of activities are developed with the partner institution in the United Kingdom. As our experience grows, we are seeking to catalyse major links between medical schools and hospitals. This is preferable to a medley of individual links from a number of different institutions converging on a single overseas institution because it brings coherence to the goals of individuals and groups involved.’ (Parry,. 1998)
Volunteer Personal Variables		
Choices made/behaviour	Desire to become culturally sensitive Wanting to work outside of competency Willingness to work in dangerous situations Use of stress reduction strategies Understanding of local context Communication with friends/home Feeling like a foreigner Being realistic about achievements Engagement with project Willingness to learn language Perception of placement as negative or positive experience	‘a LMI country may present a temptation to students to undertake medical care or procedures which they would not be permitted to perform at home’ (Lumb, 2014) ‘learning the local language will enable nurses to succeed in developing relationships with patients or nursing students. In doing so, they will begin to move to the third level of cultural competence’ (Paterson, 2014)
Motivations for international placement	Professional/career motivations Personal Cultural Recognition from peers Desire to help other	‘unclear whether those who participated wanted to learn from the experience or whether they saw themselves as aiding the perceived ‘unfortunate” (Button, 2005)
Differences between volunteers	Level of advanced preparation Age Locum posts before or after Have individuals volunteered before? Stage in professional career Level of experience Use of professional leave	‘the range of professionals that are not qualified so they have to be supervised when they go out’ (workshop participant) ‘In practical terms, overseas working may be more accessible to younger GPs who have fewer family and financial commitments and may take up international work during training or during periods of job transition’ (Smith, 2014)
Mechanisms through which outcomes happen		
Opportunities for reflection	Critical reflection Set reflection tasks Debrief Self-reflection when choosing a placement Time for post-placement reflection	‘the process of critical reflection was uncomfortable for some. Critical reflection facilitated in a safe place may support individuals to transform their way of thinking’ (Briscoe, 2013)
Opportunities for clinical exposure	To experience complex situations and procedures To be thrown out of professional comfort zone To experience a different healthcare environment To experience a measure to compare UK and NHS to To experience unusual networks and hierachies To work with higher severity of illness To work with limited resources To work with many illnesses: spread and volume	‘Participation in health links provides in depth experience of these increasingly global pathologies’ (Peate, 2008) ‘cannot emphasise enough how seeing a mind-bogglingly large number of seriously ill people has helped … in [their] subsequent career.’ (Seo, 2012)
Opportunities for culturally different exposure	Risk exposure To engage with people from culturally diverse backgrounds To experience another culture To experience being a foreigner To experience challenging situations	‘being a foreigner- trigger for disturbance’ (Greatex-White, 2008) ‘the opportunity to work in complicated, poorly resourced and challenging environments’ (Kiernan, 2014)
Opportunities for skill development	To test coping mechanisms To use own approaches to care For creativity and innovation For hands on work For student/volunteer-centred approach to learning To use risk management skills To convert knowledge to know how To develop communication skills To challenge communication skills To practice clinical skills To practice speaking in another language To put theory into practice	‘There was lots of hands-on experience and opportunities to improve clinical skills (Kiernan, 2014) ‘opportunity to use skills- risk management’ (Workshop participant) ‘the opportunity to develop their clinical skills.’ (Barnabas, 1992)
Opportunities for research skill development	To research unusual areas To undertake collaborative research To conduct research mutually	‘Many doctors undertaking research in the UK become frustrated with its perceived lack of relevance to health care: research in developing countries is often more applied and the benefits more tangible’ (Banatlava, 1997)
Opportunities for leadership	To be included and opinions valued For teaching To lead and have responsibility To use risk management skills	‘opportunities to develop leadership skills’ Smith (2014)
Opportunities for atypical learning experiences	To learn about self Mutual learning	‘Nursing electives at home or abroad may be one way of encouraging nurses in the UK to consider their role and function from a different perspective” (Peate, 2008)

#### Extracted outcomes

**Fig. 2:**
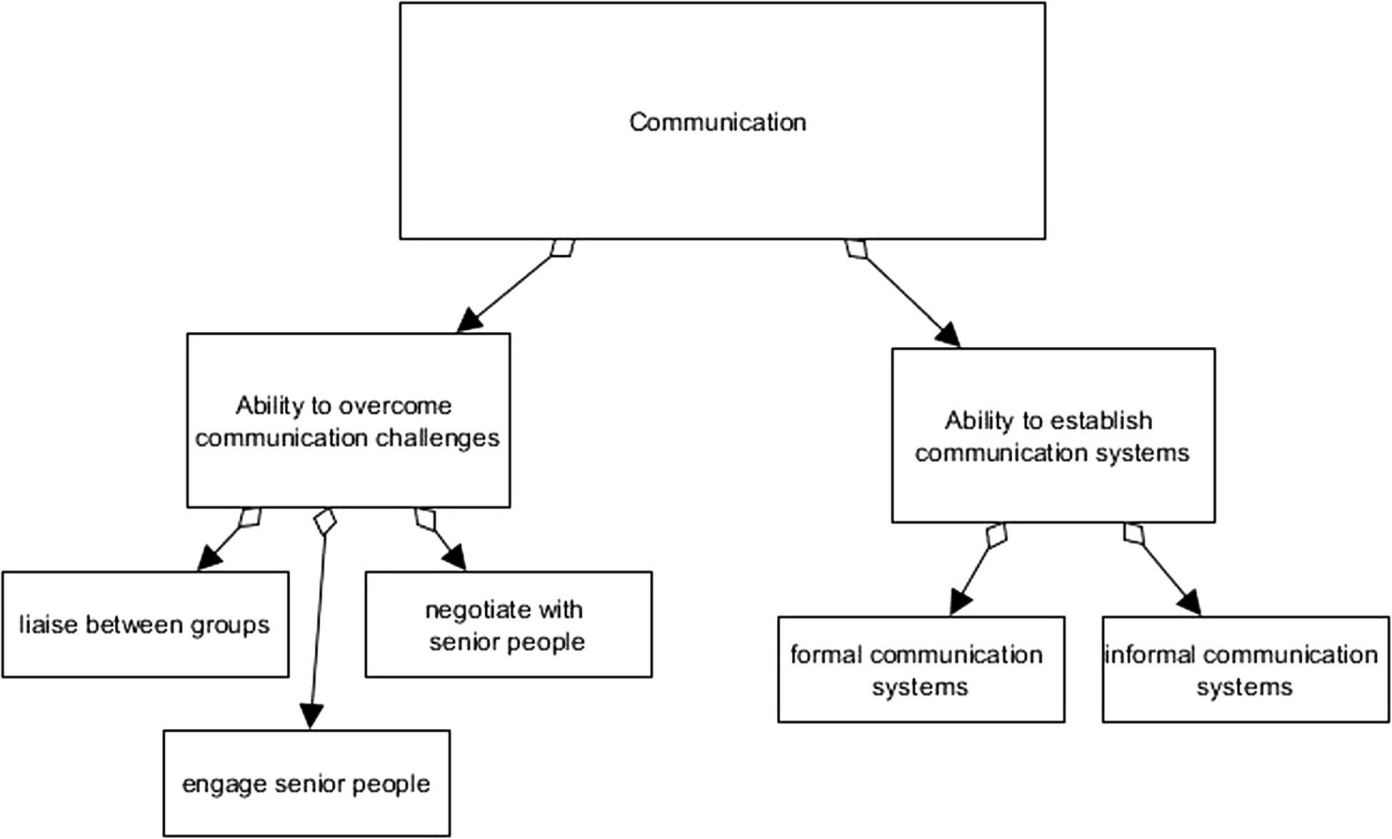
Example coding matrix (communication was not a theme, but it highlights how it was used in past research)

We found 133 unique outcomes, including 28 negative outcomes. The outcomes extracted could be categorised within NHS professional development terminology; there were 24 items about knowledge, 44 about skills and 20 about attitudes [34]. Six were organisational benefits and 29 negative; 10 were categorised as ‘other’. Organisational outcomes were deliberately separated, as organisation-specific outcomes were identified in addition to the general positive effect of staff with developed knowledge, skills and attitudes. Only 29 (22%) of the outcomes stated in the literature were negative, suggesting an overall positive attitude towards international placements from the authors (Fig. 2 and Tables 4 and 5).

**Table 4:** Percentage of papers containing positive or negative outcomes

Positive outcomes 96% Negative outcomes 49% Variables 91%

**Table 5:** How the data extracted was coded, including higher-level outcomes, lower-level outcomes and examples from the data

Outcome: highest-order theme	Second-order theme	Example data from source
Knowledge		
Increased awareness of and knowledge about how communication between two people can affect understanding	Effectively conveying ideas in an contextually appropriate way	‘Effectively conveying and receiving ideas and messages in appropriate ways so that information is carried in context’ (workshop participant)
Increased awareness of and knowledge about conditions and procedures rarely encountered in the United Kingdom	Greater knowledge of procedures not used in the United Kingdom Better management of conditions that are not common in the United Kingdom	‘Experience of unfamiliar pathologies’ [14] “Experience has been gained in open operations now rarely performed in the UK, including vesico-vaginal fistula surgery” (Gujral 2002)
Increased awareness of and knowledge about the importance of assessing healthcare on an individual basis	The uniqueness of each patient	“Enhanced the students’ cultural awareness and made them more aware of the need to assess healthcare needs on an individual basis” [25]
Increased awareness of and knowledge about the importance of community participation in health	The importance of community involvement in health Awareness of the role of the community in improving healthcare Understanding the importance of community work	“The investigators reported a significant growth in participants’ awareness of how nurses interacted with the village as a community” [36]
Increased understanding of basic skills and ideas	Core skills often replaced by technology (basic observations, using eyes, relying less on lab tests)	‘It kind of makes you go back and think about things in their fundamental…of course physics and that kind of thing’ (workshop participant)
Increased awareness of and knowledge about clinical knowledge in relation to other professions	Doctors about nurses and vice versa	‘Facilitate exploration of a different health care profession’. [36] ‘Improved interdisciplinary teamwork’ (Lee et al. 2011)
Increased awareness of and knowledge about the importance of mutual learning and respect		‘Acknowledgement from the participants that the learning was a two way process’ (Standage et al. 2014) ‘Mutual respect’ (workshop participant)
Understanding how to be a good teacher	Understanding how to target training most effectively Ability to suggest and acknowledge improvements in teaching Understanding importance of experiential learning	‘Makes you drill down more and more what makes a good teaching programme’ (workshop participant) ‘Learning in this context has enabled me to suggest ways to improve the facilitation of learning’. (Lovatt et al. 2011)
Increased awareness of and knowledge about the importance of relationship maintenance skills	Consciously making an effort to get on with colleagues Learning colleagues names	‘Increased appreciation of and skills in maintaining of relationships’ [3]
Increased awareness of and knowledge about the positive impact of clinical policies and governance	Greater policy skills	‘Work overseas will enable the health care worker to develop a greater understanding of socioeconomic and political determinants of health and consider the benefits of alternative health systems and health care initiatives’. (Banatlava, 1997)
Increased awareness of and knowledge about tropical diseases	New knowledge of tropical diseases and increasing existing knowledge	‘Knowledge of tropical diseases has increased’ (Wood et al. 1994)
Increased awareness of and knowledge about appropriate clinical behaviour	Knowing when to ask for help Knowledge of different populations needs	‘Specifically for people from other cultures’. Remembering to let people speak to husband or want to pray. Not talking to baby when it comes out. ‘(workshop participant)
Increased awareness of and knowledge about the cultural aspects of health	Greater understanding and appreciation of health promotion Understanding how culture affects daily occupation Increased understanding of cultural differences in health Understanding the effects of politics on health Understanding how culture affects you professionally Understanding how to incorporate health beliefs into a shared decision Greater understanding of sustainable healthcare	‘The noticeable lack of parental input in caring for their hospitalised children compared with UK culture and practice’. (Standage et al. 2014) ‘Increased understanding of the importance of culture in health care and the degree of variability in the countries they visited’ [25]
Increased awareness of and knowledge about global issues	Re-evaluation of world issues Deeper engagement with issues of equality and diversity Greater global knowledge	“Both learners and institutions potentially will gain from an enhanced awareness of global health issues”. (Lumb, 2014)
Increased awareness of and knowledge about cultural differences and similarities	Understanding key issues within a culture Understanding culturally acceptable behaviour Learning about other cultures Being more attentive to subtle clues about cultural differences Accepting cultural differences Understanding of cultures of UK immigrants Changed assumptions of culture	‘In Mexico it was inappropriate for them to discuss family planning methods with females because it was common for the males to exert control over such matters’. (Standage et al. 2014) ‘They could apply this new understanding to immigrant communities in the UK who had come from these cultural backgrounds’. (Standage et al. 2014)
Increased awareness of and knowledge about ethical considerations	Through experiential learning	‘This process of challenging assumptions appeared to help student to appreciate the child rights stance promoted in the UK’. (Standage et al. 2014)
Increased awareness of and knowledge about the need for/importance of training	Understanding how important effective training is in the United Kingdom and overseas	‘I recognised the need [for] teaching, so trained as a GP trainer’. (Smith et al. 2002)
Increased awareness of and knowledge about how other healthcare systems function	Developed insight into disparities within healthcare systems Increased understanding and awareness of other systems	‘Gain a more effective measure by which to evaluate the strengths and weakness of their own country’s health care system, and further develop insights into disparities’ [36]
Increased self-awareness	Awareness of own skills and limitations Able to challenge own beliefs Able to reflect on own situation Able to self-define	‘Also made me more aware of my own values and beliefs and broadened my mind’ (Greatex-White, 2008)
Increased awareness of and knowledge about finance in healthcare	Awareness of the costs of healthcare	‘There is an acute awareness of the costs of healthcare delivery especially when confronted by patients who have to pay for each intervention’ (Longstaff, 2012)
Increased awareness of and knowledge about the resistance of culture	Understanding how to make small changes Being innovative in overcoming language and cultural difference Understanding not to enforce your perspective onto others	‘To demonstrate cultural competence, nurses should reflect on and recognise their own biases and be open to other perspectives, rather than trying to persuade others to see things their way’. (Paterson, 2014)
Increased awareness of and knowledge about culture in practical assessments	Understanding importance of collecting relevant cultural information about people’s presenting health problems Learning how to conduct cultural assessments and culturally based physical assessments	‘Better understanding of cultural differences and of the need to acknowledge them in the delivery of health care’. (Paterson et al. 2014)
Increased awareness of and knowledge about the importance of trust within healthcare systems and staff	Understanding other people’s perceptions of trust	“Understanding of perceptions of trust, risk taking behaviour and approaches to risk management style”. [6]
Increased awareness of and knowledge about how systems work	Able to identify stakeholders and change agents Awareness of value systems Understanding influencing patterns of those in power Ability to assess impact of healthcare systems Understanding the difficulty of questioning an organisation	‘Had come to understand a lot about how host countries health systems operate. They were also able to make direct comparisons with the British health care system’ (Standage et al. 2014)
Skills		
Ability to overcome communication challenges	Liase between groups Engage senior people Negotiate with senior people	‘Ability to have challenging conversations about sustainable change’ (workshop participant)
Ability to communicate non-verbally	Developed non-verbal techniques	‘Developed nonverbal techniques’ [36]
Ability to provide better care	Ability to provide multicultural care Ability to develop most effective approaches to care Taking responsibility for providing quality care	‘Taking responsibility for developing quality of care’ (Banatlava, 1997)
Ability to observe and examine patients	Increased intuitive knowledge of clinical signs Ability to make diagnosis without investigations Increased clinical judgement	“In particular, UK doctors ‘honed’ their clinical diagnoses when laboratory confirmation was not available”. [24]
Ability to be innovative with clinical skills	Use of innovative techniques New ways of working)	‘Innovation in healthcare delivery and use of resources’ [3]
Ability to use a broader range of clinical skills	Enhancing existing skills and acquiring new clinical skill	‘Clinical skills were better and that the trainee had a broader range of skills’ [35]
Ability to apply clinical skills to another context	A more challenging environment or a low resource setting	‘They gained hands-on experience of care and developed a keen awareness of how the principles of nursing were applied in contexts very different from that to which they were used’. [25]
Ability to work with limited resources	Being more resourceful Ability to target resource Ability to find solutions despite limited resources Ability to work without reliance on technology Ability to manage in a low resource setting Understanding the reasons behind lack of resources	‘The nurses and doctors there are resourceful with what they have to use. I have learnt a lot and it has made me think differently. [4]
Ability to ‘get the best out of people’	Encouraging people to work together Empowering people to recognise their own strengths and to take possession of their own work/projects Ability to assess the capability of others Encouraging people to work together	‘Empowering them to recognise their strengths and not deskilling them’ (workshop participant)
Ability to manage risk	Manage risk in advance Evaluation of environment Understanding the clinical importance of risk management Understanding the wider implication of poorly managed risk	‘To manage risks they would not normally be exposed to’ (Morgan, 2012)
Ability to negotiate with multiple stakeholders		‘Improved skills of negotiation with multiple stakeholders’ [3]
Ability to make independent clinical decisions	Ability to make an urgent decision in an emergency Dealing with uncertain outcomes	‘More independent clinical decision making, eg in an emergency situation’ (workshop participant)
Ability to manage time and prioritise	Ability to respond quickly in an emergency Prioritisation of limited resources	‘Time management and prioritisation’ (workshop participant)
Ability to work within a system with unfamiliar power systems		‘Power relationships very difficult to manage’ ‘understanding the power context’ (workshop participant)
Ability to fulfil future leadership roles		‘Prepare them for future leadership roles within their profession’ [36]
Ability to plan and organise	Able to set direction	‘Planning and organisation’ (Pearson et al. 2014)
Ability to improve service	Including renewed enthusiasm for service improvement	‘Service improvement’ [11]
Ability to transfer skills and knowledge to another context		‘Applying those skills in a different context’ (workshop participant)
Ability to work towards solutions	Solution focused approach	‘Solutions despite resource constraints’ [36]
Ability to find facts to solve problems		‘They all recognised improvements in their ability to problem solve’ (Longstaff, 2012)
Ability to make decisions	Understanding who the decision is for Taking action on decision Make judgements	‘Better able to make decisions and take action’ [36]
Ability to co-operate		‘Enhancing their own cooperation and communication skills’ [24]
Ability to work as part of a team	Understanding team group norm Perception of roles within the group Managing personal objectives within a group	‘At a professional level, the experience enhanced team-working skills’ Longstaff, 2012)
Ability to develop friendships	Relationship formation skills Developing new friendships	‘Fostering friendships’ (Smith, 2012)
Ability to build a global network		‘They provide opportunities for personal and professional development of staff and promote the development of friendships and supportive networks between diverse communities”’ (Bagguley et al. 2006)
Ability to give and accept praise		‘Appeared to be related to the giving and accepting of praise. In this context praise was meaningful and valued and often contrasted with the inanition of the home situation’ (Greatex-White, 2008)
Ability to disseminate best practice globally		‘Fosters international networking, which leads to the dissemination of best practices’ (Horton, 2009)
Ability to be professionally competent	Wider view of profession Intellectual development Reminder of professional responsibilities Stronger work ethic	‘A wider view of their profession’ (Horton, 2009)
Developed research skills	Grant application skills Greater research skills	‘Experiential engagement with research is a desirable outcome’ (Pearson et al. 2014)
Ability to present work	Greater presentation skills	‘Ive seen them change considerable as people – by the end they are standing up and presenting their work and they really value that’. (workshop participant)
Ability to write reports and academic pieces		‘I believe this not only enhances my effectiveness as an NHS consultant, hut also the lecturing, teaching and writing that I do reflects favourably on my hospital and university’. (Banatlava, 1997)
Ability to apply knowledge gained in host system to the United Kingdom	Relating experiences back to the United Kingdom Using knowledge gained overseas to improve UK systems	‘Renewed enthusiasm for service improvement’ (Conference)
Ability to cope	Better coping strategies Ability to deal with knock backs Being unfazed by things Learning to deal with stress	‘I am more adaptable and can cope much easier with change’ (Longstaff et al. 2012)
Ability to adapt social norms to meet needs of another culture	Change behaviour to fit with social norms	‘Transcultural adaptation’ [37]
Ability to lead by example		‘Leading by example with consistency and perseverance can be successful ways to improve practice’ (Dowell et al. 2014)
Ability to exchange ideas with those from another culture	Communicate effectively with those from another country or culture	‘Interpersonal skills to live and work together with people of all nationalities and cultures’ (Paterson, 2014)
Ability to encourage others to take responsibility for own health		‘Encourage taking responsibility for health’ (workshop participant)
Ability to manage self	Own expectations Self-reliance Self-management Self-assurance	‘Self-management’ (Lumb, 2014)
Ability to manage projects		‘I gained significant experience in report writing, project planning, managing budgets and particularly human resources’. [11]
Ability to think through problems in a logical way	Analytical thinking Lateral thinking	‘The experience of clinical practice in a low resource environment stimulated lateral thinking’ (Lee et al. 2011)
Ability to establish communication systems	Formal and informal	‘Establishing communication systems, both formal and informal’. [6]
Developed teaching skills	Greater training delivery skills	‘But nurses/midwives - confidence and skills really increase, do not do teaching in the UK’ (workshop participant)
Ability to use evidence based practice	Ability to apply theory	‘Use evidence-based practice effectively and develop a broader and more sophisticated understanding of occupation’ (Dowell et al. 2009)
Ability to speak host language		‘Some people would learn new language, this could depend on how rural you are’. (workshop participant)
Attitudes		
Confidence to work in other locations	Confidence to move to another city/country Working with UK multicultural/ underserved populations	‘To live and work independently in a new community and culture’. (Morgan,2012)
Independence		‘Autonomy/independence’ [36]
Integrity		‘Integrity’ [11]
Diplomacy		‘Utilising diplomacy skills’ (workshop participant)
Humility		‘Knowing that you are sometimes wrong’ (Conference notes)
Judgement	Non-judgemental attitude Changed self-judgement	‘Yes and taking things less as face value and less judgemental’. (Workshop participant)
Proactivity	Using initiative	‘Initiative’ (Pearson et al. 2014)
Increased cultural sensitivity	Sensitivity to reasoning behind cultural differences Sensitivity towards feelings of minority Sensitivity towards language barriers	‘It involves an awareness and acceptance of cultural differences’ (Paterson, 2014)
Increased respect for other cultures		‘An understanding of and respect for other cultures’ (Horton, 2009)
Reinforced ethnic and cultural identity	Positivity about being British	“Having become a foreigner in the host country, there remained a sense of being tied to the home culture” (Greatex-White, 2008)
Patience and tolerance	Accepting and working at other peoples pace More tolerance	‘Made them more tolerant of others’ [25]
Increased confidence	In caring for clients from another culture In quality improvement methods To take bolder steps Self-confidence Confidence in professional ability In ability to address challenging situations	‘Confidence about caring for clients whose culture differed from their own’ (Briscoe, 2013)
Flexibility and adaptability	Acceptance of other ways of working Adaptation to responsibility Able to adapt more easily to unfamiliar situations Able to cope more easily with change Able to manage change Gaining a wider perspective Understanding the flexibility of roles	‘Flexibility/humility: Accepting different ways of working’ (workshop participant)
Emotional intelligence	Changed engagement with self Knowledge and world	‘Emotional intelligence’ (workshop participant)
Appreciation of importance of care and compassion	Empathy	‘Greater empathy and understanding’ [37]
Changed perception of otherness	Understanding importance of being a friendly stranger in the United Kingdom Experienced feeling like a foreigner whilst away	‘Learning cultural differences gave students the rare chance of being in a minority status, with the consequential experience of living and surviving in a foreign culture – an experience that students reported as ‘more valuable than a mere excursion’ (Morgan, 2012)
Appreciation of excellent human resource in the NHS	Multidisciplinary teams HR structures Appreciation of own profession Understanding hierarchy and the importance of each person within it Interaction between healthcare professionals	‘Through lack of team working they appreciated Resources - material and human’ (workshop participant)
Appreciation of having the right tools and equipment to be able to do the job	Resources: technical equipment, disposal equipment, cleaning products and protective equipment	‘Greater appreciation of the resources’ (Lee et al. 2014)
Appreciation of free universal health	NHS system of free healthcare for all Privilege and opportunity for UK citizens Understanding the expectations that are placed on NHS by service users	‘Able to comment and reflect on issues around the perceived inequalities of insurance based healthcare systems’ (Standage et al. 2014)
Appreciation of clinical governance procedures within NHS	Waste disposal Audit Teamwork Education system Tests and investigations Understanding that systems are not restricting	‘And a greater understanding of why we need to do the things that we do, like gaining consent from a child’ (Standage et al. 2014)
Organisational outcomes		
Increased staff knowledge and skills	Increased staff knowledge of low-cost healthcare More knowledgeable staff Staff able to discover better ways of doing things Staff more aware of waste reduction	‘Makes people more adaptable when they come back because in some areas if you have not move ward for twenty years, it is trauma just to be asked and work in ward X in the same hospital is not it? If you have got somebody that has been exposed to a range of environment, they are more likely to cover shifts’. (workshop participant)
Increased international reputation of NHS	Greater fulfilment of social responsibility)	‘Reputational development’ [3]
NHS becomes a more attractive employee (If offers staff opportunity to volunteer)		‘Link attracts potential staff’ [24]
Increased patient satisfaction	Staff better able to respond to UK multicultural populations Staff have greater relationships with multicultural patient population Staff more in tune with patients Staff more aware of individual needs of patients	‘Patient experience and dignity: understanding of patients from different areas’ [3]
Medical school more attractive to students (if allow students to go abroad)		‘Medical school benefits (programme are increasingly attractive, potentially providing a strong tool for recruitment)’ (Miranda et al. 2005)
Increased workforce productivity		‘Increased workforce productivity’ [3]
Reduction in NHS drop outs	Increased staff retention	‘Attraction & retention of (more/better quality) workforce’ [3]
Increased international reputation (of the United Kingdom)		‘96 per cent of health professionals interviewed for the study thought that the reputation of the NHS could only be enhanced by involvement in international health links’. (Longstaff, 2012)
Miscellaneous outcomes		
Upper hand when competing for careers		‘Working internationally is beneficial when competing for future employment’ (Paterson, 2014)
Increased job satisfaction	Increased motivation and morale with profession Renewed passion for work Sense of reward	‘They came back with greater job satisfaction’ (Longstaff, 2012)
Influence career pathway	Affects specialism choice Exploration of potential career pathways Pursuing careers in primary care, family practice, and public service Sub-specialism in global health, Teaching or lecturing careers Teaching responsibilities within clinical position	‘Such broadening experiences are recognized to impact upon the likelihood of working with underserved populations, and pursuing careers in primary care or public service’ (Lumb, 2013)
Refreshment and reinvigoration	Coming back to the United Kingdom refreshed and reinvigorated Bringing new ideas to the United Kingdom	‘With a rekindling of that initial desire to “change the world and help people” and refresh those values underpinning their initial vocational drive to enter the profession’. (Lumb, 2013)
Personal satisfaction	Personal achievements and challenges New experiences Experiencing a different lifestyle A holiday Personal fulfilment	‘An opportunity to travel, experience and work in a different setting, and to make a positive impact’ (Elanaway et al. 2014)
Increased motivation to learn a language		‘Enhanced your motivation and/or ability to learn a foreign language after returning to Northern Ireland?’ (Thompson 2000)
Development of a new perspective	Revising assumptions Reassessed outlook on life Seeing things differently Changed world views Changed outlook Look at everything in a new light Openness to new experiences Put things into perspective	‘They were beginning to see differently and to compare aspects of the host environment with those of home, leading to new perspectives on life’ (Greatex-White, 2008)
Escapism	Escape from agendas and workload A chance to take time out of training and practice Space to think and clarify career objectives	‘They want to escape the hassle of home’. (workshop participant)
Negative outcomes		
Costs to British patients	Bringing tropical illness to the United Kingdom	‘It is not uncommon for a few students each year to return from their elective unwell, with some of the infectious diseases occasionally brought back from electives not becoming apparent for some time, e.g. tuberculosis or malaria. This has significant public health implications’ (Lumb, 2013)
Developing redundant or bad skills/attitudes	Non-transferable skills Bad habits Deskilling Overconfidence in ability Poorer communication skills Loss of confidence	“They may be left to ‘do their best’ to manage heavy workloads with limited or no supervision, leading to the acquisition of poor practice habits”. (Barnabas, 2012)
Difficulty getting the job you want on return	Permanent jobs or training contracts	“Many of them experienced discouragement and warnings of ‘career suicide’ when proposing to opt out from accepted career pathways in Britain to work in the developing world for a short period’. (Connelly, 1995)
Loss of trained staff	Utilisation of key staff time Financial cost of losing staff Having to find cover for staff	‘Trained staff leaving their post following links’ [3]
Negative perceptions of NHS	Reputational When program run badly	‘Negative perception of the UK institution where links are run badly’ [3]
Distracted staff		‘Distracts staff from their work at the institution’ [3]
Exposure to ethical dilemmas	To work outside of competency Lack of regulation Too much responsibility	‘To encounter challenging ethical scenarios, particularly those students venturing to developing countries’ (Banatlava, 1998)
No recognition of accreditation upon return		‘Training and accreditation issues’ (Banatlava, 1998) ‘Lack of accreditation/recognition’ (workshop participant)
Reduced experience and exposure to UK procedures, protocols and research	No experience with NHS procedures that do not exist in host country Missing out on formal training and conferences No experience with chronic disease management over time No experience with health conditions that are common in the United Kingdom and not in host country Unaware of NHS protocol and updates Loss of professional networks and relationships	‘Referral experience more limited’ [35] ‘Things might be outdated’ (workshop participant)
Affects professional progression	Lengthens training Less time to prepare for exams Loss of partnerships	“The threat of having to ‘retrain’ is ludicrous when I am working in a developed country in a primary care setting essentially modeled on the British system”. [2]
Negative colleague perceptions	Colleagues have to cover	‘Negative perception of gaps in training programmes’ (workshop participant)
Use of time	Annual leave General time consumption	‘Staff generally use their ‘annual leave for the trips’. [4]
Professional revalidation issues	For consultants	‘Another common barrier was keeping up appraisal in light of the recent changes to GP revalidation’. [11]
Litigation	Legal issues involving clinical/professional risk	‘Clinical-professional risk- litigation’ (Morgan, 2012)
Security	Exposure to aggression Violence and death Becoming a victim of crime Political unrest	‘Examples range from involvement in criminal activity (either as perpetrator or victim)’ (Lumb, 2014)
Carbon footprint		‘Another health and safety issue is the carbon footprint’. (Pearson et al. 2014)
Culture shock		‘Culture shock due to the contextual differences and challenges faced in resource poor settings’. [3]
Environmental and infrastructural risk		‘Physical risk to person- environment, infrastructure’ (Morgan, 2012)
Extreme nationalism towards the United Kingdom		‘Developing negative attitudes towards host culture- causes retreat back to culture of origin and even extreme nationalism’ (Greatex-White, 2008)
Experiencing negative feelings	Feeling as though imposing on UK colleagues to provide cover Feeling out of depth Frustration Guilt and regret about death	‘I was subjected to the feelings of guilt and regret which accompany the death of a patient under one’s care’ (Robinson, 2014)
Financial loss	Costs of getting involved Loss of earnings Loss of pension or employment entitlement	‘Costs of getting involved’ [4]
Health consequences	Animal bites Tropical diseases Sexually Transmitted Disease Injuries and transport accidents Infection Jet lag Skin disease	‘11.1% were concerned that they had placed themselves at risk of HIV and STIs. Unprotected sexual intercourse was the most commonly reported reason’. [20]
Psychological consequences	Depression Anxiety Stress Nervousness	‘Psychological problems on return from their placements’ [20]
Exhaustion and burn out		‘Exhaustion/Burnout/Stress’ [3]
Loneliness	Isolation Social isolation No or few friends in host country	‘You will often be doing lone working which will be very high risk and that happens an awful lot’. (workshop participant)
Missing things at home	Missing life in the United Kingdom Time away from family and friends	‘Time away from their family’ [36]
Loss of interest in global health and international placements	Negative perceptions of volunteering and international placements	‘Many reported negative experiences and never wanted to do it again’ (Conference speaker)
Socio-cultural risk	Exposure to corruption Experiencing resistance to western influence	‘Socio-cultural risk- dress like them, did not want English influence, corruption’ (Morgan, 2012)
Become judgemental		‘Go home with a judgmental opinion of some of the people I look after’. (workshop participant)

### Study 2: Delphi

#### Participants

Fifty-one participants attended the round 1 workshop. Invitations were sent to 259 participants for the online Delphi, and 78 (30%) accepted. Once enrolled in the study, response rates remained high: round 2, *n* = 58/78 (74%); round 3, *n* = 49 (63%); and round 4, *n* = 45 (58%). More than half of the participants were involved in global health policy, and one third of the participants had volunteered.

After round 2, 98 of the 156 statements (63%) were retained; this meant over 70% of the stakeholders agreed or strongly agreed these 98 statements were core outcomes. After re-considering their own vote in round 2, the group median and anonymous comments regarding each statement, 13 additional statements were retained in round 3. Finally, after readdressing the above items for the second time, an additional five statements met consensus and were retained in round 4. Of the items that met consensus, 99 were positive and eight were negative. Positive outcomes were of educational benefits to the British health professionals and negative outcomes were drawbacks, costs or negative effects (Tables 6, 7, 8, 9 and 10).

**Table 6:** Number of statements retained at each stage with 70% consensus being met

Round	Number of Statements retained (*n* = 156)	Positive outcomes	Negative outcomes
2	98	97	1
3	13	10	3
4	5	1	4
Did not meet consensus	40	14	26

**Table 7:** Applying our results to the current knowledge: our core learning outcomes presented within the existing domains from [3]

Domain in [3]	Number of COs within this domain	Examples
Clinical skills	12	Ability to use a broader range of clinical skills (e.g. enhancing existing skills and acquiring new clinical skills, greater all round competence) Increased awareness of/knowledge about tropical diseases Increased awareness of/knowledge about the cultural aspects of health (e.g. greater understanding of health promotion, how culture affects daily life and professional work, cultural differences in health, the effects of politics on health, sustainable healthcare)
Management skills	16	Ability to be adaptable in leading (e.g. able to lead in complex novel situations, ability to compromise not dictate) Ability to work within a system with unfamiliar power dynamics Ability to manage projects
Communication and teamwork	21	Understanding that words and behaviours can have different meanings (e.g. understanding how words are perceived by others, understanding how to speak and behave so as not offend people) Ability to co-operate (e.g. willingness to see another point of view) Ability to work as part of a team (e.g. understanding team group norms, perception of roles within the group, managing personal objectives within a group)
Patient experience and dignity	19	Understanding own potential to empower people Increased respect for other cultures Appreciation of free universal health (e.g. the NHS system of free healthcare for all, privilege and opportunity, the expectations that are placed on NHS by service users)
Service/policy development and implementation	15	Increased awareness of/knowledge about the positive impact of clinical policies and governance (e.g. understanding the benefits of a comprehensive checklist) Appreciation of excellent human resource in the NHS (e.g. multidisciplinary teams, HR structures, appreciation of own profession, understanding hierarchy and the importance of each person within it)
Academic skills	9	Ability to dissemination best practice globally Improvement in teaching skills (e.g. learning new techniques, greater training delivery skills, lecturing skills and small group teaching skills) Ability to build a global network
Personal satisfaction and interest	16	Ability to develop friendships (e.g. relationship formation skills, developing new friendships) Refreshment and reinvigoration (e.g. chance to take time away to become refreshed and feel reinvigorated to work upon return) Can-do attitude

**Table 8:** Examples of COs that fell within a number of categories

Example	Categories
Increased awareness/knowledge about clinical conditions and procedures rarely encountered in the United Kingdom	Clinical, academic
Increased awareness of/knowledge about the importance of mutual learning and respect	Patient experience and dignity, communication and team work
Ability to disseminate best practice globally	Communication and team work, academic, service improvement and policy
Ability to develop friendships	Personal, communication and team work

**Table 9:** Examples of core learning outcomes that did not fit within the categories

Core outcome	
Improved flexibility and adaptability	
Ability to be innovative when overcoming challenges	
Ability to cope	
Improved situational awareness

**Table 10:** List of all outcomes and those that met consensus (those that met consensus were included in the core outcome set)

Core outcome	Met consensus at round	Percentage consensus	Include or exclude	Rank
Increased awareness of/knowledge about cultural differences and similarities (e.g. understanding key issues within a culture, culturally acceptable behaviour and cultures of UK immigrants, learning about, accepting and changing assumptions about other cultures)	2	100	+	1
Increased awareness of/knowledge about the cultural aspects of health (e.g. greater understanding of health promotion, how culture affects daily life and professional work, cultural differences in health, the effects of politics on health, sustainable healthcare)	2	100	+	1
Ability to work with limited resources (e.g. being more resourceful, ability to target resources, ability to find solutions despite limited resources, making use of everything available, ability to work without reliance on technology, manage in a low resource setting)	2	95	+	3
Increased awareness of/knowledge about culture in practical assessments (e.g. the importance of collecting relevant cultural information about people’s presenting health problems and learning how to conduct cultural assessments and culturally based physical assessments)	2	93	+	4
Ability to apply clinical skills to another context (e.g. a more challenging environment or a low resource setting)	2	93	+	4
Ability to be adaptable and innovative in teaching (e.g. ability to transfer skills and knowledge to the most influential people or to another context, recognising different learning styles, being adaptable in assessment)	2	93	+	4
Increased awareness of/knowledge about how other healthcare systems function (e.g. developed insight into disparities within healthcare systems, understanding of other systems)	2	93	+	4
Ability to cope (e.g. improved coping strategies, ability to deal with lack of structure, knock backs and stress, being unfazed by things and taking things in stride, new approach to guilt for patients problems)	2	93	+	4
Increased cultural sensitivity (e.g. sensitivity to reasoning behind cultural differences, feelings of minority and language barriers)	2	91	+	9
Understanding that words and behaviours can have different meanings (e.g. understanding how words are perceived by others, understanding how to speak and behave so as not offend people)	2	91	+	9
Ability to apply knowledge across systems (e.g. ability to apply knowledge from host system to United Kingdom and vice versa, using knowledge gained in system to improve/change another)	2	91	+	9
Development of a new perspective (e.g. revising assumptions, seeing things differently, changed world views and outlook, look at everything in a new light, openness to new experiences, put things into perspective)	2	91	+	9
Improved flexibility and adaptability (e.g. acceptance of other ways of working, adaptation to responsibility, being able to adapt more easily to unfamiliar situations, able to cope more easily with change, gaining a wider perspective, understanding the flexibility of roles)	2	91	+	9
Ability to be innovate when overcoming challenges (i.e. finding unique ways of overcoming cultural and language challenges)	2	91	+	9
Increased respect for other cultures	2	90	+	15
Increased understanding of basic skills and ideas (i.e. back to basics, e.g. basic observations using eyes, less reliance on lab tests and technology, basic clinical skills and science)	2	90	+	15
Confidence in teaching ability (e.g. being more comfortable around others, confidence public speaking, confidence in transferring knowledge)	2	90	+	15
Improved confidence (e.g. in caring for clients from another culture, in quality improvement methods, to take bolder steps, to address challenging situations, self-confidence, confidence in professional ability,)	2	90	+	15
Confidence to work in other locations (e.g. confidence to move to another city/country, working with UK multicultural/underserved populations)	2	89	+	19
Increased awareness of/knowledge about global issues (e.g. re-evaluating world issues, shared purpose)	2	88	+	20
Increased awareness of/knowledge about conditions and procedures rarely encountered in the United Kingdom (e.g. greater understanding of procedures not used in the United Kingdom, unfamiliar equipment and delayed presentations, better management of conditions that are not common in the United Kingdom)	2	88	+	20
Increased awareness of/knowledge about tropical diseases	2	88	+	20
Increased awareness of/knowledge about the importance of mutual learning and respect (i.e. greater understanding of reciprocal learning)	2	88	+	20
Ability to be adaptable in leading (e.g. able to lead in complex novel situations, ability to compromise not dictate)	2	88	+	20
Ability to work within a system with unfamiliar power dynamics	2	88	+	20
Ability to adapt social norms to meet needs of another culture (e.g. change behaviours to fit into another culture, being aware of own social norms and adapting them)	2	88	+	20
Ability to exchange ideas with those from another culture	2	88	+	20
Increased self-awareness (e.g. understanding own skills and limitations, how to challenge own beliefs and importance of reflecting on own situation)	2	88	+	20
Patience and tolerance (e.g. accepting and working at other peoples pace, more tolerant)	2	88	+	20
Proactivity (e.g. thinking on feet, using initiative, efficiency, get on with things rather than look for someone to blame)	2	88	+	20
Ability to work with resources available in specific contexts (i.e. understanding the reasons behind lack of resources)	2	88	+	20
Ability to work towards solutions (e.g. solution focused approach)	2	88	+	20
Understanding that speed and language competency affect communication (e.g. awareness of how speed affects comprehension, understanding language differences and checking recipient comprehension, ability to use an interpreter)	2	86	+	33
Increased awareness of/knowledge about the importance of community participation in health (e.g. understanding the community and social influences on health, the role of the community in health, public health and the importance of community work)	2	86	+	33
Ability to use a broader range of clinical skills (e.g. enhancing existing skills and acquiring new clinical skills, greater all round competence)	2	86	+	33
Understanding that changing behaviour is complex (e.g. understanding how to make small changes and not to force your perspective onto others,)	2	86	+	33
Ability to improve service (e.g. renewed enthusiasm for service improvement)	2	86	+	33
Increased staff knowledge and skills (e.g. increased staff knowledge of low cost healthcare, more knowledgeable staff able to cover more areas, to discover better ways of doing things and more aware of waste reduction)	2	86	+	33
Increased awareness of/knowledge about how context affects communication (e.g. effectively conveying ideas in a contextually appropriate way)	2	84	+	39
Increased awareness of/knowledge about the need for and importance of training (i.e. understanding how important effective training is in)	2	84	+	39
Improvement in teaching skills (e.g. learning new techniques, greater training delivery skills, lecturing skills and small group teaching skills)	2	84	+	39
Ability to deal with the unexpected	2	84	+	39
Ability to manage projects	3	84	+	99
Deeper engagement with issues of equality and diversity	2	83	+	43
Ability to overcome communication challenges (e.g. ability to communicate effectively in high pressure situations, engage in challenging conversations and liaise between groups)	2	83	+	43
Ability to be innovative with clinical skills (e.g. use of innovative techniques, finding new ways to approach a condition, new ways of working)	2	83	+	43
Appreciation of having the right tools and equipment to be able to do the job (i.e. resources: technical equipment, disposal equipment, cleaning products and protective equipment)	2	83	+	43
Appreciation of excellent human resource in the NHS (e.g. multidisciplinary teams, HR structures, appreciation of own profession, understanding hierarchy and the importance of each person within it)	2	83	+	43
Improved emotional intelligence (e.g. changed engagement with self, knowledge and world)	2	83	+	43
Ability to identify and anticipate potential problems (e.g. identify problems when setting up a new project)	2	83	+	43
Increased awareness of/knowledge about appropriate clinical behaviour (e.g. knowing when to stop and when to move forward, when to ask for help and different populations needs)	2	82	+	50
Ability to make independent clinical decisions (e.g. ability to make an urgent decision in an emergency, dealing with uncertain outcomes, evaluating risks to patients and self)	2	81	+	51
Understanding own potential to empower people	2	81	+	51
Ability to work as part of a team (e.g. understanding team group norms, perception of roles within the group, managing personal objectives within a group)	2	81	+	51
Ability to build a global network	2	81	+	51
Ability to disseminate best practice globally	2	81	+	51
Appreciation of free universal health (e.g. the NHS system of free healthcare for all, privilege and opportunity, the expectations that are placed on NHS by service users)	2	81	+	51
Improved situational awareness (i.e. understanding your environment so you can understand what to do)	2	81	+	51
Increased job satisfaction (e.g. increased motivation and morale within profession, renewed passion for work, sense of reward)	2	81	+	51
Personal satisfaction (e.g. personal achievements and challenges, new experiences, experiencing a different lifestyle, a holiday, appreciation of own life, personal fulfilment)	2	81	+	51
Can-do attitude	3	81	+	100
Ability to co-operate (e.g. willingness to see another point of view)	2	79	+	60
Appreciation of clinical governance procedures within NHS (e.g. waste disposal, audit, teamwork, education system, tests and investigations)	2	79	+	60
Appreciation of the importance of care and compassion (e.g. ability to compare compassion in both systems, empathy and fairness)	2	79	+	60
Ability to provide better care (e.g. ability to integrate primary and secondary care, to provide multicultural care, to develop most effective approaches to care and taking responsibility for providing quality of care)	2	79	+	60
Increased awareness of/knowledge about the positive impact of clinical policies and governance (e.g. understanding the benefits of a comprehensive checklist)	3	78	+	101
Increased awareness of/knowledge about ethics (i.e. experiencing ethical dilemmas, understanding the importance of ethics)	2	78	+	64
Changed perception of otherness (e.g. understanding importance of being a friendly stranger in the United Kingdom, feeling like a foreigner)	2	78	+	64
Integrity	2	78	+	64
Independence (e.g. lone working)	2	78	+	64
Ability to plan and organise (e.g. ability to set direction, improved audit skills)	2	78	+	64
Ability to make decisions (e.g. understanding who the decision is for, taking action on decision, making judgements	2	78	+	64
Ability to manage risk (e.g. manage risk in advance, evaluation of environment, understanding the clinical importance of risk management and the wider implication of poorly managed risk)	2	78	+	64
Increased patient satisfaction (e.g. staff better able to respond to UK multicultural populations, staff able to compare how systems affect patient satisfaction, have greater relationships with multicultural population, more in tune with patients and more aware of individual needs of patients).	2	77	+	71
Ability to communicate non-verbally	2	76	+	72
Ability to establish communication systems (e.g. formal and informal)	3	76	+	102
Increased clinical knowledge in relation to other professions (e.g. doctors understanding nurses and vice versa, multi-disciplinary awareness)	3	76	+	102
Ability to get the most out of people (e.g. encouraging people to work together, recognise their own strengths and to take possession of their own work/projects, ability to assess the capability of others)	2	76	+	72
Ability to manage people (e.g. able to allocate tasks and co-ordinate people, to deal with people with differing objectives, to negotiate with multiple stakeholders, to manage difficult people)	2	76	+	72
Ability to develop friendships (e.g. relationship formation skills, developing new friendships)	2	76	+	72
Ability to manage self (e.g. own expectations, self-reliance, self-management, self-assurance, reflexivity)	2	76	+	72
Changed judgement (e.g. non-judgemental attitude, changed self-judgement)	2	76	+	72
Diplomacy	2	76	+	72
Ability to find facts to solve problems	2	76	+	72
Developing redundant or bad skills/attitudes (e.g. developing non-transferable skills, bad habits, deskilling, returning with overconfidence in own ability, poorer communication skills, loss of confidence)	3	76	–	102
Financial loss (e.g. costs of getting involved, loss of earnings, pension or employment entitlement)	4	76	+	112
Reduction in NHS drop outs (e.g. increased staff retention, when they volunteer and come back to NHS)	3	75	+	105
Ability to observe and examine patients (e.g. increased intuitive knowledge of clinical signs and clinical judgement ability to make diagnosis without investigations)	2	74	+	80
Ability to work in a professionally competent way (e.g. having wider view of profession, intellectual development, reminder of professional responsibilities, stronger work ethic)	2	74	+	80
Increased understanding of how to be a good teacher (e.g. allowing students to learn from mistakes, ability to suggest and acknowledge improvements in teaching, understanding how communication affects learning, how to target training most effectively and the importance of experiential learning)	2	74	+	80
Act as a role model (e.g. lead by example)	2	74	+	80
Influences career pathway (i.e. affects specialism choice, exploration of potential career pathways, pursuing careers in primary care, family practice, public service, sub-specialism in global health, teaching)	2	74	+	80
Ability to manage time and prioritise (e.g. ability to respond quickly in an emergency, managing immediate need vs long term need, prioritisation of limited resources)	2	74	+	80
Increased ability to change behaviour in colleagues or patients (e.g. ability to implement behaviour change and to assess the impact of healthcare systems)	4	73	+	113
Ability to manage tragedies	3	73	+	106
Reduction in staff competence (e.g. brain drain reversal: NHS loss of competent staff to overseas placements, staff unable to cope with paperwork on return)	4	73	–	113
Exposure to ethical dilemmas (e.g. expected to work outside of competency, to do clinical work, little regulation, little supervision, too much responsibility)	3	73	+	106
No recognition or accreditation upon return	4	73	+	113
Increased international reputation (of United Kingdom)	3	73	+	106
Increased international reputation of NHS (e.g. greater fulfilment of social responsibility)	2	73	+	86
Ability to verbalise knowledge (e.g. ability to verbalise core concepts and deep knowledge, ability to explain complex ideas to others)	2	72	+	87
Increased awareness of/knowledge about the importance of trust between colleagues within healthcare systems	2	72	+	87
Increased awareness of and knowledge the functioning of systems (e.g. able to identify stakeholders and change agents, understanding influencing patterns of those in power, value systems and the difficulty of questioning organisations)	2	72	+	87
Refreshment and reinvigoration (e.g. chance to take time away to become refreshed and feel reinvigorated to work upon return)	2	72	+	87
Increased awareness of/knowledge about the importance of consciously making an effort to get on with colleagues (e.g. learning colleague’s names)	3	71	+	109
Ability to manage healthcare environments (e.g. ability to manage wards and staff)	2	71	+	91
Increased awareness of/knowledge about the costs of healthcare	2	71	+	91
Ability to accept and understand failure (e.g. to continue with something that did not have desired outcome at first, learning to accept failure, thinking differently about failure, persistence)	2	71	+	91
Humility (including professional humility)	2	71	+	91
Ability to think through problems in a logical way (e.g. analytical/lateral thinking)	2	71	+	91
Ability to engage senior people	2	70	+	96
Loss of interest in profession (e.g. not wanting to work in your profession when home)	4	70	–	114
Extreme nationalism towards the United Kingdom	3	70	–	110
Health consequences (e.g. animal bites, tropical diseases, STD’s, injuries and transport accidents, infection, jet lag, skin disease)	2	70	+	96
Increased workforce productivity	3	70	+	110
NHS becomes a more attractive employer (e.g. an employer that offers staff the opportunity to volunteer)	2	70	+	96
Reinforced ethnic and cultural identity (e.g. understanding of own ethic and cultural identity)	No Con	0		
Ability to listen	No Con	0		
Increased awareness of/knowledge about the importance of assessing healthcare on an individual basis (i.e. the uniqueness of each patient)	No Con	0		
Ability to apply evidence based practice (e.g. understanding its importance (sometimes through being unable to apply it overseas), understanding how to apply it innovatively with limited resources)	No Con	0		
Ability to give and accept praise	No Con	0		
Ability to encourage others to take responsibility for own health	No Con	0		
Ability to speak the host language	No Con	0		
Ability to challenge breaches of privacy and confidentiality (e.g. ability to stand up for patients/peoples rights if they are jeopardised, increased awareness of human rights, ability to respect regulatory standards of home and overseas regulatory bodies)	No Con	0		
An upper hand when competing for careers	No Con	0		
Spiritual development	No Con	0		
Escapism (e.g. freedom from bureaucracy, space outside of regular routine to clarify objectives, escape from agendas and workload, a chance to take time out of training and practice)	No Con	0		
Improved research skills (e.g. grant application skills, research design and implementation)	No Con	0		
Ability to present work	No Con	0		
Ability to write reports and academic pieces	No Con	0		
Costs to British patients (e.g. staff desensitised, staff less tolerant and patient, staff bringing tropical illnesses to the United Kingdom)	No Con	0		
Loss of trained staff (e.g. utilisation of key staff time, financial cost of losing staff, having to find cover for staff)	No Con	0		
Negative perceptions of NHS (e.g. NHS reputation jeopardised if a health link is badly organised)	No Con	0		
Distracted staff (e.g. staff going on international placements coming back disengaged with UK work and pre-occupied)	No Con	0		
Difficulty getting the job or training position that you want upon return (e.g. returning to work in a locum position, not having a permanent job upon return)	No Con	0		
Reduced experience and exposure to UK procedures, protocols and research (e.g. NHS procedures that do not exist in host country, missing out on formal training and conferences, chronic disease management over time, health conditions that are common in the United Kingdom and not in host country, NHS protocol and updates, loss of professional networks and relationships)	No Con	0		
Affects professional progression (e.g. lengthens training, less time to prepare for exams, time for professional readjustment upon return, career suicide, loss of partnerships)	No Con	0		
Negative colleague perceptions (e.g. colleagues think it’s a holiday, colleagues have to cover)	No Con	0		
Use of time (e.g. using annual leave to spend time on international placements, physically spending time on placements that could be spent in another way)	No Con	0		
Professional revalidation issues (e.g. gaps in consultants portfolio)	No Cons	0		
Litigation (e.g. legal issues involving clinical/professional risk)	No Con	0		
Security (e.g. exposure to aggression, violence and death, becoming a victim of crime, political unrest)	No Con	0		
Carbon footprint	No Con	0		
Culture shock	No Con	0		
Environmental and infrastructural risk (e.g. being in dangerous infrastructures and environments)	No Con	0		
Experiencing negative feelings (e.g. feeling as though imposing on UK colleagues to provide cover, feeling failure, feeling out of depth, frustration, guilt and regret about death)	No Con	0		
Psychological consequences (e.g. depression, anxiety, stress, traumatisation and nervousness)	No Con	0		
Compromises of health and safety	No Con	0		
Exhaustion and burn out	No Con	0		
Loneliness (e.g. lone working, isolation, social isolation, no or few friends in host country)	No Con	0		
Missing things at home (e.g. missing home comforts, missing life in the United Kingdom, time away from family and friends)	No Con	0		
Loss of interest in global health and international placements (e.g. not wanting to do it again, negative perceptions)	No Con	0		
Socio-cultural risk (e.g. corruption, local resistance to western influence)	No Con	0		
Becoming judgemental	No Con	0		
Negative feelings towards the NHS (e.g. questioning NHS, questioning the disposable culture of NHS, having a different system to compare to NHS)	No Con	0		
Medical school more attractive to students (e.g. if allows students to go abroad)	No Con	0		

## Conclusion

This study aimed to generate a list of core learning outcomes which might be developed through international placements and variables which might affect their development. We found 55 peer-reviewed papers and extracted 133 outcomes and 34 variables Table 3. The most recent research to summarise learning outcomes [3] found 40 individual benefits in seven domains: clinical skills, management skills, communication and teamwork, patient experience and dignity, policy, academic skills and personal satisfaction and interest. Our results support the domains but present the outcomes at a more granular level. For example, the previous review reports ‘management skills’ as a domain, which includes the outcome of ‘leadership and management’. We extracted more granular knowledge, skills and attitudes which would map into the domain of ‘management’, such as ability to manage self, ability to lead by example and ability to manage risk. These more specific outcomes would lend themselves more to measurement due to the reported difficulties with assessment of domains [15, 16]. By extracting outcomes at a granular level, we were also able to highlight many outcomes that do not fit neatly into any of the pre-defined categories of previous research such as ‘ability to cope’ or those that fit into more than one, i.e. ‘ability to disseminate best practice globally’. Our study is the first to summarise the variables which have been assumed or proposed to influence learning in international placements, which will allow for hypothesis testing in the future. The outcome set provides a framework of personal and professional learning across healthcare professional groups. This is important as previous literature has tended to focus on specific professional cadres, so this COS would allow comparison and collation across professional groups [35, 36].

Our study generated a list of 28 potential negative outcomes. It is interesting that only eight of these were retained in the Delphi, i.e. stakeholders were in agreement that these negative outcomes were either not likely to happen or likely to happen to a range of healthcare professionals. Only one negative outcome was considered core: ‘health consequences’. This indicates that stakeholders believe almost all negative outcomes do not happen on many or most placements. There is much less consensus about the negative aspects of placements.

The literature contains stated or implied variables which might influence learning on international placements, and this study has synthesised these, finding 33 variables. This provides a framework for future research that aims to study the interactions between variables and outcomes by empirically testing some of the hypotheses reported or assumed in the literature.

Historically, international volunteering has been conceptualised as a benefit to the LMIC and a loss to the HIC [8, 9]. Recent policy documents explicitly discuss the benefit to UK health professionals in terms of personal and professional development and the necessity to develop competencies to be used in training curricula [9]. This study will facilitate the specification and exploration of learning outcomes and so in the future help in addressing the imbalanced discourse of the “benefitting LMIC” and the “donor HIC”. Additionally, a recent Royal College policy describes what competencies paediatricians need to work globally, or with a global population in the United Kingdom [37]. Many of the competencies described map onto the core outcome set suggesting that international placements themselves may provide a vehicle for developing these necessary competencies. In fact, the core outcome set maps onto policy documents such as the Health Education England (HEE) Framework 15: 2014–2029, which suggests the future NHS workforce needs to be flexible, open to innovation and change and life-long learners (all components of the COS) [18]. The core outcome set provides a way of framing and evidencing the NHS benefits. Future work will focus on how the core outcome set can be used as a tool to measure outcomes. The research has also influenced the production of the Health Education England Global strategy, which aims to embed global learning opportunities into NHS training [38].

In summary, there is a broad range of learning outcomes which we have synthesised into a set of 116 core outcomes agreed by a group of 45 stakeholders from various invested groups that could be used in future assessment of learning and testing of hypotheses about what leads to or detracts from learning. We also extracted 33 variables from the literature. We reported a list of negative outcomes, as well as every variable that has been reported (implicitly or explicitly) to affect learning. The core outcome set and variables will enable the development of assessments of health professional learning in international placements, which has implications for how international placements are created and on the support for international placements amongst UK healthcare organisations.

### Limitations

This study has a number of limitations. Firstly, we did not update the systematic review because this was the first stage of the outcome set development, and therefore, new outcomes could not be added. We conducted a scoping search using the same search strategy in March 2018 and found 23 new papers had been published. We read these papers and did not find any new outcomes or variables reported. Secondly, the papers included in the meta-synthesis included both those with primary data and those which did not. Formal risk of bias assessment, using standard tools, was therefore not possible. However, it is important to note that the papers included and the findings of the Delphi indicate an overall positive attitude towards international placements, with 96% of papers in the review reporting positive outcomes as opposed to 49% reporting negative outcomes. It is possible that there is publication bias, in which reports of negative experiences are less likely to be written and/or accepted for publication. In the Delphi, participants agreed most of the positive outcomes were core and very few negative. It may be that Delphi participants (particularly those who choose to dedicate hours of their own time) feel more positively about the outcomes than those that were invited but chose not to participate. This represents a risk of bias both in terms of an underreporting of negative outcomes and an inconsistent reporting of variables, with variables influencing outcomes being reported by people whose outcomes had been positive.

### Future research and recommendations

The core outcome set could be developed into a tool to assess outcomes. Measurement of learning outcomes is not straightforward, and self-report of learning is fraught with difficulties, including people not knowing what they do not know and people not being aware of what has changed for them at a particular time [39]. Nonetheless, metrics and standard indicators are useful for policy and decision-making [40], and this COS could facilitate quantification and the variables could facilitate hypothesis testing.

## Additional file

Additional file 1: Systematic review search criteria. Systematic review instructions for screeners. Systematic review results: table of literature included in the review. Systematic review and meta-synthesis results: table of outcomes. Systematic review and metasynthesis results: table of variables that may affect outcomes. List of core outcomes after Delphi study: percentage of consensus, positive (include)/negative (exclusive) and the overall rank in terms of stakeholder agreement. Descriptive statistics for each statement in the Delphi across the three rounds. (DOCX 143 kb)

## Abbreviations

CO: Core outcome
COS: Core outcome set
CPD: Continued professional development
HIC: High-income county
ILO: Intended learning outcome
LMIC: Low- and middle-income country
MOVE: MOVE Project, Measuring the outcomes of volunteering for education
NHS: National Health Service
