# Supplementary material for: The benefits of international volunteering in a low-resource setting: development of a core outcome set
Source: Hum Resour Health. 2018 Dec 20;16:69. doi: 10.1186/s12960-018-0333-5 (PMC6300912; doi:10.1186/s12960-018-0333-5)
Supplement: Supplementary file 1 — Systematic review search criteria. Systematic review instructions for screeners. Systematic review results: table of literature included in the review. Systematic review and meta-synthesis results: table of outcomes. Systematic review and metasynthesis results: table of variables that may affect outcomes. List of core outcomes after Delphi study: percentage of consensus, positive (include)/negative (exclusive) and the overall rank in terms of stakeholder agreement. Descriptive statistics for each statement in the Delphi across the three rounds. (DOCX 143 kb) [file 12960_2018_333_MOESM1_ESM.docx]

**Additional Files**

## 1: Systematic review search criteria

| **Cost/Benefit** | **What** | **Who** | **Where (Home)** | **Where (Away)** |
| --- | --- | --- | --- | --- |
| Impact | “Health Link” | Doctor | UK | Overseas |
| Impacts | “Health Links” | Doctors | “United Kingdom” | Foreign |
| Benefit | “Health Partnership” | Nurse | Britain | International |
| Benefits | “Health Partnerships” | Nurses | England | “Low Income Countries” |
| Cost | ‘International Placement’ | “Health Professional” | Scotland | “Low Income Country” |
| Costs | ‘overseas placement’ | “Health Professionals” | Wales | “Lower Middle Income Countries” |
| Outcome | volunteering | University | “Northern Ireland” | “Lower Middle Income Country” |
| Outcomes | Volunteer | Universities | British | “Developing Countries” |
| Evaluate | Placement | Hospital | English | “Developing Country” |
| Evaluation | volunteers | Hospitals | Scottish | “Global South” |
| Evaluations | Placements | “Health Institution” | Welsh | Africa |
| *‘Moderating variable*’* | ‘overseas placements’ | “Health Institutions” | “Northern Irish” | Asia |
| *‘Mediating variable*’* | ‘international placements’ | NHS |  | “South America” |
| *‘Influential factor*’* | “gap year” | Medical |  |  |
| *Factor* | “voluntary work” | Midwife |  |  |
| *Variable* | “volunteer project” | Physiotherapy |  |  |
| *Factors* |  | biomedical |  |  |
| *Variables* |  | Pharmacy |  |  |
| context |  | Therapist |  |  |
|  |  | Radiographer |  |  |
|  |  | radiography |  |  |
|  |  | Therapy |  |  |
|  |  | pharmacist |  |  |
|  |  | Podiatry |  |  |
|  |  | practitioner |  |  |
|  |  | Audiology |  |  |
|  |  | Orthotist |  |  |
|  |  | “healthcare scientists” |  |  |
|  |  | Dentists |  |  |
|  |  | Dentist |  |  |
|  |  | “nhs admin” |  |  |
|  |  | “nhs managers” |  |  |
|  |  | “nhs leaders” |  |  |
|  |  | “clinical Psychology” |  |  |
|  |  | Dental |  |  |
|  |  | “operating department” |  |  |
|  |  | “pharmacy technicians” |  |  |
|  |  | “health visitor” |  |  |
|  |  | “clinical support” |  |  |
|  |  | “healthcare worker” |  |  |
|  |  | healthcare |  |  |
|  |  | “clinical psychologist” |  |  |
|  |  | podiatrist |  |  |

(impact OR impacts OR benefit OR benefits OR cost OR costs OR outcome OR outcomes OR evaluation OR evaluate OR evaluations OR "moderating variable" OR "moderating variables" OR "mediating variable" OR "influential factor" OR "influential factors" OR factor OR factors OR variable OR variables OR context) AND ("health link" OR "health links" OR "health partnership" OR "health partnerships" OR "international placement" OR "international placements" OR "overseas placement" OR "overseas placements" OR "international volunteer" OR volunteer OR placement OR “gap year” OR “voluntary work” OR “voluntary project”) AND (doctor OR doctors OR nurse OR nurses OR "health professional" OR "health professionals" OR university OR universities OR hospital OR hospitals OR "health institution" OR "health institutions" OR nhs OR biomedical OR pharmacist OR pharmacists OR medical OR midwife OR midwives OR physiotherapist OR physiotherapy OR therapist OR Therapy OR Radiographer OR Radiography OR Podiatry OR Podiatrist OR practitioner OR audiologist OR audiology OR Orthotic OR prosthetics OR “healthcare scientist” OR Dentist OR Dental OR “NHS admin” OR “NHS manager” OR “NHS leader” OR “clinical psychology” OR “clinical psychologist” OR “operating department” OR “pharmacy technician” OR “health visitor” OR “clinical support” OR “healthcare worker” OR healthcare) AND (UK OR united kingdom OR britain OR england OR scotland OR wales OR "northern ireland" OR british OR english OR welsh OR scottish OR "northern irish") AND (overseas OR foreign OR international OR "low income country" OR "low income countries" OR "lower middle income country" OR "lower middle income countries" OR "developing country" OR "developing countries" OR "global south" OR Africa OR asia OR “south America”)

Database Search

| **Database** | **Number of hits** | **Number relevant** | **Date of Search** | **Other info** |
| --- | --- | --- | --- | --- |
| Medline (Pubmed) | 54 |  | 1/9/14 | Activated field title/abstract |
| Cochrane Economic Evaluations | 12 from trials, 0 from economic evaluations |  |  | Searched title, abstract and keywords  None relevant have not included database |
| Health Management Information Consortium | 0 |  |  | Was 0 in Jones article too |
| Health Business Elite | 45 |  |  | Searched EBSCO for abstracts only (unable to access specific databases but hosted by EBSCO) |
| Scopusr | 488 |  |  | Limited to UK as country and health professionals |
| Web of Knowledge (science) | 314 |  |  | Searched Topic field |
| PsychINFO | 0 |  |  | Was 0 in Jones article too |
| CINAHL | 45 |  |  |  |
| AMED | 0 |  |  | Was 0 in Jones article too |
| International Bibliography of Social Sciences, Social Services Abstracts and Sociological Abstracts | 6 |  |  | Only searched Abstracts |
| Global Health | 45 |  |  | Searched  EBSCO for abstracts only (unable to access specific databases but hosted by EBSCO) |
| JSTOR |  |  |  | Saying search criteria too long |

CINAHL:

http://search.ebscohost.com/login.aspx?direct=true&db=cin20&bquery=(impact+OR+impacts+OR+benefit+OR+benefits+OR+cost+OR+costs+OR+outcome+OR+outcomes+OR+evaluation+OR+evaluate+OR+evaluations+OR+%26quot%3bmoderating+variable%26quot%3b+OR+%26quot%3bmoderating+variables%26quot%3b+OR+%26quot%3bmediating+variable%26quot%3b+OR+%26quot%3binfluential+factor%26quot%3b+OR+%26quot%3binfluential+factors%26quot%3b+OR+factor+OR+factors+OR+variable+OR+variables+OR+context)+AND+(%26quot%3bhealth+link%26quot%3b+OR+%26quot%3bhealth+links%26quot%3b+OR+%26quot%3bhealth+partnership%26quot%3b+OR+%26quot%3bhealth+partnerships%26quot%3b+OR+%26quot%3binternational+placement%26quot%3b+OR+%26quot%3binternational+placements%26quot%3b+OR+%26quot%3boverseas+placement%26quot%3b+OR+%26quot%3boverseas+placements%26quot%3b+OR+%26quot%3binternational+volunteer%26quot%3b+OR+volunteer+OR+placement+OR+%26quot%3bgap+year%26quot%3b+OR+%26quot%3bvoluntary+work%26quot%3b+OR+%26quot%3bvoluntary+project%26quot%3b)+AND+(doctor+OR+doctors+OR+nurse+OR+nurses+OR+%26quot%3bhealth+professional%26quot%3b+OR+%26quot%3bhealth+professionals%26quot%3b+OR+university+OR+universities+OR+hospital+OR+hospitals+OR+%26quot%3bhealth+institution%26quot%3b+OR+%26quot%3bhealth+institutions%26quot%3b+OR+nhs+OR+biomedical+OR+pharmacist+OR+pharmacists+OR+medical+OR+midwife+OR+midwives+OR+physiotherapist+OR+physiotherapy+OR+therapist+OR+Therapy+OR+Radiographer+OR+Radiography+OR+Podiatry+OR+Podiatrist+OR+practitioner+OR+audiologist+OR+audiology+OR+Orthotist+OR+prostethics+OR+%26quot%3bhealthcare+scientist%26quot%3b+OR+Dentist+OR+Dental+OR+%26quot%3bNHS+admin%26quot%3b+OR+%26quot%3bNHS+manager%26quot%3b+OR+%26quot%3bNHS+leader%26quot%3b+OR+%26quot%3bclinical+psychology%26quot%3b+OR+%26quot%3bclinical+psychologist%26quot%3b+OR+%26quot%3boperating+department%26quot%3b+OR+%26quot%3bpharmacy+technician%26quot%3b+OR+%26quot%3bhealth+visitor%26quot%3b+OR+%26quot%3bclinical+support%26quot%3b+OR+%26quot%3bhealthcare+worker%26quot%3b+OR+healthcare)+AND+(UK+OR+united+kingdom+OR+britain+OR+england+OR+scotland+OR+wales+OR+%26quot%3bnorthern+ireland%26quot%3b+OR+british+OR+english+OR+welsh+OR+scottish+OR+%26quot%3bnorthern+irish%26quot%3b)+AND+(overseas+OR+foreign+OR+international+OR+%26quot%3blow+income+country%26quot%3b+OR+%26quot%3blow+income+countries%26quot%3b+OR+%26quot%3blower+middle+income+country%26quot%3b+OR+%26quot%3blower+middle+income+countries%26quot%3b+OR+%26quot%3bdeveloping+country%26quot%3b+OR+%26quot%3bdeveloping+countries%26quot%3b+OR+%26quot%3bglobal+south%26quot%3b+OR+Africa+OR+asia+OR+%26quot%3bsouth+America%26quot%3b)&type=1&site=ehost-live

## 2: Systematic review instructions

**Screening Process**

When given the list of search results, check that each paper meets the inclusion/exclusion criteria and complete the corresponding spreadsheet. In order for a paper to be included it should meet all the criteria.

**Database**

Please record the name of the database searched to find the article (i.e. SCOPUS). If it appears in more than one please list all.

**Title**

Please record the full title

**Authors**

Please record all authors

**Year**

Please record the year of publication

**UK**

Please answer Yes or No. Are participants from a UK organisation/residing in the UK directly before placement/Employed by NHS/UK nationals?

UK includes: English, Scotland, Wales and Northern Ireland

**Health Focus**

Please answer Yes or No. Do the activities have a health focus? This could extend beyond typical patient care (Doctors and Nurses) for example to biomedical engineers, hospital lab assistants, ODPs, physiotherapists, etc.

**Lower/Lower-Middle income country**

Please answer Yes or No. Are the activities taking place in Lower/Lower-middle income (developing) country? Please answer No if the receiving country is high income (developed).

**Volunteer (no salary)**

Please answer Yes or No. Are the participant’s volunteers so not in receipt of a full salary?

**Benefits/Costs/Variables**

Please answer Yes or No. Is there reference to benefits, costs or variables? This can be at an individual (participant) level, or an organisational level (ie. NHS). Benefits/costs/variables must be in reference to the developed country/organisation/individual. Please answer No if benefits/costs/variables only relate to the receiving (developing) country.

**Primary Data**

Please answer yes or no. Does the paper contain primary data? Only answer yes if the data is Levels I – III in the following table:

| **Level of Evidence** | **Description** |
| --- | --- |
| Level I | Based on randomized, controlled trials (or meta-analysis of such trials) of adequate size to ensure a  low risk of incorporating false-positive or false-negative results |
| Level II | Based on randomized, controlled trials that are too small to provide Level I evidence. These may  show either positive trends that are not statistically significant or no trends and are associated with  a high risk of false-negative results |
| Level III | Based on non-randomized, controlled or cohort studies, case series, case controlled studies, or  cross-sectional studies |
| Level IV | Based on the opinion of respected authorities or that of an expert committee as indicated in  published consensus conferences or guidelines |
| Level V (a) | Based on the opinion of those individuals who have knowledge in one particular field and are  applying that knowledge to another field; or summarizes the collective wisdom or experiences of  others in the field |
| Level V (b) | Based on the opinion of those individuals who have written and reviewed the guidelines, based on  their experience, knowledge of the relevant literature, and discussion with their peers |

A quote from a qualitative study with distinct methodology of interviewing participants to collect data would be an example of primary data.

A quote from an expert in the field that has not been collected through a distinct methodological framework would not be an example of primary data.

**Data Extraction Spreadsheet**

**Study/Journal**

Title, Author, Year same as in Screening Instructions

**Journal/Source**

Please record the name of the journal/source in which the paper/article in published

**Volume/Page Number**

Please record the volume and page number

**Language**

Please record the language used. This review does not exclude foreign language articles.

**Participant Characteristics**

**No of Participants**

If the paper presents this information, please record the number of participants tested

**Participant Age**

If the paper presents this information, please record mean age and age range of participants

**Participant Gender**

If the paper presents this information please record the number of participants of each gender

**Profession**

If the paper presents this information please record the profession/professions of the participants

**Stage in Career**

If the paper presents this information please record the stage of the career which the participants are at (Undergraduate Student, Postgraduate Student, Consultant etc.)

**Country of Origin**

Please record the country from which the participant originated. The country of origin would be the place of residence directly before the placement. For example, if a participant was originally born in Somalia, but had spent the last few years working for the NHS (ahead of the placement) the country of origin would still be UK.

**Receiving Country**

Please record the country in which in the participant is volunteering**.**

**Length of Stay**

Please record the length of stay in the receiving country.

**Sending Organisation**

Please record the organisation that has arranged the placement. In some cases volunteers are independent (please record *independent*). This is likely to be a health partnership, a NGO, or a profit-making company (ie. original volunteers, STA travel).

**Methodology**

**Quant/Qual**

Please record whether the study uses quantitative methods, qualitative methods or both

**Procedural Details**

Please list all Procedural Methodology, (ie. Interview, questionnaire etc.) Also anything specific to the study (ie. conditions, trials etc.)

**Level of Evidence**

Please record the Level of Evidence based on the below table:

| **Level of Evidence** | **Description** |
| --- | --- |
| Level I | Based on randomized, controlled trials (or meta-analysis of such trials) of adequate size to ensure a  low risk of incorporating false-positive or false-negative results |
| Level II | Based on randomized, controlled trials that are too small to provide Level I evidence. These may  show either positive trends that are not statistically significant or no trends and are associated with  a high risk of false-negative results |
| Level III | Based on non-randomized, controlled or cohort studies, case series, case controlled studies, or  cross-sectional studies |

**Outcomes**

In each column please record the outcome extracted from the data. If the author includes any further information about how they reached their conclusion in regards to the specified outcome please also record this information, for example:

- Ability to communicate effectively *(reached through initial codes of ‘able to understand the needs of others’, ‘understanding the necessity if listening’)*
- Increased tropical disease knowledge in the NHS *(authors decided on this category as individuals reported developing new knowledge which was very useful upon return to the UK)*

Please ensure every outcome at any level that is presented in the data is recorded.

Outcomes must be extracted from the results/discussion section. For example if an outcome is mentioned in the introduction but is not present in the results, this would not be recorded.

If a paper presents more than 10 outcomes please insert extra columns accordingly.

**Variables**

Please record any variables (both moderating and mediating) that affect outcomes. Examples of this could be gender, length of stay, receiving country etc.)

Please record any extra information that is related to the variable and if it relates to a specific outcome. For example:

- Gender (males are more likely to gain communication skills than females)
- Length of stay (those who stay longer than 6 months have a greater understanding of tropical disease)

These must be extracted from the results/discussion section. For example if a variable is mentioned in the introduction but is not present in the results, this would not be recorded.

If a paper presents more than 10 variables please insert extra columns accordingly.

**Costs**

Please record any costs that are experienced by the individual or the UK/organisation.

Please record the cost as well as any important information/variables presented regarding the cost, for example:

- Skill Loss (participants complain that they experience a technical skill loss, but this is counteracted by a non-technical skill gain and is usually overcome within weeks of being back in the NHS)
- NHS staffing issues (when people leave for a short period, temporary staff must be sourced to cover leave, this is costly, difficult and time-consuming)

These must be extracted from the results/discussion section. For example if a cost is mentioned in the introduction but is not present in the results, this would not be recorded.

If a paper presents more than 10 costs please insert extra columns accordingly

## 3: Systematic Review Results: Table of literature included in the review

|  | Title | Date | Authors | LMICs | Profession | Level of Evidence |
| --- | --- | --- | --- | --- | --- | --- |
| 1 | Hands across the Equator: the Hereford/Muheza link | 1988 | Wood JB, Hills EA | Tanzania | Uncategorised | VI |
| 2 | Hands across the Equator : Hereford/Muheza link 8 years on | 1994 | Wood JB, Hills EA, Keto FJ | Tanzania | Uncategorised | VI |
| 3 | Training for health care in developing countries: the work of the Tropical Health and Education Trust | 1998 | Parry E, Parry V | Uncategorised | Uncategorised | Vb |
| 4 | A comparison of an international experience for nursing students in developed and developing countries | 2000 | Thompson K, Boore J, Deeny P | Australia Brazil Canada Gambia New Zealand India Spain Kenya Sweden Malawi USA Tanzania Uganda Zambia | Nurses | III |
| 5 | Education and research links between the UK and Thailand | 2000 | Burnard P, Claewplodtook P, Pathanapong P | Thailand | Nurses | Vb |
| 6 | UROLINK – benefits for trainees from both sides | 2002 | Gujral S, Nassanga R | Tanzania | Urologists | Vb |
| 7 | Twinning: the future for sustainable collaboration | 2002 | MacDonagh R, Jiddawi M, Parry V | Tanzania | Urologists | Vb |
| 8 | The impact of international placements on nurses' personal and professional lives: literature review. | 2005 | Button L, Green B, Tengnah C, Johansson I, Baker C | Uncategorised | Student Nurses | III |
| 9 | International Health Electives: Four years of experience | 2005 | Miranda JJ, Yudkin JS , Willott C | Uncategorised | Medical students | III |
| 10 | NHS Links: a new approach to international health links | 2005 | Wright J, Silverman M, Sloan J | Uncategorised | Uncategorised | Vb |
| 11 | Can you help? | 2006 | Hancock C | Africa (mainly East) | Nurses | Vb |
| 12 | International health links: an evaluation of partnerships between health-care organizations in the UK and developing countries. | 2006 | Baguley D, Killeen T, Wright J | Ethiopia, Bangladesh, Botswana, Guyana, Nepal, Tanzania, Thailand and Uganda | Uncategorised | III |
| 13 | The contribution of international health volunteers to the health workforce in sub-Saharan Africa | 2007 | Laleman G, Kegels G, Marchal B, Van der Roost D, Bogaert I, Van Damme W | Sub-Saharan Africa | Health Volunteers | III |
| 14 | Nursing electives: an innovative and creative learning opportunity. | 2008 | Peate I | Uncategorised | Nursing Student | Vb |
| 15 | Uncovering study abroad: Foreignness and its relevance to nurse education and cultural competence | 2008 | Greatex White S | Uncategorised | Nursing students | III |
| 16 | Short-term visits by eye care professionals: ensuring greater benefit to the host community | 2008 | Pyott A | Uncategorised | Orthomologists | Vb |
| 17 | Overseas Placements: Addressing Our Challenges? | 2008 | Clampin A | Uncategorised | Student Occupational Therapists | Vb |
| 18 | A questionnaire study of Voluntary Service Overseas (VSO) volunteers: health risk and problems encountered. | 2009 | Bhatta P, Simkhada P, van Teijlingen E, Maybin S | Uncategorised | 10.1% Drs and Nurses | III |
| 19 | All aboard with Impact India | 2009 | Sikkh N | India | Dentists | Vb |
| 20 | Internationalising occupational therapy education | 2009 | Horton A | Uncategorised | Student Occupational Therapists | Vb |
| 21 | Electives: isn't it time for a change? | 2009 | Dowell J, Merrylees N | Uncategorised | Medical Students | Vb |
| 22 | NHS links: achievements of a scheme between one London mental health trust and Uganda | 2009 | **Baillie D, Boardman J, Onen T, Hall C, Gedde M, Parry E** | Uganda | Mental Health Professionals | Vb |
| 23 | Global health partnerships: leadership development for a purpose | 2009 | Hockey P, Tobin A, Kemp J, Kerrigan J, Kitsell F, Green P, Sewell A, Smith C, Stanwick S, Lees P | Cambodia | Healthcare workers | Vb |
| 24 | Research into practice: 10 years of international public health partnership between the UK and Swaziland. | 2010 | Wright J, Walley J, Philip A, Petros H, Ford H | Swaziland | NHS staff and Academics | Vb |
| 25 | Medical electives: a chance for international health | 2010 | Banerjee | Uncategorised | Medical Students | Vb |
| 26 | International Health Links movement expands in the United Kingdom | 2010 | Leather A, Butterfield C, Peachey K, Silvermand M, Syed Sheriff R | Various Developing countries | Uncategorised | Vb |
| 27 | Global public health training in the UK: preparing for the future | 2011 | [Lee AC](http://www.ncbi.nlm.nih.gov/pubmed/?term=Lee%20AC%5BAuthor%5D&cauthor=true&cauthor_uid=21389106), [Hall JA](http://www.ncbi.nlm.nih.gov/pubmed/?term=Hall%20JA%5BAuthor%5D&cauthor=true&cauthor_uid=21389106), [Mandeville KL](http://www.ncbi.nlm.nih.gov/pubmed/?term=Mandeville%20KL%5BAuthor%5D&cauthor=true&cauthor_uid=21389106) | Uncategorised | Registrars | III |
| 28 | Reflecting on the learning experiences of student nurses in rural Uganda. | 2011 | Lovett W, Gidman J | Uganda | Health Visitor’s/ Student Nurses | Vb |
| 29 | Working with UK-based non-governmental organisations for better reproductive health in Nepal | 2011 | Nunns D | Nepal | Various | Vb |
| 30 | Student nurse perceptions of risk in relation to international placements: A phenomenological research study. | 2012 | Morgan DA | High Income Countries-9, Low Income Countries- 1 | Student Nurses | III |
| 31 | Travel related illness in short-term volunteers from the UK to developing countries. | 2012 | Wyler N, Green S, Boddington N, Davies C, Friedli K, Lankester T | Developing Countries | Healthcare Professionals (others such as teaching, construction etc also included) | III |
| 32 | The role of health links in international development: the need for greater evidence? | 2012 | Smith C | NA | Uncategorised | Vb |
| 33 | How international health links can help the NHS workforce develop | 2012 | Longstaff B | Uncategorised | Uncategorised | III |
| 34 | Combining UK general practice with international work — who benefits? | 2012 | [Seo HN](http://www.ncbi.nlm.nih.gov/pubmed/?term=Seo%20HN%5BAuthor%5D&cauthor=true&cauthor_uid=23265233), [Smith C](http://www.ncbi.nlm.nih.gov/pubmed/?term=Smith%20C%5BAuthor%5D&cauthor=true&cauthor_uid=23265233), [Pettigrew LM](http://www.ncbi.nlm.nih.gov/pubmed/?term=Pettigrew%20LM%5BAuthor%5D&cauthor=true&cauthor_uid=23265233), [Dorward J](http://www.ncbi.nlm.nih.gov/pubmed/?term=Dorward%20J%5BAuthor%5D&cauthor=true&cauthor_uid=23265233), [Bygrave H](http://www.ncbi.nlm.nih.gov/pubmed/?term=Bygrave%20H%5BAuthor%5D&cauthor=true&cauthor_uid=23265233) | Uncategorised | GPs | III |
| 35 | Combining general practice with international work: online survey of experiences of UK GPs | 2012 | Smith C, Pettigrew LM, Seo HN, and Dorward J | Uncategorised | GPs | III |
| 36 | A new partnership for anesthesia training in Zambia: reflections on the first | 2013 | Kinnear JA, Bould MD, Ismailova F, Measures E | Zambia | Consultant Anaesthesiologists | Vb |
| 37 | Factors that influence a career choice in primary care among medical students from high-, middle-, and low-income countries: a systematic review. | 2013 | Puertas EB, Arósquipa C, Gutiérrez D | Uncategorised | Medical Students | III |
| 38 | Becoming culturally sensitive: A painful process? | 2013 | Briscoe L | UK America Canada Guatemala | Midwifery Students | III |
| 39 | 'Tanzania changed me'. | 2013 | Dean E | Tanzania | Nurses | Vb |
| 40 | Do health partnerships with organisations in lower income countries benefit the UK partner? A review of the literature. | 2013 | Jones FA, Knights DP, Sinclair VF, Baraitser P | Uncategorised | Healthcare Professionals | III |
| 41 | Placements in global health masters’ programmes: what is the student experience? | 2013 | Cole DC, Plugge EH, Jackson SF | Uncategorised | Global health Masters Students | III |
| 42 | Should trainee doctors use the developing world to gain clinical experience? The annual Varsity Medical Debate – London, Friday 20th January, 2012 | 2013 | Gilbert BJ, Miller C, Corrick F, Watson RA | Developing Countries | Trainee Doctors | Vb |
| 43 | Maximising the value from the elective experience: post-elective workshops | 2013 | Evans R, Dotchin C, WalkerR | Uncategorised | Medical Students | III |
| 44 | Developing cultural sensitivity and awareness in nursing overseas | 2014 | Paterson JG | Uncategorised | Nurses | Vb |
| 45 | Sharing skills in dementia care with staff overseas. | 2014 | Marçal-Grilo J | Sri Lanka | Nurses | Vb |
| 46 | The benefits for children's nurses of overseas placements: where is the evidence? | 2014 | Standage R, Randall D | India, Canada, USA | Children’s Nurse Students | III |
| 47 | Making short-term international medical volunteer placements work: a qualitative study | 2014 | Elnawawy O, Lee AC, Pohl G | Nepal | GPs | III |
| 48 | Evaluation of effect on skills of GP trainees taking time out of programme (OOP) in developing countries. | 2014 | Kiernan P, O'Dempsey T, Kwalombota, K Elliot L, Cowan L | South Africa, Zambia and Rajasthan (as well as allowing them to construct their own) | GPs | III |
| 49 | Mutual learning and reverse innovation–where next? | 2014 | Crisp N | Uncategorised | Uncategorised | Vb |
| 50 | Boost or burden? Issues posed by short placements in resource-poor settings | 2014 | Dowell J, Blacklock C, Liao C, Merrylees N | Uncategorised | Uncategorised | Vb |
| 51 | Medical professionalism across cultures: A challenge for medicine and medical educatio | 2014 | Jha V, Mclean M, Gibbs TJ, Sandar J | Various (developed and developing) | Medics | Vb |
| 52 | Lessons from an elective in Sierra Leone | 2014 | Robinson T | Sierra Leone | Medical Students | Vb |
| 53 | Supporting medical students to do international field research: a case study | 2014 | Pearson S, Parr J Ullah Z, Omar M | Uncategorised | Medical Students, Academics, | III |
| 54 | Electives in undergraduate medical education: AMEE Guide No. 88 | 2014 | Lumb A, Murdoch-Eaton D | Uncategorised | Medical Students | Vb |
| 55 | International work and leadership in UK general practice. | 2014 | Young P, Smith C., Pettigrew L. Seo, HN, Blane D | Uncategorised | GPs | III |

## 4: Systematic Review and Metasynthesis Results: Table of Outcomes

| **Outcome** | **Examples or components** |
| --- | --- |
| **Knowledge** | |
| Increased awareness of and knowledge about how communication between two people can affect understanding | Effectively conveying ideas in an contextually appropriate way |
| Increased awareness of and knowledge about conditions and procedures rarely encountered in the UK | Greater knowledge of procedures not used in the UK  Better management of conditions that are not common in the UK |
| Increased awareness of and knowledge about the importance of assessing healthcare on an individual basis | The uniqueness of each patient |
| Increased awareness of and knowledge about the importance of community participation in health | The importance of community involvement in health  Awareness of the role of the community in improving healthcare  Understanding the importance of community work |
| Increased understanding of basic skills and ideas | Core skills often replaced by technology (basic observations, using eyes, relying less on lab tests) |
| Increased awareness of and knowledge about clinical knowledge in relation to other professions | Doctors about nurses and vice versa |
| Increased awareness of and knowledge about the importance of mutual learning and respect |  |
| Understanding how to be a good teacher | Understanding how to target training most effectively  Ability to suggest and acknowledge improvements in teaching  Understanding importance of experiential learning |
| Increased awareness of and knowledge about the importance of relationship maintenance skills | Consciously making an effort to get on with colleagues  Learning colleagues names |
| Increased awareness of and knowledge about the positive impact of clinical policies and governance | Greater policy skills |
| Increased awareness of and knowledge about tropical diseases | New knowledge of tropical diseases and increasing existing knowledge |
| Increased awareness of and knowledge about appropriate clinical behaviour | Knowing when to ask for help  Knowledge of different populations needs |
| Increased awareness of and knowledge about the cultural aspects of health | Greater understanding and appreciation of health promotion  Understanding how culture affects daily occupation  Increased understanding of cultural differences in health  Understanding the effects of politics on health  Understanding how culture affects you professionally  Understanding how to incorporate health beliefs into a shared decision  Greater understanding of sustainable healthcare |
| Increased awareness of and knowledge about global issues | Re-evaluation of world issues  Deeper engagement with issues of equality and diversity  Greater global knowledge |
| Increased awareness of and knowledge about cultural differences and similarities | Understanding key issues within a culture Understanding culturally acceptable behaviour Learning about other cultures  Being more attentive to subtle clues about cultural differences  Accepting cultural differences  Understanding of cultures of UK immigrants, Changed assumptions of culture |
| Increased awareness of and knowledge about ethical considerations | Through experiential learning |
| Increased awareness of and knowledge about the need for/importance of training | Understanding how important effective training is in the UK and overseas |
| Increased awareness of and knowledge about how other healthcare systems function | Developed insight into disparities within healthcare systems  Increased understanding and awareness of other systems |
| Increased self-awareness | Awareness of own skills and limitations  Able to challenge own beliefs  Able to reflect on own situation  Able to self-define |
| Increased awareness of and knowledge about finance in healthcare | Awareness of the costs of healthcare |
| Increased awareness of and knowledge about the resistance of culture | Understanding how to make small changes  Being innovative in overcoming language and cultural difference  Understanding not to enforce your perspective onto others |
| Increased awareness of and knowledge about culture in practical assessments | Understanding importance of collecting relevant cultural information about people’s presenting health problems  Learning how to conduct cultural assessments and culturally based physical assessments |
| Increased awareness of and knowledge about the importance of trust within healthcare systems and staff | Understanding other peoples perceptions of trust |
| Increased awareness of and knowledge about how systems work | Able to identify stakeholders and change agents Awareness of value systems  Understanding influencing patterns of those in power  Ability to assess impact of healthcare systems Understanding the difficulty of questioning an organisation |
| **Skills** | |
| Ability to overcome communication challenges | Liase between groups  Engage senior people  Negotiate with senior people |
| Ability to communicate non-verbally | Developed non-verbal techniques |
| Ability to provide better care | Ability to provide multicultural care  Ability to develop most effective approaches to care  Taking responsibility for providing quality care |
| Ability to observe and examine patients | Increased intuitive knowledge of clinical signs  Ability to make diagnosis without investigations Increased clinical judgement |
| Ability to be innovative with clinical skills | Use of innovative techniques  New ways of working) |
| Ability to use a broader range of clinical skills | Enhancing existing skills and acquiring new clinical skill |
| Ability to apply clinical skills to another context | A more challenging environment or a low resource setting |
| Ability to work with limited resources | Being more resourceful  Ability to target resource  Ability to find solutions despite limited resources  Ability to work without reliance on technology  Ability to manage in a low resource setting Understanding the reasons behind lack of resources |
| Ability to ‘get the most out of people’ | Encouraging people to work together  Empowering people to recognise their own strengths and to take possession of their own work/projects  Ability to assess the capability of others  Encouraging people to work together |
| Ability to manage risk | Manage risk in advance  Evaluation of environment  Understanding the clinical importance of risk management  Understanding the wider implication of poorly managed risk |
| Able to negotiate with multiple stakeholders |  |
| Ability to make independent clinical decisions | Ability to make an urgent decision in an emergency  Dealing with uncertain outcomes |
| Ability to manage time and prioritise | Ability to respond quickly in an emergency Prioritisation of limited resources |
| Ability to work within a system with unfamiliar power systems |  |
| Ability to fulfil future leadership roles |  |
| Ability to plan and organise | Able to set direction |
| Ability to improve service | Including renewed enthusiasm for service improvement |
| Ability to transfer skills and knowledge to another context |  |
| Ability to work towards solutions | Solution focused approach |
| Ability to find facts to solve problems |  |
| Ability to make decisions | Understanding who the decision is for  Taking action on decision  Make judgements |
| Ability to co-operate |  |
| Ability to work as part of a team | Understanding team group norm  Perception of roles within the group  Managing personal objectives within a group |
| Ability to develop friendships | Relationship formation skills  Developing new friendships |
| Ability to build a global network |  |
| Ability to give and accept praise |  |
| Ability to disseminate best practice globally |  |
| Ability to be professionally competent | Wider view of profession  Intellectual development  Reminder of professional responsibilities  Stronger work ethic |
| Developed research skills | Grant application skills  Greater research skills |
| Ability to present work | Greater presentation skills |
| Ability to write reports and academic pieces |  |
| Ability to apply knowledge gained in host system to the UK | Relating experiences back to UK,  Using knowledge gained overseas to improve UK systems |
| Ability to cope | Better coping strategies  Ability to deal with knock backs  Being unfazed by things  Learning to deal with stress |
| Ability to adapt social norms to meet needs of another culture | Change behaviour to fit with social norms |
| Ability to lead by example |  |
| Ability to exchange ideas with those from another culture | Communicate effectively with those from another country or culture |
| Ability to encourage others to take responsibility for own health |  |
| Ability to manage self | Own expectations  Self-reliance  Self-management  Self-assurance |
| Ability to manage projects |  |
| Ability to think through problems in a logical way | Analytical thinking  Lateral thinking |
| Ability to establish communication systems | Formal and informal |
| Developed teaching skills | Greater training delivery skills |
| Ability to use evidence based practice | Ability to apply theory |
| Ability to speak host language |  |
| **Attitudes** | |
| Confidence to work in other locations | Confidence to move to another city/country  Working with UK multicultural/ underserved populations |
| Independence |  |
| Integrity |  |
| Diplomacy |  |
| Humility |  |
| Judgement | Non-judgemental attitude  Changed self-judgement |
| Proactivity | Using initiative |
| Increased cultural sensitivity | Sensitivity to reasoning behind cultural differences  Sensitivity towards feelings of minority Sensitivity towards language barriers |
| Increased respect for other cultures |  |
| Reinforced ethnic and cultural identity | Positivity about being British |
| Patience and tolerance | Accepting and working at other peoples pace More tolerance |
| Increased confidence | In caring for clients from another culture  In quality improvement methods  To take bolder steps  Self-confidence  Confidence in professional ability  In ability to address challenging situations |
| Flexibility and adaptability | Acceptance of other ways of working  Adaptation to responsibility  Able to adapt more easily to unfamiliar situations  Able to cope more easily with change  Able to manage change  Gaining a wider perspective  Understanding the flexibility of roles |
| Emotional intelligence | Changed engagement with self  Knowledge and world |
| Appreciation of importance of care and compassion | Empathy |
| Changed perception of otherness | Understanding importance of being a friendly stranger in UK  Experienced feeling like a foreigner whilst away |
| Appreciation of excellent human resource in the NHS | Multidisciplinary teams  HR structures,  Appreciation of own profession  Understanding hierarchy and the importance of each person within it  Interaction between healthcare professionals |
| Appreciation of having the right tools and equipment to be able to do the job | Resources: technical equipment, disposal equipment, cleaning products and protective equipment |
| Appreciation of free universal health | NHS system of free healthcare for all  Privilege and opportunity for UK citizens, Understanding the expectations that are placed on NHS by service users |
| Appreciation of clinical governance procedures within NHS | Waste disposal  Audit  Teamwork  Education system  Tests and investigations  Understanding that systems are not restricting |
| **Organisational Outcomes** | |
| Increased staff knowledge and skills | Increased staff knowledge of low cost healthcare,  More knowledgeable staff  Staff able to discover better ways of doing things  Staff more aware of waste reduction |
| Increased international reputation of NHS | Greater fulfilment of social responsibility) |
| NHS becomes a more attractive employee (If offers staff opportunity to volunteer) |  |
| Increased patient satisfaction | Staff better able to respond to UK multicultural populations  Staff have greater relationships with multicultural patient population  Staff more in tune with patients  Staff more aware of individual needs of patients |
| Medical school more attractive to students (if allow students to go abroad) |  |
| Increased workforce productivity |  |
| Reduction in NHS drop outs | Increased staff retention |
| Increased international reputation (of UK) |  |
| **Miscellaneous outcomes** | |
| Upper hand when competing for careers |  |
| Increased job satisfaction | Increased motivation and morale with profession  Renewed passion for work  Sense of reward |
| Influence career pathway | Affects specialism choice  Exploration of potential career pathways  Persuing careers in primary care, family practice, and public service  Sub-specialism in global health,  Teaching or lecturing careers  Teaching responsibilities within clinical position |
| Refreshment and reinvigoration | Coming back to UK refreshed and reinvigorated  Bringing new ideas to UK |
| Personal satisfaction | Personal achievements and challenges  New experiences  Experiencing a different lifestyle  Aa holiday  Personal fulfilment |
| Increased motivation to learn a language |  |
| Development of a new perspective | Revising assumptions  Reassessed outlook on life  Seeing things differently  Changed world views  Changed outlook  Look at everything in a new light  Openness to new experiences  Put things into perspective |
| Escapism | Escape from agendas and workload  A chance to take time out of training and practice  Space to think and clarify career objectives |
| **Negative Outcomes** | |
| Costs to British patients | Bringing tropical illness to UK |
| Developing redundant or bad skills/attitudes | Non-transferable skills  Bad habits  Deskilling  Overconfidence in ability  Poorer communication skills  Loss of confidence |
| Difficulty getting the job you want on return | Permanent jobs or training contracts |
| Loss of trained staff | Utilisation of key staff time  Financial cost of losing staff  Having to find cover for staff |
| Negative perceptions of NHS | Reputational  When program run badly |
| Distracted staff |  |
| Exposure to ethical dilemmas | To work outside of competency  Lack of regulation  Too much responsibility |
| No recognition of accreditation upon return |  |
| Reduced experience and exposure to  UK procedures, protocols and research | No experience with NHS procedures that don’t exist in host country  Missing out on formal training and conferences  No experience with chronic disease management over time  No experience with health conditions that are common in UK and not in host country,  Unaware of NHS protocol and updates  Loss of professional networks and relationships |
| Affects professional progression | Lengthens training  Less time to prepare for exams  Loss of partnerships |
| Negative colleague perceptions | Colleagues have to cover |
| Use of time | Annual leave  General time consumption |
| Professional revalidation issues | For consultants |
| Litigation | Legal issues involving clinical/professional risk |
| Security | Exposure to aggression  Violence and death  Becoming a victim of crime  Political unrest |
| Carbon footprint |  |
| Culture shock |  |
| Environmental and infrastructural risk |  |
| Extreme nationalism towards UK |  |
| Experiencing negative feelings | Feeling as though imposing on UK colleagues to provide cover  Feeling out of depth  Frustration  Guilt and regret about death |
| Financial loss | Costs of getting involved  Loss of earnings  Loss of pension or employment entitlement |
| Health consequences | Animal bites  Tropical diseases  Sexually Transmitted Disease  Injuries and transport accidents  Infection  Jet lag  Skin disease |
| Psychological consequences | Depression  Anxiety  Stress  Nervousness |
| Exhaustion and burn out |  |
| Loneliness | Isolation  Social isolation  No or few friends in host country |
| Missing things at home | Missing life in the UK  Time away from family and friends |
| Loss of interest in global health and international placements | Negative perceptions of volunteering and international placements |
| Socio-cultural risk | Exposure to corruption  Experiencing resistance to western influence |
| Become judgemental |  |

## 5: Systematic Review and Metasynthesis Results: Table of variables that may affect outcomes

| **Variable** | **Examples/Components** |
| --- | --- |
| **External Variables** | |
| Ethics | Are local patients informed of the risk?  Corporate and social responsibility  Do patients come first?  Levels of standards  Health and Safety |
| Funding | Consistency of funding for project  Finance plan for project  Funding from a charity or grant  Volunteer funded by sending organisation  Volunteer fundraising  Support of a health link partnership  Self-funding  Specific funding for training |
| Decision of host countries needs | Needs Assessment by both parties  High income party decides  Host country decides |
| Healthcare facility factors | Does the environment favour flexibility  Does management allow people to become multi-skilled  Level of organisational support  Use of specific activities/sessions for learning  Volunteer exposure to numerous systems  Opportunities for exposure to culture outside of hospital  Differences in protocols  Licensing and professional regulations  Level of corruption  Are volunteer skills best utilised?  Encouragement and motivation of volunteers  Financial and human resources  Criticism of project/volunteers  Mobility of local staff  Existence of local role models  Number of times volunteers and local professionals engage |
| Benefits for host organisation | Donations  Material/financial benefits  Payment for supervision |
| Income of host country | Low  Middle  High |
| Commitment of local staff to project | Staff time pressures  Empowerment of local staff  Involvement of hospital leaders  Project use local experts  Local perceptions of volunteers  Value of volunteer opinions |
| Difference between host and origin country | Cultural distance between host and origin country  Level of cultural immersion  Severity of communication difficulties  Shared values and cultural fit |
| NHS and UK Factors | Accreditation  Existence of returner schemes  Bureaucracy  Political Climate in UK  Recognition of benefits by NHS/UK organisation  Trust, deaneries and PCT’s support and influence  Support of UK colleagues |
| Relationship between host and sending organisation | Dependence on one-another  Quality of communication  Collaboration  Differing expectations  Equality of input  Ground rules and protocol  How the link is set up  Multi-departmental partnerships  Registered links i.e. THET  Sensitivity to local contexts  Sustainability of relationship  Length of relationship  Uni-professional or multi-disciplinary |
| Level of supervision and support | Mentor in UK  Support in UK  Supervision from western staff residing in host country  Linking of senior and junior volunteers  Supervision from local people  Support structure in host country  Access to HR |
| Existence of other similar project in areas | Over-crowding of volunteers in one hospitals  Support from others volunteers in another project |
| Focus of project | Agreement of focus  Focus on mutual benefit  Alignment of project with host country health plans  Capacity building focus  Service delivery focus  Developmental focus  Sustainability focus  Training focus |
| Practical Factors | Travel  Accommodation  Use of travel agent  Documentation |
| Structure of the programme | Aims developed by volunteers themselves  Informed by other similar projects  Informed by literature  Coercion  Continuation of project by other volunteers  Involvement of local governments  Countrywide initiatives  Do volunteers have a project?  How project is managed (i.e., well run)  Existence of guidelines and frameworks  Commitment/time allocation/number of UK admin staff  Programme tailored to volunteer needs  Spread of volunteers throughout the year  Quality control of services provided by volunteers |
| Length of placement | Long term  Short term  Adjustment  Short re-occurring trips |
| Project evaluations | Evaluations during placement  Post-placement longitudinal evaluation |
| Project retention and recruitment of volunteers | Volunteer drop out  How are volunteers recruited |
| Assessment and Education | Existence of set learning outcomes and objectives  Use of assessment  Use of model to facilitate contextual understanding |
| Time of programme arrangement | In advance  In country |
| Training and preparation | Appropriate training and preparation before placement  Contact with previous volunteers  Debriefing  Encouraging people to share experience  Set training and preparation events  Health monitoring  Meeting in UK  Training and preparation in country  Volunteer involvement in planning |
| Type of organisation | Health Partnership  Existing organisations  Commercial involvement  DIY/self-organised  Remote or physical volunteering |
| Transferability of skills learnt | Non-transferable skills  Skills latency period  Context dependency of skills |
| Volunteer dynamics within project | Different disciplines of volunteers in project  Number of volunteers in the project  Social support from other volunteers in country  Planned travel to destination as a group |
| **Volunteer Personal Variables** | |
| Choices made/behaviour | Desire to become culturally sensitive  Wanting to work outside of competency  Willingness to work in dangerous situations  Use of stress reduction strategies  Understanding of local context  Communication with friends/home  Feeling like a foreigner  Being realistic about achievements  Engagement with project  Willingness to learn language  Perception of placement as negative or positive experience |
| Motivations for international placement | Professional/career motivations  Personal  Cultural  Recognition from peers  Desire to help other |
| Differences between volunteers | Level of advanced preparation  Age  Locum posts before or after  Have individuals volunteered before?  Stage in professional career  Level of experience  Use of professional leave |
| **Mechanisms through which outcomes happen** | |
| Opportunities for reflection | Critical reflection  Set reflection tasks  Debrief  Self-reflection when choosing a placement  Time for post-placement reflection |
| Opportunities for clinical exposure | To experience complex situations and procedures  To be thrown out of professional comfort zone  To experience a different healthcare environment  To experience a measure to compare UK and NHS to  To experience unusual networks and hierachies  To work with higher severity of illness  To work with limited resources  To work with many illnesses: spread and volume |
| Opportunities for culturally different exposure | Risk exposure  To engage with people from culturally diverse backgrounds  To experience another culture  To experience being a foreigner  To experience challenging situations |
| Opportunities for skill development | To test coping mechanisms  To use own approaches to care  For creativity and innovation  For hands on work  For student/volunteer-centred approach to learning  To convert knowledge to know how  To develop communication skills  To challenge communication skills  To practice clinical skills  To practice speaking in another language  To put theory into practice |
| Opportunities for research skill development | To research unusual areas  To undertake collaborative research  To conduct research mutually |
| Opportunities for leadership | To be included and opinions valued  For teaching  To lead and have responsibility  To use risk management skills |
| Opportunities for atypical learning experiences | To learn about self  Mutual learning |

6: List of Core outcomes after Delphi Study: percentage of consensus, positive (include)/negative (exclusive) and the overall rank in terms of stakeholder agreement

## 7: Descriptive Statistics for Each Statement in the Delphi across the three rounds

KEY

Low- Number of stakeholders who disagreed with this statement

Med- Number of participants who gave a medium score

High- Number of participant who agreed with this statement

IK- Number who reported having insufficient knowledge

Min- Minumum score recorded

Max- Maximum score recorded

IQR 25-25% Interquartile Range

IQR75- 75% Interquartile Range

SA- number of participants who strongly agreed

SD- number of Participants who strongly disagreed

|  | **Round 1 (n=58)** | | | | | | | | | | | **Round 2 (n=49)** | | | | | | | | | **Round 3** | | | | | | | | |
| --- | --- | --- | --- | --- | --- | --- | --- | --- | --- | --- | --- | --- | --- | --- | --- | --- | --- | --- | --- | --- | --- | --- | --- | --- | --- | --- | --- | --- | --- |
|  | **Low** | **Med** | **High** | **IK** | **Median** | **Min** | **Max** | **IQR 25** | **IQR75** | **SA** | **SD** | **Low** | **Med** | **High** | **IK** | **median** | **min** | **max** | **25 IQR** | **75IQR** | **Low** | **Med** | **High** | **IK** | **Median** | **Min** | **Max** | **IQR25** | **IQR75** |
| INCREASED AWARENESS OF/KNOWLEDGE ABOUT CULTURAL DIFFERENCES AND SIMILARITIES (e.g., understanding key issues within a culture, culturally acceptable behaviour and cultures of UK immigrants, learning about, accepting and changing assumptions about other cultures) | 0 | 0 | 58 | 0 | 7 | 5 | 7 | 6 | 7 | 36 | 0 |  |  |  |  |  |  |  |  |  |  |  |  |  |  |  |  |  |  |
| INCREASED AWARENESS OF/KNOWLEDGE ABOUT THE CULTURAL ASPECTS OF HEALTH (e.g., greater understanding of health promotion, how culture affects daily life and professional work, cultural differences in health, the effects of politics on health, sustainable healthcare) | 0 | 0 | 58 | 0 | 7 | 5 | 7 | 5 | 7 | 30 | 0 |  |  |  |  |  |  |  |  |  |  |  |  |  |  |  |  |  |  |
| INCREASED AWARENESS OF/KNOWLEDGE ABOUT GLOBAL ISSUES (e.g., re-evaluating world issues, shared purpose) | 3 | 4 | 51 | 0 | 6 | 2 | 7 | 5 | 7 | 18 | 0 |  |  |  |  |  |  |  |  |  |  |  |  |  |  |  |  |  |  |
| INCREASED AWARENESS OF/KNOWLEDGE ABOUT CULTURE IN PRACTICAL ASSESSMENTS (e.g., the importance of collecting relevant cultural information about peopleâ€™s presenting health problems and learning how to conduct cultural assessments and culturally based physical assessments) | 1 | 3 | 54 | 0 | 6 | 1 | 7 | 5 | 7 | 21 | 1 |  |  |  |  |  |  |  |  |  |  |  |  |  |  |  |  |  |  |
| DEEPER ENGAGEMENT WITH ISSUES OF EQUALITY AND DIVERSITY | 3 | 7 | 48 | 0 | 6 | 3 | 7 | 5 | 6 | 13 | 0 |  |  |  |  |  |  |  |  |  |  |  |  |  |  |  |  |  |  |
| REINFORCED ETHNIC AND CULTURAL IDENTITY (e.g., understanding of own ethic and cultural identity) | 17 | 14 | 26 | 1 | 4 | 1 | 7 | 3 | 6 | 4 | 1 | 18 | 18 | 12 | 1 | 4 | 1 | 7 | 3 | 4.8 | 17 | 13 | 15 | 0 | 4 | 1 | 7 | 2.5 | 5 |
| INCREASED RESPECT FOR OTHER CULTURES | 3 | 3 | 52 | 0 | 6 | 3 | 7 | 5 | 7 | 21 | 0 |  |  |  |  |  |  |  |  |  |  |  |  |  |  |  |  |  |  |
| INCREASED CULTURAL SENSITIVITY (e.g., sensitivity to reasoning behind cultural differences, feelings of minority and language barriers) | 2 | 3 | 53 | 0 | 6 | 2 | 7 | 6 | 7 | 21 | 0 |  |  |  |  |  |  |  |  |  |  |  |  |  |  |  |  |  |  |
| UNDERSTANDING THAT SPEED AND LANGUAGE COMPETENCY AFFECT COMMUNICATION (e.g., awareness of how speed affects comprehension, understanding language differences and checking recipient comprehension, ability to use an interpreter) | 2 | 6 | 50 | 0 | 6 | 1 | 7 | 5 | 7 | 16 | 1 |  |  |  |  |  |  |  |  |  |  |  |  |  |  |  |  |  |  |
| UNDERSTANDING THAT WORDS AND BEHAVIOURS CAN HAVE DIFFERENT MEANINGS (e.g., understanding how words are perceived by others, understanding how to speak and behave so as not offend people) | 1 | 4 | 53 | 0 | 6 | 3 | 7 | 4 | 7 | 21 | 0 |  |  |  |  |  |  |  |  |  |  |  |  |  |  |  |  |  |  |
| INCREASED AWARENESS OF/KNOWLEDGE ABOUT HOW CONTEXT AFFECTS COMMUNICATION (e.g., effectively conveying ideas in a contextually appropriate way) | 4 | 5 | 49 | 0 | 6 | 2 | 7 | 5 | 6 | 11 | 0 |  |  |  |  |  |  |  |  |  |  |  |  |  |  |  |  |  |  |
| ABILITY TO OVERCOME COMMUNICATION CHALLENGES (e.g., ability to communicate effectively in high pressure situations, engage in challenging conversations and liaise between groups) | 3 | 7 | 48 | 0 | 6 | 2 | 7 | 5 | 7 | 15 | 0 |  |  |  |  |  |  |  |  |  |  |  |  |  |  |  |  |  |  |
| ABILITY TO ENGAGE SENIOR PEOPLE | 8 | 9 | 40 | 1 | 5 | 1 | 7 | 4 | 6 | 9 | 2 |  |  |  |  |  |  |  |  |  |  |  |  |  |  |  |  |  |  |
| ABILITY TO COMMUNICATE NON-VERBALLY | 5 | 9 | 44 | 0 | 5 | 2 | 7 | 4.75 | 7 | 15 | 0 |  |  |  |  |  |  |  |  |  |  |  |  |  |  |  |  |  |  |
| ABILITY TO LISTEN | 6 | 13 | 39 | 0 | 6 | 1 | 7 | 4 | 6.25 | 14 | 1 | 12 | 3 | 34 | 0 | 5 | 1 | 7 | 3.5 | 6 | 15 | 3 | 27 | 0 | 5 | 1 | 7 | 3 | 5.5 |
| ABILITY TO VERBALISE KNOWLEDGE (e.g., ability to verbalise core concepts and deep knowledge, ability to explain complex ideas to others) | 7 | 9 | 42 | 0 | 5.5 | 1 | 7 | 4 | 6 | 9 | 1 |  |  |  |  |  |  |  |  |  |  |  |  |  |  |  |  |  |  |
| ABILITY TO ESTABLISH COMMUNICATION SYSTEMS (e.g., formal and informal) | 4 | 14 | 40 | 0 | 5 | 2 | 7 | 4 | 6 | 13 | 0 | 6 | 6 | 37 | 0 | 5 | 1 | 7 | 4.5 | 6 |  |  |  |  |  |  |  |  |  |
| INCREASED AWARENESS OF/KNOWLEDGE ABOUT CONDITIONS AND PROCEDURES RARELY ENCOUNTERED IN THE UK (e.g., greater understanding of procedures not used in the UK, unfamiliar equipment and delayed presentations, better management of conditions that are not common in the UK) | 3 | 4 | 51 | 0 | 6 | 2 | 7 | 5 | 7 | 28 | 0 |  |  |  |  |  |  |  |  |  |  |  |  |  |  |  |  |  |  |
| INCREASED AWARENESS OF/KNOWLEDGE ABOUT THE IMPORTANCE OF ASSESSING HEALTHCARE ON AN INDIVIDUAL BASIS (i.e. the uniqueness of each patient) | 8 | 16 | 34 | 0 | 5 | 2 | 7 | 4 | 6 | 12 | 0 | 12 | 8 | 29 | 0 | 5 | 2 | 7 | 3.5 | 6 | 11 | 7 | 27 | 0 | 5 | 1 | 7 | 3.5 | 5 |
| INCREASED AWARENESS OF/KNOWLEDGE ABOUT THE IMPORTANCE OF COMMUNITY PARTICIPATION IN HEALTH (e.g., understanding the community and social influences on health, the role of the community in health, public health and the importance of community work) | 4 | 4 | 50 | 0 | 6 | 2 | 7 | 5 | 7 | 23 | 0 |  |  |  |  |  |  |  |  |  |  |  |  |  |  |  |  |  |  |
| INCREASED UNDERSTANDING OF BASIC SKILLS AND IDEAS (i.e., back to basics, e.g., basic observations using eyes, less reliance on lab tests and technology, basic clinical skills and science) | 3 | 3 | 52 | 0 | 6 | 3 | 7 | 4 | 7 | 24 | 0 |  |  |  |  |  |  |  |  |  |  |  |  |  |  |  |  |  |  |
| INCREASED CLINICAL KNOWLEDGE IN RELATION TO OTHER PROFESSIONS (e.g., doctors understanding nurses and vice versa, multi-disciplinary awareness) | 6 | 13 | 39 | 0 | 6 | 1 | 7 | 4 | 7 | 15 | 1 | 9 | 3 | 37 | 0 | 6 | 2 | 7 | 4.5 | 6.5 |  |  |  |  |  |  |  |  |  |
| INCREASED AWARENESS OF/KNOWLEDGE ABOUT THE POSITIVE IMPACT OF CLINICAL POLICIES AND GOVERNANCE (e.g., understanding the benefits of a comprehensive checklist) | 7 | 11 | 40 | 0 | 6 | 1 | 7 | 4 | 6 | 13 | 1 | 6 | 5 | 38 | 0 | 6 | 2 | 7 | 5 | 6 |  |  |  |  |  |  |  |  |  |
| INCREASED AWARENESS OF/KNOWLEDGE ABOUT TROPICAL DISEASES | 4 | 3 | 51 | 0 | 6 | 2 | 7 | 5 | 7 | 20 | 2 |  |  |  |  |  |  |  |  |  |  |  |  |  |  |  |  |  |  |
| INCREASED AWARENESS OF/KNOWLEDGE ABOUT APPROPRIATE CLINICAL BEHAVIOUR (e.g., knowing when to stop and when to move forward, when to ask for help and different populations needs) | 5 | 5 | 47 | 1 | 5 | 1 | 7 | 5 | 7 | 16 | 2 |  |  |  |  |  |  |  |  |  |  |  |  |  |  |  |  |  |  |
| ABILITY TO APPLY EVIDENCE BASED PRACTICE (e.g., understanding its importance (sometimes through being unable to apply it overseas), understanding how to apply it innovatively with limited resources) | 13 | 11 | 34 | 0 | 5.5 | 1 | 7 | 4 | 6 | 13 | 1 | 16 | 5 | 28 | 0 | 5 | 1 | 7 | 3 | 6 | 11 | 6 | 28 | 0 | 5 | 1 | 7 | 3.5 | 5 |
| ABILITY TO OBSERVE AND EXAMINE PATIENTS (e.g., increased intuitive knowledge of clinical signs and clinical judgement ability to make diagnosis without investigations) | 11 | 4 | 43 | 0 | 6 | 1 | 7 | 4 | 7 | 15 | 1 |  |  |  |  |  |  |  |  |  |  |  |  |  |  |  |  |  |  |
| ABILITY TO BE INNOVATIVE WITH CLINICAL SKILLS (e.g., use of innovative techniques, finding new ways to approach a condition, new ways of working) | 7 | 3 | 48 | 0 | 6 | 2 | 7 | 5 | 7 | 23 | 0 |  |  |  |  |  |  |  |  |  |  |  |  |  |  |  |  |  |  |
| ABILITY TO USE A BROADER RANGE OF CLINICAL SKILLS (e.g., enhancing existing skills and acquiring new clinical skills, greater all round competence) | 3 | 5 | 50 | 0 | 6 | 2 | 7 | 5 | 7 | 17 | 0 |  |  |  |  |  |  |  |  |  |  |  |  |  |  |  |  |  |  |
| ABILITY TO APPLY CLINICAL SKILLS TO ANOTHER CONTEXT (e.g., a more challenging environment or a low resource setting) | 2 | 2 | 54 | 0 | 6 | 2 | 7 | 5 | 7 | 25 | 0 |  |  |  |  |  |  |  |  |  |  |  |  |  |  |  |  |  |  |
| ABILITY TO MAKE INDEPENDENT CLINICAL DECISIONS (e.g., ability to make an urgent decision in an emergency, dealing with uncertain outcomes, evaluating risks to patients and self) | 4 | 7 | 47 | 0 | 6 | 3 | 7 | 5 | 7 | 2 | 0 |  |  |  |  |  |  |  |  |  |  |  |  |  |  |  |  |  |  |
| ABILITY TO WORK IN A PROFESSIONALLY COMPETENT WAY (e.g., having wider view of profession, intellectual development, reminder of professional responsibilities, stronger work ethic) | 6 | 9 | 43 | 0 | 5 | 2 | 7 | 4 | 6 | 12 | 0 |  |  |  |  |  |  |  |  |  |  |  |  |  |  |  |  |  |  |
| INCREASED AWARENESS OF/KNOWLEDGE ABOUT THE IMPORTANCE OF MUTUAL LEARNING AND RESPECT (i.e., greater understanding of reciprocal learning) | 4 | 3 | 51 | 0 | 6 | 2 | 7 | 5 | 7 | 16 | 0 |  |  |  |  |  |  |  |  |  |  |  |  |  |  |  |  |  |  |
| INCREASED UNDERSTANDING OF HOW TO BE A GOOD TEACHER (e.g., allowing students to learn from mistakes, ability to suggest and acknowledge improvements in teaching, understanding how communication affects learning, how to target training most effectively and the importance of experiential learning) | 7 | 8 | 42 | 1 | 6 | 2 | 7 | 4 | 7 | 16 | 0 |  |  |  |  |  |  |  |  |  |  |  |  |  |  |  |  |  |  |
| INCREASED AWARENESS OF/KNOWLEDGE ABOUT THE NEED FOR AND IMPORTANCE OF TRAINING (i.e., understanding how important effective training is in) | 6 | 3 | 49 | 0 | 6 | 2 | 7 | 5 | 7 | 16 | 0 |  |  |  |  |  |  |  |  |  |  |  |  |  |  |  |  |  |  |
| IMPROVEMENT IN TEACHING SKILLS (e.g., learning new techniques, greater training delivery skills, lecturing skills and small group teaching skills) | 4 | 5 | 49 | 0 | 6 | 2 | 7 | 5 | 6 | 13 | 0 |  |  |  |  |  |  |  |  |  |  |  |  |  |  |  |  |  |  |
| ABILITY TO BE ADAPTABLE AND INNOVATIVE IN TEACHING (e.g., ability to transfer skills and knowledge to the most influential people or to another context, recognising different learning styles, being adaptable in assessment) | 4 | 0 | 54 | 0 | 6 | 2 | 7 | 5 | 6.25 | 14 | 0 |  |  |  |  |  |  |  |  |  |  |  |  |  |  |  |  |  |  |
| CONFIDENCE IN TEACHING ABILITY (e.g., being more comfortable around others, confidence public speaking, confidence in transferring knowledge) | 4 | 2 | 52 | 0 | 6 | 2 | 7 | 5 | 6 | 9 | 0 |  |  |  |  |  |  |  |  |  |  |  |  |  |  |  |  |  |  |
| INCREASED AWARENESS OF/KNOWLEDGE ABOUT THE IMPORTANCE OF CONSCIOUSLY MAKING AN EFFORT TO GET ON WITH COLLEAGUES (e.g., learning colleagueâ€™s names) | 11 | 11 | 36 | 0 | 5 | 1 | 7 | 4 | 6 | 10 | 1 | 10 | 4 | 35 | 0 | 5 | 2 | 7 | 4 | 6 |  |  |  |  |  |  |  |  |  |
| UNDERSTANDING OWN POTENTIAL TO EMPOWER PEOPLE   *You have now completed 25%* | 3 | 8 | 47 | 0 | 6 | 2 | 7 | 5 | 6 | 13 | 1 |  |  |  |  |  |  |  |  |  |  |  |  |  |  |  |  |  |  |
| INCREASED AWARENESS OF/KNOWLEDGE ABOUT THE IMPORTANCE OF TRUST BETWEEN COLLEAGUES WITHIN HEALTHCARE SYSTEMS | 5 | 11 | 42 | 0 | 5 | 2 | 7 | 4 | 6 | 13 | 0 |  |  |  |  |  |  |  |  |  |  |  |  |  |  |  |  |  |  |
| ABILITY TO GET THE MOST OUT OF PEOPLE (e.g., encouraging people to work together, recognise their own strengths and to take possession of their own work/projects, ability to assess the capability of others) | 8 | 6 | 44 | 0 | 5 | 2 | 7 | 4.75 | 6 | 11 | 0 |  |  |  |  |  |  |  |  |  |  |  |  |  |  |  |  |  |  |
| ABILITY TO BE ADAPTABLE IN LEADING (e.g., able to lead in complex novel situations, ability to compromise not dictate) | 3 | 4 | 51 | 0 | 6 | 2 | 7 | 5 | 7 | 17 | 0 |  |  |  |  |  |  |  |  |  |  |  |  |  |  |  |  |  |  |
| ABILITY TO MANAGE HEALTHCARE ENVIRONMENTS (e.g., ability to manage wards and staff) | 10 | 7 | 41 | 0 | 5 | 2 | 7 | 4 | 6 | 11 | 0 |  |  |  |  |  |  |  |  |  |  |  |  |  |  |  |  |  |  |
| ABILITY TO MANAGE PEOPLE (e.g., able to allocate tasks and co-ordinate people, to deal with people with differing objectives, to negotiate with multiple stakeholders, to manage difficult people) | 5 | 9 | 44 | 0 | 5 | 1 | 7 | 4.75 | 6 | 9 | 1 |  |  |  |  |  |  |  |  |  |  |  |  |  |  |  |  |  |  |
| ABILITY TO WORK WITHIN A SYSTEM WITH UNFAMILIAR POWER DYNAMICS | 4 | 3 | 51 | 0 | 6 | 2 | 7 | 5 | 7 | 19 | 0 |  |  |  |  |  |  |  |  |  |  |  |  |  |  |  |  |  |  |
| ABILITY TO CO-OPERATE (e.g., willingness to see another point of view) | 7 | 5 | 46 | 0 | 6 | 2 | 7 | 5 | 7 | 19 | 0 |  |  |  |  |  |  |  |  |  |  |  |  |  |  |  |  |  |  |
| ABILITY TO WORK AS PART OF A TEAM (e.g., understanding team group norms, perception of roles within the group, managing personal objectives within a group) | 5 | 6 | 47 | 0 | 6 | 1 | 7 | 5 | 7 | 16 | 2 |  |  |  |  |  |  |  |  |  |  |  |  |  |  |  |  |  |  |
| ABILITY TO DEVELOP FRIENDSHIPS (e.g., relationship formation skills, developing new friendships) | 10 | 4 | 44 | 0 | 5 | 1 | 7 | 4.75 | 7 | 16 | 2 |  |  |  |  |  |  |  |  |  |  |  |  |  |  |  |  |  |  |
| ABILITY TO BUILD A GLOBAL NETWORK | 8 | 3 | 46 | 0 | 6 | 1 | 7 | 5 | 7 | 18 | 2 |  |  |  |  |  |  |  |  |  |  |  |  |  |  |  |  |  |  |
| ABILITY TO DISSEMINATION BEST PRACTICE GLOBALLY | 6 | 5 | 46 | 0 | 5 | 1 | 7 | 5 | 6 | 12 | 1 |  |  |  |  |  |  |  |  |  |  |  |  |  |  |  |  |  |  |
| ABILITY TO GIVE AND ACCEPT PRAISE | 10 | 16 | 31 | 0 | 5 | 1 | 7 | 4 | 6 | 11 | 2 | 13 | 10 | 26 | 0 | 5 | 1 | 7 | 3 | 5 | 15 | 6 | 24 | 0 | 5 | 1 | 7 | 3 | 5 |
| ABILITY TO ADAPT SOCIAL NORMS TO MEET NEEDS OF ANOTHER CULTURE (e.g., change behaviours to fit into another culture, being aware of own social norms and adapting them) | 3 | 4 | 51 | 0 | 6 | 1 | 7 | 5 | 7 | 16 | 1 |  |  |  |  |  |  |  |  |  |  |  |  |  |  |  |  |  |  |
| ABILITY TO ENCOURAGE OTHERS TO TAKE RESPONSIBILITY FOR OWN HEALTH | 14 | 16 | 27 | 1 | 4 | 1 | 7 | 3.5 | 6 | 9 | 1 | 13 | 12 | 23 | 1 | 4 | 2 | 7 | 3 | 6 | 13 | 12 | 20 | 0 | 4 | 2 | 7 | 3 | 5 |
| ABILITY TO EXCHANGE IDEAS WITH THOSE FROM ANOTHER CULTURE | 4 | 3 | 51 | 0 | 6 | 2 | 7 | 5 | 7 | 20 | 0 |  |  |  |  |  |  |  |  |  |  |  |  |  |  |  |  |  |  |
| ACT AS A ROLE MODEL (e.g., lead by example) | 7 | 8 | 43 | 0 | 5.5 | 2 | 7 | 4 | 6 | 13 | 0 |  |  |  |  |  |  |  |  |  |  |  |  |  |  |  |  |  |  |
| UNDERSTANDING THAT CHANGING BEHAVIOUR IS COMPLEX (e.g., understanding how to make small changes and not to force your perspective onto others,) | 2 | 6 | 50 | 0 | 6 | 2 | 7 | 5 | 7 | 19 | 0 |  |  |  |  |  |  |  |  |  |  |  |  |  |  |  |  |  |  |
| INCREASED ABILITY TO CHANGE BEHAVIOUR IN COLLEAGUES OR PATIENTS (e.g., ability to implement behaviour change and to assess the impact of healthcare systems) | 8 | 13 | 37 | 0 | 5 | 1 | 7 | 4 | 6 | 5 | 1 | 6 | 9 | 34 | 0 | 5 | 1 | 7 | 4 | 5.5 | 9 | 3 | 33 | 0 | 5 | 1 | 7 | 4 | 5 |
| INCREASED AWARENESS OF/KNOWLEDGE ABOUT HOW OTHER HEALTHCARE SYSTEMS FUNCTION (e.g., developed insight into disparities within healthcare systems, understanding of other systems) | 0 | 4 | 54 | 0 | 6 | 4 | 7 | 4 | 6 | 17 | 0 |  |  |  |  |  |  |  |  |  |  |  |  |  |  |  |  |  |  |
| INCREASED AWARENESS OF/KNOWLEDGE ABOUT ETHICS (i.e., experiencing ethical dilemmas, understanding the importance of ethics) | 6 | 7 | 45 | 0 | 6 | 2 | 7 | 5 | 6 | 12 | 0 |  |  |  |  |  |  |  |  |  |  |  |  |  |  |  |  |  |  |
| INCREASED AWARENESS OF AND KNOWLEDGE THE FUNCTIONING OF SYSTEMS (e.g., able to identify stakeholders and change agents, understanding influencing patterns of those in power, value systems and the difficulty of questioning organisations) | 5 | 11 | 42 | 0 | 5 | 2 | 7 | 4 | 6 | 12 | 0 |  |  |  |  |  |  |  |  |  |  |  |  |  |  |  |  |  |  |
| INCREASED AWARENESS OF/KNOWLEDGE ABOUT THE COSTS OF HEALTHCARE | 7 | 10 | 41 | 0 | 5.5 | 2 | 7 | 4 | 7 | 15 | 0 |  |  |  |  |  |  |  |  |  |  |  |  |  |  |  |  |  |  |
| ABILITY TO IMPROVE SERVICE (e.g., renewed enthusiasm for service improvement) | 4 | 4 | 50 | 0 | 6 | 2 | 7 | 5 | 7 | 16 | 0 |  |  |  |  |  |  |  |  |  |  |  |  |  |  |  |  |  |  |
| ABILITY TO APPLY KNOWLEDGE ACROSS SYSTEMS (e.g., ability to apply knowledge from host system to UK and vice versa, using knowledge gained in system to improve/change another) | 1 | 4 | 53 | 0 | 6 | 2 | 7 | 5 | 7 | 16 | 0 |  |  |  |  |  |  |  |  |  |  |  |  |  |  |  |  |  |  |
| APPRECIATION OF CLINICAL GOVERNANCE PROCEDURES WITHIN NHS (e.g., waste disposal, audit, teamwork, education system, tests and investigations) | 9 | 3 | 46 | 0 | 6 | 1 | 7 | 5 | 7 | 17 | 1 |  |  |  |  |  |  |  |  |  |  |  |  |  |  |  |  |  |  |
| APPRECIATION OF FREE UNIVERSAL HEALTH (e.g., the NHS system of free healthcare for all, privilege and opportunity, the expectations that are placed on NHS by service users) | 6 | 5 | 47 | 0 | 6 | 2 | 7 | 5 | 7 | 25 | 0 |  |  |  |  |  |  |  |  |  |  |  |  |  |  |  |  |  |  |
| APPRECIATION OF HAVING THE RIGHT TOOLS AND EQUIPMENT TO BE ABLE TO DO THE JOB (i.e., resources: technical equipment, disposal equipment, cleaning products and protective equipment) | 5 | 5 | 48 | 0 | 6 | 2 | 7 | 5 | 7 | 19 | 0 |  |  |  |  |  |  |  |  |  |  |  |  |  |  |  |  |  |  |
| APPRECIATION OF EXCELLENT HUMAN RESOURCE IN THE NHS (e.g., multidisciplinary teams, HR structures, appreciation of own profession, understanding hierarchy and the importance of each person within it) | 5 | 5 | 48 | 0 | 6 | 2 | 7 | 5 | 7 | 19 | 0 |  |  |  |  |  |  |  |  |  |  |  |  |  |  |  |  |  |  |
| IMPROVED SITUATIONAL AWARENESS (i.e., understanding your environment so you can understand what to do) | 6 | 5 | 47 | 0 | 6 | 2 | 7 | 5 | 7 | 19 | 0 |  |  |  |  |  |  |  |  |  |  |  |  |  |  |  |  |  |  |
| INCREASED SELF-AWARENESS (e.g., understanding own skills and limitations, how to challenge own beliefs and importance of reflecting on own situation) | 3 | 4 | 51 | 0 | 6 | 2 | 7 | 5 | 7 | 23 | 0 |  |  |  |  |  |  |  |  |  |  |  |  |  |  |  |  |  |  |
| ABILITY TO SPEAK THE HOST LANGUAGE | 13 | 13 | 30 | 2 | 5 | 1 | 7 | 4 | 5 | 3 | 2 | 11 | 12 | 25 | 1 | 5 | 1 | 6 | 4 | 5 | 9 | 10 | 26 | 0 | 5 | 1 | 7 | 4 | 5 |
| ABILITY TO ACCEPT AND UNDERSTAND FAILURE (e.g., to continue with something that did not have desired outcome at first, learning to accept failure, thinking differently about failure, persistence) | 10 | 7 | 41 | 0 | 5 | 2 | 7 | 4 | 6 | 11 | 0 |  |  |  |  |  |  |  |  |  |  |  |  |  |  |  |  |  |  |
| ABILITY TO COPE (e.g., improved coping strategies, ability to deal with lack of structure, knock backs and stress, being unfazed by things and taking things in stride, new approach to guilt for patients problems) | 2 | 2 | 54 | 0 | 6 | 2 | 7 | 5 | 7 | 21 | 0 |  |  |  |  |  |  |  |  |  |  |  |  |  |  |  |  |  |  |
| ABILITY TO CHALLENGE BREACHES OF PRIVACY AND CONFIDENTIALITY (e.g., ability to stand up for patients/peoples rights if they are jeopardised, increased awareness of human rights, ability to respect regulatory standards of home and overseas regulatory bodies) | 10 | 9 | 38 | 1 | 5 | 1 | 7 | 4 | 6 | 9 | 1 | 11 | 6 | 31 | 1 | 5 | 2 | 7 | 4 | 5.8 | 8 | 6 | 30 | 1 | 5 | 2 | 7 | 4 | 6 |
| ABILITY TO MANAGE SELF (e.g., own expectations, self-reliance, self-management, self-assurance, reflexivity) | 6 | 8 | 44 | 0 | 6 | 1 | 7 | 4.75 | 7 | 18 | 1 |  |  |  |  |  |  |  |  |  |  |  |  |  |  |  |  |  |  |
| AN UPPER HAND WHEN COMPETING FOR CAREERS | 20 | 8 | 28 | 2 | 4.5 | 1 | 7 | 3 | 6 | 8 | 5 | 16 | 11 | 20 | 2 | 4 | 1 | 7 | 3 | 6 | 14 | 13 | 18 | 0 | 4 | 1 | 7 | 3 | 5 |
| INCREASED JOB SATISFACTION (e.g., increased motivation and morale within profession, renewed passion for work, sense of reward) | 3 | 8 | 46 | 1 | 6 | 2 | 7 | 5 | 7 | 15 | 0 |  |  |  |  |  |  |  |  |  |  |  |  |  |  |  |  |  |  |
| INFLUENCES CAREER PATHWAY (i.e., affects specialism choice, exploration of potential career pathways, pursuing careers in primary care, family practice, public service, sub-specialism in global health, teaching) | 9 | 6 | 42 | 1 | 6 | 1 | 7 | 4 | 6 | 12 | 2 |  |  |  |  |  |  |  |  |  |  |  |  |  |  |  |  |  |  |
| SPIRITUAL DEVELOPMENT | 14 | 15 | 27 | 2 | 4 | 1 | 7 | 3.25 | 6 | 7 | 5 | 13 | 18 | 17 | 1 | 4 | 1 | 7 | 3 | 5 | 15 | 18 | 11 | 1 | 4 | 1 | 6 | 2.25 | 4.75 |
| REFRESHMENT AND REINVIGORATION (e.g., chance to take time away to become refreshed and feel reinvigorated to work upon return) | 8 | 8 | 42 | 0 | 6 | 2 | 7 | 4 | 6 | 13 | 0 |  |  |  |  |  |  |  |  |  |  |  |  |  |  |  |  |  |  |
| PERSONAL SATISFACTION (e.g., personal achievements and challenges, new experiences, experiencing a different lifestyle, a holiday, appreciation of own life, personal fulfilment) | 4 | 7 | 47 | 0 | 6 | 2 | 7 | 5 | 7 | 18 | 0 |  |  |  |  |  |  |  |  |  |  |  |  |  |  |  |  |  |  |
| DEVELOPMENT OF A NEW PERSPECTIVE (e.g., revising assumptions, seeing things differently, changed world views and outlook, look at everything in a new light, openness to new experiences, put things into perspective) | 2 | 3 | 53 | 0 | 6 | 2 | 7 | 5 | 7 | 20 | 0 |  |  |  |  |  |  |  |  |  |  |  |  |  |  |  |  |  |  |
| ESCAPISM (e.g., freedom from bureaucracy, space outside of regular routine to clarify objectives, escape from agendas and workload, a chance to take time out of training and practice) | 17 | 10 | 31 | 0 | 5 | 1 | 7 | 3 | 6 | 10 | 7 | 14 | 7 | 27 | 1 | 5 | 1 | 7 | 3 | 6 | 11 | 4 | 30 | 0 | 5 | 1 | 7 | 3.5 | 5 |
| CHANGED PERCEPTION OF OTHERNESS (e.g., understanding importance of being a friendly stranger in UK, feeling like a foreigner) | 6 | 7 | 45 | 0 | 5.5 | 1 | 7 | 5 | 7 | 15 | 2 |  |  |  |  |  |  |  |  |  |  |  |  |  |  |  |  |  |  |
| APPRECIATION OF THE IMPORTANCE OF CARE AND COMPASSION (e.g., ability to compare compassion in both systems, empathy and fairness) | 6 | 6 | 46 | 0 | 6 | 2 | 7 | 5 | 7 | 17 | 0 |  |  |  |  |  |  |  |  |  |  |  |  |  |  |  |  |  |  |
| IMPROVED EMOTIONAL INTELLIGENCE (e.g., changed engagement with self, knowledge and world) | 4 | 6 | 48 | 0 | 6 | 2 | 7 | 5 | 6 | 11 | 0 |  |  |  |  |  |  |  |  |  |  |  |  |  |  |  |  |  |  |
| IMPROVED FLEXIBILITY AND ADAPTABILITY (e.g., acceptance of other ways of working, adaptation to responsibility, being able to adapt more easily to unfamiliar situations, able to cope more easily with change, gaining a wider perspective, understanding the flexibility of roles) | 2 | 3 | 53 | 0 | 6 | 2 | 7 | 5 | 7 | 17 | 0 |  |  |  |  |  |  |  |  |  |  |  |  |  |  |  |  |  |  |
| IMPROVED CONFIDENCE (e.g., in caring for clients from another culture, in quality improvement methods, to take bolder steps, to address challenging situations, self-confidence, confidence in professional ability,) | 2 | 4 | 52 | 0 | 6 | 2 | 7 | 5 | 7 | 20 | 0 |  |  |  |  |  |  |  |  |  |  |  |  |  |  |  |  |  |  |
| PATIENCE AND TOLERANCE (e.g., accepting and working at other peoples pace, more tolerant) | 4 | 3 | 51 | 0 | 6 | 1 | 7 | 5 | 6 | 12 | 1 |  |  |  |  |  |  |  |  |  |  |  |  |  |  |  |  |  |  |
| PROACTIVITY (e.g., thinking on feet, using initiative, efficiency, get on with things rather than look for someone to blame) | 2 | 5 | 51 | 0 | 6 | 2 | 7 | 5 | 7 | 18 | 0 |  |  |  |  |  |  |  |  |  |  |  |  |  |  |  |  |  |  |
| CHANGED JUDGEMENT (e.g., non-judgemental attitude, changed self-judgement) | 6 | 8 | 44 | 0 | 5 | 2 | 7 | 4.75 | 6 | 10 | 0 |  |  |  |  |  |  |  |  |  |  |  |  |  |  |  |  |  |  |
| CAN-DO ATTITUDE | 5 | 13 | 40 | 0 | 5.5 | 2 | 7 | 4 | 6 | 13 | 0 | 3 | 6 | 39 | 1 | 6 | 2 | 7 | 5 | 6 |  |  |  |  |  |  |  |  |  |
| HUMILITY (including professional humility) | 8 | 9 | 41 | 0 | 5 | 1 | 7 | 4 | 6 | 13 | 1 |  |  |  |  |  |  |  |  |  |  |  |  |  |  |  |  |  |  |
| DIPLOMACY | 6 | 8 | 44 | 0 | 6 | 2 | 7 | 4.75 | 7 | 19 | 0 |  |  |  |  |  |  |  |  |  |  |  |  |  |  |  |  |  |  |
| INTEGRITY | 5 | 8 | 45 | 0 | 6 | 1 | 7 | 5 | 7 | 17 | 1 |  |  |  |  |  |  |  |  |  |  |  |  |  |  |  |  |  |  |
| INDEPENDENCE (e.g., lone working) | 5 | 8 | 45 | 0 | 6 | 1 | 7 | 5 | 7 | 17 | 1 |  |  |  |  |  |  |  |  |  |  |  |  |  |  |  |  |  |  |
| CONFIDENCE TO WORK IN OTHER LOCATIONS (e.g., confidence to move to another city/country, working with UK multicultural/ underserved populations) | 2 | 4 | 51 | 1 | 6 | 2 | 7 | 5 | 7 | 19 | 0 |  |  |  |  |  |  |  |  |  |  |  |  |  |  |  |  |  |  |
| ABILITY TO BE INNOVATE WHEN OVERCOMING CHALLENGES (i.e., finding unique ways of overcoming cultural and language challenges) | 2 | 3 | 53 | 0 | 6 | 2 | 7 | 5 | 7 | 21 | 0 |  |  |  |  |  |  |  |  |  |  |  |  |  |  |  |  |  |  |
| ABILITY TO WORK WITH RESOURCES AVAILABLE IN SPECIFIC CONTEXTS (i.e., understanding the reasons behind lack of resources) | 4 | 3 | 51 | 0 | 6 | 2 | 7 | 5 | 7 | 22 | 0 |  |  |  |  |  |  |  |  |  |  |  |  |  |  |  |  |  |  |
| ABILITY TO WORK WITH LIMITED RESOURCES (e.g., being more resourceful, ability to target resources, ability to find solutions despite limited resources, making use of everything available, ability to work without reliance on technology, manage in a low resource setting) | 2 | 1 | 55 | 0 | 6 | 2 | 7 | 6 | 7 | 26 | 0 |  |  |  |  |  |  |  |  |  |  |  |  |  |  |  |  |  |  |
| ABILITY TO PLAN AND ORGANISE (e.g., ability to set direction, improved audit skills) | 7 | 6 | 45 | 0 | 6 | 2 | 7 | 5 | 7 | 18 | 0 |  |  |  |  |  |  |  |  |  |  |  |  |  |  |  |  |  |  |
| ABILITY TO DEAL WITH THE UNEXPECTED | 4 | 5 | 49 | 0 | 6 | 2 | 7 | 5 | 7 | 20 | 0 |  |  |  |  |  |  |  |  |  |  |  |  |  |  |  |  |  |  |
| ABILITY TO IDENTIFY AND ANTICIPATE POTENTIAL PROBLEMS (e.g., identify problems when setting up a new project) | 5 | 5 | 48 | 0 | 6 | 2 | 7 | 5 | 6 | 10 | 0 |  |  |  |  |  |  |  |  |  |  |  |  |  |  |  |  |  |  |
| ABILITY TO WORK TOWARDS SOLUTIONS (e.g., solution focused approach) | 4 | 3 | 51 | 0 | 6 | 2 | 7 | 5 | 6.25 | 14 | 0 |  |  |  |  |  |  |  |  |  |  |  |  |  |  |  |  |  |  |
| ABILITY TO FIND FACTS TO SOLVE PROBLEMS | 6 | 8 | 44 | 0 | 6 | 2 | 7 | 4.75 | 6 | 11 | 0 |  |  |  |  |  |  |  |  |  |  |  |  |  |  |  |  |  |  |
| ABILITY TO MAKE DECISIONS (e.g., understanding who the decision is for, taking action on decision, making judgements | 5 | 8 | 45 | 0 | 6 | 2 | 7 | 5 | 7 | 15 | 0 |  |  |  |  |  |  |  |  |  |  |  |  |  |  |  |  |  |  |
| ABILITY TO MANAGE RISK (e.g., manage risk in advance, evaluation of environment, understanding the clinical importance of risk management and the wider implication of poorly managed risk) | 7 | 6 | 45 | 0 | 6 | 2 | 7 | 5 | 7 | 15 | 0 |  |  |  |  |  |  |  |  |  |  |  |  |  |  |  |  |  |  |
| ABILITY TO MANAGE TIME AND PRIORITISE (e.g., ability to respond quickly in an emergency, managing immediate need vs long term need, prioritisation of limited resources) | 9 | 6 | 43 | 0 | 6 | 2 | 7 | 4 | 6 | 11 | 0 |  |  |  |  |  |  |  |  |  |  |  |  |  |  |  |  |  |  |
| ABILITY TO MANAGE PROJECTS | 8 | 13 | 37 | 0 | 5 | 1 | 7 | 4 | 6 | 12 | 1 | 5 | 3 | 41 | 0 | 5 | 1 | 7 | 5 | 6 |  |  |  |  |  |  |  |  |  |
| ABILITY TO MANAGE TRAGEDIES | 8 | 12 | 36 | 2 | 5 | 1 | 7 | 4 | 6 | 9 | 1 | 6 | 7 | 36 | 0 | 5 | 1 | 7 | 4 | 6 |  |  |  |  |  |  |  |  |  |
| ABILITY TO PROVIDE BETTER CARE (e.g., ability to integrate primary and secondary care, to provide multicultural care, to develop most effective approaches to care and taking responsibility for providing quality of care) | 7 | 5 | 46 | 0 | 6 | 2 | 7 | 5 | 6 | 13 | 0 |  |  |  |  |  |  |  |  |  |  |  |  |  |  |  |  |  |  |
| IMPROVED RESEARCH SKILLS (e.g., grant application skills, research design and implementation) | 11 | 14 | 31 | 2 | 5 | 2 | 7 | 4 | 6 | 12 | 0 | 14 | 7 | 28 | 0 | 5 | 1 | 7 | 3 | 6 | 21 | 4 | 20 | 0 | 4 | 1 | 7 | 3 | 5 |
| ABILITY TO PRESENT WORK | 12 | 10 | 36 | 0 | 5 | 1 | 7 | 4 | 6 | 12 | 1 | 11 | 6 | 32 | 0 | 5 | 2 | 7 | 4 | 6 | 13 | 2 | 30 | 0 | 5 | 1 | 7 | 3 | 5 |
| ABILITY TO WRITE REPORTS AND ACADEMIC PIECES | 15 | 15 | 28 | 0 | 4 | 1 | 7 | 3 | 5 | 8 | 3 | 12 | 8 | 29 | 0 | 5 | 1 | 7 | 3.5 | 6 | 17 | 2 | 26 | 0 | 5 | 1 | 7 | 3 | 5 |
| ABILITY TO THINK THROUGH PROBLEMS IN A LOGICAL WAY (e.g., analytical/lateral thinking) | 7 | 10 | 41 | 0 | 5 | 2 | 7 | 4 | 6 | 10 | 1 | 4 | 9 | 36 |  |  |  |  |  |  |  |  |  |  |  |  |  |  |  |
| INCREASED INTERNATIONAL REPUTATION (of UK) | 8 | 12 | 37 | 1 | 6 | 1 | 7 | 4 | 6 | 10 | 2 | 4 | 9 | 36 | 0 | 6 | 1 | 7 | 4 | 6 |  |  |  |  |  |  |  |  |  |
| REDUCTION IN NHS DROP OUTS (e.g., increased staff retention, when they volunteer and come back to NHS) | 9 | 14 | 32 | 3 | 5 | 1 | 7 | 4 | 6 | 6 | 1 | 7 | 5 | 36 | 1 | 5 | 2 | 7 | 4.25 | 6 |  |  |  |  |  |  |  |  |  |
| INCREASED WORKFORCE PRODUCTIVITY | 9 | 14 | 32 | 3 | 5 | 2 | 7 | 4 | 6 | 9 | 3 | 5 | 9 | 33 | 2 | 5 | 1 | 7 | 4 | 6 |  |  |  |  |  |  |  |  |  |
| INCREASED STAFF KNOWLEDGE AND SKILLS (e.g., increased staff knowledge of low cost healthcare, more knowledgeable staff able to cover more areas, to discover better ways of doing things and more aware of waste reduction) | 8 | 11 | 36 | 3 | 6 | 3 | 7 | 5 | 6 | 12 | 1 |  |  |  |  |  |  |  |  |  |  |  |  |  |  |  |  |  |  |
| INCREASED INTERNATIONAL REPUTATION OF NHS (e.g., greater fulfilment of social responsibility) | 1 | 7 | 49 | 1 | 5.5 | 2 | 7 | 4 | 6 | 11 | 0 |  |  |  |  |  |  |  |  |  |  |  |  |  |  |  |  |  |  |
| NHS BECOMES A MORE ATTRACTIVE EMPLOYER (e.g., an employer that offers staff the opportunity to volunteer) | 2 | 13 | 41 | 2 | 5 | 2 | 7 | 4 | 6 | 12 | 0 |  |  |  |  |  |  |  |  |  |  |  |  |  |  |  |  |  |  |
| INCREASED PATIENT SATISFACTION (e.g., staff better able to respond to UK multicultural populations, staff able to compare how systems affect patient satisfaction, have greater relationships with multicultural population, more in tune with patients and more aware of individual needs of patients). | 4 | 9 | 43 | 2 | 6 | 2 | 7 | 5 | 6 | 12 | 0 |  |  |  |  |  |  |  |  |  |  |  |  |  |  |  |  |  |  |
| MEDICAL SCHOOL MORE ATTRACTIVE TO STUDENTS (e.g., if allows students to go abroad) | 7 | 11 | 37 | 3 | 5 | 1 | 7 | 4 | 6 | 11 | 1 | 5 | 10 | 34 | 0 | 5 | 1 | 7 | 4 | 6 | 5 | 9 | 30 | 1 | 5 | 1 | 7 | 4 | 5 |
| COSTS TO BRITISH PATIENTS (e.g., staff desensitised, staff less tolerant and patient, staff bringing tropical illnesses to UK) | 29 | 6 | 23 | 0 | 3.5 | 1 | 7 | 1.75 | 5 | 8 | 14 | 20 | 11 | 18 | 0 | 4 | 1 | 7 | 2 | 5 | 19 | 11 | 15 | 0 | 4 | 1 | 7 | 2 | 5 |
| LOSS OF TRAINED STAFF (e.g., utilisation of key staff time, financial cost of losing staff, having to find cover for staff) | 32 | 5 | 21 | 0 | 3 | 1 | 7 | 2 | 5 | 6 | 12 | 26 | 11 | 12 | 0 | 3 | 1 | 7 | 3 | 4.5 | 25 | 5 | 15 | 0 | 3 | 1 | 6 | 2 | 5 |
| REDUCTION IN STAFF COMPETENCE (e.g., brain drain reversal: NHS loss of competent staff to overseas placements, staff unable to cope with paperwork on return) | 33 | 6 | 19 | 0 | 3 | 1 | 7 | 1 | 3 | 5 | 17 | 31 | 7 | 11 | 0 | 3 | 1 | 7 | 2 | 4 | 33 | 5 | 7 | 0 | 3 | 1 | 7 | 2 | 4 |
| NEGATIVE PERCEPTIONS OF NHS (e.g., NHS reputation jeopardised if a health link is badly organised) | 38 | 7 | 13 | 0 | 3 | 1 | 7 | 1 | 4 | 2 | 16 | 30 | 8 | 11 | 0 | 3 | 1 | 6 | 2 | 4 | 29 | 5 | 11 | 0 | 3 | 1 | 6 | 2 | 4.5 |
| DISTRACTED STAFF (e.g., staff going on international placements coming back disengaged with UK work and pre-occupied) | 31 | 10 | 17 | 0 | 3 | 1 | 7 | 1.75 | 5 | 2 | 14 | 29 | 9 | 11 | 0 | 3 | 1 | 6 | 2 | 4 | 29 | 8 | 8 | 0 | 3 | 1 | 6 | 2 | 4 |
| DEVELOPING REDUNDANT OR BAD SKILLS/ATTITUDES (e.g., developing non-transferable skills, bad habits, deskilling, returning with overconfidence in own ability, poorer communication skills, loss of confidence) | 39 | 6 | 13 | 0 | 3 | 1 | 7 | 1 | 4 | 2 | 15 | 37 | 4 | 8 | 0 | 3 | 1 | 7 | 2 | 3.5 |  |  |  |  |  |  |  |  |  |
| DIFFICULTY GETTING THE JOB OR TRAINING POSITION THAT YOU WANT UPON RETURN (e.g., returning to work in a locum position, not having a permanent job upon return) | 23 | 10 | 22 | 3 | 4 | 1 | 7 | 3 | 5 | 3 | 8 | 17 | 14 | 16 | 2 | 4 | 1 | 6 | 2 | 5 | 15 | 16 | 14 | 0 | 4 | 1 | 6 | 3 | 5 |
| EXPOSURE TO ETHICAL DILEMMAS (e.g., expected to work outside of competency, to do clinical work, little regulation, little supervision, too much responsibility) | 17 | 9 | 32 | 0 | 5 | 1 | 7 | 2 | 5 | 3 | 8 | 8 | 5 | 36 | 0 | 5 | 1 | 7 | 4 | 5 |  |  |  |  |  |  |  |  |  |
| NO RECOGNITION OR ACCREDITATION UPON RETURN | 18 | 6 | 33 | 1 | 5 | 1 | 7 | 2.5 | 6 | 7 | 8 | 10 | 7 | 32 | 0 | 5 | 1 | 7 | 4 | 5 | 6 | 6 | 33 | 0 | 5 | 1 | 6 | 4 | 5.5 |
| REDUCED EXPERIENCE AND EXPOSURE TO UK PROCEDURES, PROTOCOLS AND RESEARCH (e.g., NHS procedures that donâ€™t exist in host country, missing out on formal training and conferences, chronic disease management over time, health conditions that are common in UK and not in host country, NHS protocol and updates, loss of professional networks and relationships) | 27 | 10 | 18 | 3 | 4 | 1 | 7 | 2 | 5 | 3 | 8 | 20 | 14 | 14 | 1 | 4 | 1 | 7 | 3 | 5 | 20 | 13 | 12 | 0 | 4 | 1 | 7 | 3 | 5 |
| AFFECTS PROFESSIONAL PROGRESSION (e.g., lengthens training, less time to prepare for exams, time for professional readjustment upon return, career suicide, loss of partnerships) | 31 | 7 | 18 | 2 | 3 | 1 | 7 | 1 | 5 | 1 | 15 | 26 | 7 | 15 | 1 | 3 | 1 | 7 | 2 | 5 | 28 | 7 | 10 | 0 | 3 | 1 | 7 | 3 | 4 |
| NEGATIVE COLLEAGUE PERCEPTIONS (e.g., colleagues think itâ€™s a holiday, colleagues have to cover) | 25 | 8 | 24 | 1 | 4 | 1 | 7 | 2 | 6 | 4 | 7 | 18 | 9 | 21 | 1 | 4 | 2 | 7 | 3 | 5 | 14 | 12 | 18 | 1 | 4 | 2 | 7 | 3 | 5 |
| USE OF TIME (e.g., using annual leave to spend time on international placements, physically spending time on placements that could be spent in another way) | 21 | 17 | 17 | 3 | 4 | 1 | 7 | 2 | 5 | 2 | 6 | 12 | 21 | 15 | 1 | 4 | 1 | 7 | 3.25 | 5 | 11 | 19 | 13 | 2 | 4 | 1 | 6 | 3 | 5 |
| LOSS OF INTEREST IN PROFESSION (e.g., not wanting to work in your profession when home) | 36 | 9 | 13 | 0 | 3 | 1 | 7 | 2 | 4 | 3 | 13 | 32 | 9 | 8 | 0 | 3 | 1 | 6 | 2 | 4 | 31 | 7 | 6 | 1 | 3 | 1 | 6 | 2 | 4 |
| PROFESSIONAL REVALIDATION ISSUES (e.g., gaps in consultants portfolio) | 28 | 8 | 18 | 4 | 3 | 1 | 7 | 2 | 5 | 2 | 10 | 20 | 11 | 17 | 1 | 4 | 1 | 7 | 3 | 5 | 11 | 15 | 18 | 1 | 4 | 1 | 7 | 3.25 | 5 |
| LITIGATION (e.g., legal issues involving clinical/professional risk) | 29 | 12 | 12 | 5 | 3 | 1 | 7 | 2 | 4 | 3 | 10 | 25 | 13 | 8 | 3 | 3 | 1 | 7 | 2 | 4 | 27 | 6 | 10 | 2 | 3 | 1 | 7 | 3 | 4 |
| SECURITY (e.g., exposure to aggression, violence and death, becoming a victim of crime, political unrest) | 21 | 11 | 26 | 0 | 4 | 1 | 7 | 3 | 5 | 4 | 6 | 15 | 13 | 21 | 0 | 4 | 1 | 7 | 3 | 5 | 6 | 10 | 29 | 0 | 5 | 2 | 7 | 4 | 5 |
| CARBON FOOTPRINT | 26 | 18 | 12 | 2 | 4 | 1 | 6 | 2 | 4 | 4 | 6 | 16 | 21 | 10 | 2 |  | 1 | 77 | 3 | 4 | 15 | 19 | 9 | 2 | 4 | 1 | 7 | 3 | 4 |
| CULTURE SHOCK | 20 | 13 | 24 | 1 | 4 | 1 | 7 | 3 | 5 | 2 | 7 | 16 | 17 | 16 | 0 | 4 | 1 | 7 | 3 | 5 | 15 | 15 | 15 | 0 | 4 | 1 | 6 | 3 | 5 |
| ENVIRONMENTAL AND INFRASTRUCTURAL RISK (e.g., being in dangerous infrastructures and environments) | 19 | 9 | 29 | 1 | 5 | 1 | 7 | 3 | 5 | 3 | 5 | 8 | 8 | 33 | 0 | 5 | 1 | 6 | 4 | 5 | 8 | 6 | 31 | 0 | 5 | 2 | 7 | 4 | 5 |
| EXTREME NATIONALISM TOWARDS UK | 32 | 15 | 7 | 4 | 3 | 1 | 7 | 1 | 4 | 1 | 14 | 33 | 12 | 2 | 2 | 3 | 1 | 6 | 2 | 4 |  |  |  |  |  |  |  |  |  |
| EXPERIENCING NEGATIVE FEELINGS (e.g., feeling as though imposing on UK colleagues to provide cover, feeling failure, feeling out of depth, frustration, guilt and regret about death) | 26 | 12 | 19 | 1 | 4 | 1 | 7 | 2 | 5 | 1 | 8 | 18 | 14 | 16 | 1 | 4 | 1 | 6 | 3 | 5 | 13 | 16 | 13 | 3 | 4 | 1 | 7 | 3 | 5 |
| FINANCIAL LOSS (e.g., costs of getting involved, loss of earnings, pension or employment entitlement) | 19 | 8 | 31 | 0 | 5 | 1 | 7 | 3 | 6 | 3 | 2 | 6 | 11 | 31 | 1 | 5 | 2 | 7 | 4 | 5.8 | 7 | 4 | 34 | 0 | 5 | 2 | 7 | 4.5 | 5 |
| HEALTH CONSEQUENCES (e.g., animal bites, tropical diseases, STDâ€™s, injuries and transport accidents, infection, jet lag, skin disease) | 9 | 8 | 40 | 1 | 5 | 1 | 7 | 4 | 5 | 3 | 1 |  |  |  |  |  |  |  |  |  |  |  |  |  |  |  |  |  |  |
| PSYCHOLOGICAL CONSEQUENCES (e.g., depression, anxiety, stress, traumatisation and nervousness) | 14 | 13 | 29 | 2 | 5 | 1 | 7 | 3.25 | 5 | 1 | 2 | 12 | 11 | 23 | 3 | 4.5 | 1 | 6 | 3 | 5 | 8 | 8 | 26 | 3 | 5 | 1 | 6 | 4 | 5 |
| COMPROMISES OF HEALTH AND SAFETY | 21 | 15 | 21 | 1 | 4 | 1 | 7 | 3 | 5 | 1 | 6 | 12 | 15 | 22 | 0 | 4 | 2 | 7 | 3.5 | 5 | 12 | 17 | 15 | 1 | 4 | 1 | 7 | 3 | 5 |
| EXHAUSTION AND BURN OUT | 23 | 15 | 20 | 0 | 4 | 1 | 7 | 2 | 5 | 1 | 10 | 16 | 11 | 22 | 0 | 4 | 1 | 7 | 2 | 5 | 17 | 17 | 10 | 1 | 4 | 1 | 7 | 3 | 4 |
| LONELINESS (e.g., lone working, isolation, social isolation, no or few friends in host country) | 21 | 10 | 27 | 0 | 4 | 1 | 6 | 3 | 5 | 3 | 8 | 12 | 16 | 21 | 0 | 4 | 1 | 7 | 3.5 | 5 | 11 | 17 | 17 | 0 | 4 | 2 | 7 | 3.5 | 5 |
| MISSING THINGS AT HOME (e.g., missing home comforts, missing life in the UK, time away from family and friends) | 18 | 11 | 29 | 0 | 4.5 | 1 | 7 | 3 | 5 | 7 | 3 | 11 | 15 | 23 | 0 | 4 | 1 | 6 | 4 | 5 | 14 | 11 | 20 | 0 | 4 | 1 | 7 | 3 | 5 |
| LOSS OF INTEREST IN GLOBAL HEALTH AND INTERNATIONAL PLACEMENTS (e.g., not wanting to do it again, negative perceptions) | 37 | 7 | 13 | 1 | 3 | 1 | 7 | 2 | 4 | 1 | 13 | 32 | 7 | 9 | 1 | 3 | 1 | 6 | 2 | 4 | 28 | 4 | 12 | 1 | 3 | 1 | 7 | 3 | 5 |
| SOCIO-CULTURAL RISK (e.g., corruption, local resistance to western influence) | 27 | 11 | 18 | 2 | 4 | 1 | 7 | 2 | 5 | 2 | 7 | 18 | 19 | 10 | 2 | 4 | 1 | 6 | 3 | 4 | 12 | 15 | 15 | 3 | 4 | 2 | 7 | 3 | 5 |
| BECOMING JUDGEMENTAL | 27 | 12 | 17 | 2 | 4 | 1 | 7 | 2 | 5 | 1 | 7 | 17 | 16 | 16 | 0 | 4 | 1 | 6 | 3 | 5 | 19 | 19 | 7 | 0 | 4 | 2 | 6 | 3 | 4 |
| NEGATIVE FEELINGS TOWARDS THE NHS (e.g., questioning NHS, questioning the disposable culture of NHS, having a different system to compare to NHS) | 31 | 7 | 20 | 0 | 3 | 1 | 7 | 2 | 5 | 1 | 10 | 28 | 8 | 13 | 0 | 3 | 1 | 6 | 2 | 5 | 27 | 8 | 10 | 0 | 3 | 1 | 6 | 2 | 4 |

**8: Table to show levels of consensus in Delphi study and the final set of core outcomes**

| Core Outcome | | Met consensus at round | | Percentage consensus | | Positive or negative | | Rank | |
| --- | --- | --- | --- | --- | --- | --- | --- | --- | --- |
| INCREASED AWARENESS OF/KNOWLEDGE ABOUT CULTURAL DIFFERENCES AND SIMILARITIES (e.g., understanding key issues within a culture, culturally acceptable behaviour and cultures of UK immigrants, learning about, accepting and changing assumptions about other cultures) | | 2 | | 100 | | + | | 1 | |
| INCREASED AWARENESS OF/KNOWLEDGE ABOUT THE CULTURAL ASPECTS OF HEALTH (e.g., greater understanding of health promotion, how culture affects daily life and professional work, cultural differences in health, the effects of politics on health, sustainable healthcare) | | 2 | | 100 | | + | | 1 | |
| ABILITY TO WORK WITH LIMITED RESOURCES (e.g., being more resourceful, ability to target resources, ability to find solutions despite limited resources, making use of everything available, ability to work without reliance on technology, manage in a low resource setting) | | 2 | | 95 | | + | | 3 | |
| INCREASED AWARENESS OF/KNOWLEDGE ABOUT CULTURE IN PRACTICAL ASSESSMENTS (e.g., the importance of collecting relevant cultural information about peopleâ€™s presenting health problems and learning how to conduct cultural assessments and culturally based physical assessments) | | 2 | | 93 | | + | | 4 | |
| ABILITY TO APPLY CLINICAL SKILLS TO ANOTHER CONTEXT (e.g., a more challenging environment or a low resource setting) | | 2 | | 93 | | + | | 4 | |
| ABILITY TO BE ADAPTABLE AND INNOVATIVE IN TEACHING (e.g., ability to transfer skills and knowledge to the most influential people or to another context, recognising different learning styles, being adaptable in assessment) | | 2 | | 93 | | + | | 4 | |
| INCREASED AWARENESS OF/KNOWLEDGE ABOUT HOW OTHER HEALTHCARE SYSTEMS FUNCTION (e.g., developed insight into disparities within healthcare systems, understanding of other systems) | | 2 | | 93 | | + | | 4 | |
| ABILITY TO COPE (e.g., improved coping strategies, ability to deal with lack of structure, knock backs and stress, being unfazed by things and taking things in stride, new approach to guilt for patients problems) | | 2 | | 93 | | + | | 4 | |
| INCREASED CULTURAL SENSITIVITY (e.g., sensitivity to reasoning behind cultural differences, feelings of minority and language barriers) | | 2 | | 91 | | + | | 9 | |
| UNDERSTANDING THAT WORDS AND BEHAVIOURS CAN HAVE DIFFERENT MEANINGS (e.g., understanding how words are perceived by others, understanding how to speak and behave so as not offend people) | | 2 | | 91 | | + | | 9 | |
| ABILITY TO APPLY KNOWLEDGE ACROSS SYSTEMS (e.g., ability to apply knowledge from host system to UK and vice versa, using knowledge gained in system to improve/change another) | | 2 | | 91 | | + | | 9 | |
| DEVELOPMENT OF A NEW PERSPECTIVE (e.g., revising assumptions, seeing things differently, changed world views and outlook, look at everything in a new light, openness to new experiences, put things into perspective) | | 2 | | 91 | | + | | 9 | |
| IMPROVED FLEXIBILITY AND ADAPTABILITY (e.g., acceptance of other ways of working, adaptation to responsibility, being able to adapt more easily to unfamiliar situations, able to cope more easily with change, gaining a wider perspective, understanding the flexibility of roles) | | 2 | | 91 | | + | | 9 | |
| ABILITY TO BE INNOVATE WHEN OVERCOMING CHALLENGES (i.e., finding unique ways of overcoming cultural and language challenges) | | 2 | | 91 | | + | | 9 | |
| INCREASED RESPECT FOR OTHER CULTURES | | 2 | | 90 | | + | | 15 | |
| INCREASED UNDERSTANDING OF BASIC SKILLS AND IDEAS (i.e., back to basics, e.g., basic observations using eyes, less reliance on lab tests and technology, basic clinical skills and science) | | 2 | | 90 | | + | | 15 | |
| CONFIDENCE IN TEACHING ABILITY (e.g., being more comfortable around others, confidence public speaking, confidence in transferring knowledge) | | 2 | | 90 | | + | | 15 | |
| IMPROVED CONFIDENCE (e.g., in caring for clients from another culture, in quality improvement methods, to take bolder steps, to address challenging situations, self-confidence, confidence in professional ability,) | | 2 | | 90 | | + | | 15 | |
| CONFIDENCE TO WORK IN OTHER LOCATIONS (e.g., confidence to move to another city/country, working with UK multicultural/ underserved populations) | | 2 | | 89 | | + | | 19 | |
| INCREASED AWARENESS OF/KNOWLEDGE ABOUT GLOBAL ISSUES (e.g., re-evaluating world issues, shared purpose) | | 2 | | 88 | | + | | 20 | |
| INCREASED AWARENESS OF/KNOWLEDGE ABOUT CONDITIONS AND PROCEDURES RARELY ENCOUNTERED IN THE UK (e.g., greater understanding of procedures not used in the UK, unfamiliar equipment and delayed presentations, better management of conditions that are not common in the UK) | | 2 | | 88 | | + | | 20 | |
| INCREASED AWARENESS OF/KNOWLEDGE ABOUT TROPICAL DISEASES | | 2 | | 88 | | + | | 20 | |
| INCREASED AWARENESS OF/KNOWLEDGE ABOUT THE IMPORTANCE OF MUTUAL LEARNING AND RESPECT (i.e., greater understanding of reciprocal learning) | | 2 | | 88 | | + | | 20 | |
| ABILITY TO BE ADAPTABLE IN LEADING (e.g., able to lead in complex novel situations, ability to compromise not dictate) | | 2 | | 88 | | + | | 20 | |
| ABILITY TO WORK WITHIN A SYSTEM WITH UNFAMILIAR POWER DYNAMICS | | 2 | | 88 | | + | | 20 | |
| ABILITY TO ADAPT SOCIAL NORMS TO MEET NEEDS OF ANOTHER CULTURE (e.g., change behaviours to fit into another culture, being aware of own social norms and adapting them) | | 2 | | 88 | | + | | 20 | |
| ABILITY TO EXCHANGE IDEAS WITH THOSE FROM ANOTHER CULTURE | | 2 | | 88 | | + | | 20 | |
| INCREASED SELF-AWARENESS (e.g., understanding own skills and limitations, how to challenge own beliefs and importance of reflecting on own situation) | | 2 | | 88 | | + | | 20 | |
| PATIENCE AND TOLERANCE (e.g., accepting and working at other peoples pace, more tolerant) | | 2 | | 88 | | + | | 20 | |
| PROACTIVITY (e.g., thinking on feet, using initiative, efficiency, get on with things rather than look for someone to blame) | | 2 | | 88 | | + | | 20 | |
| ABILITY TO WORK WITH RESOURCES AVAILABLE IN SPECIFIC CONTEXTS (i.e., understanding the reasons behind lack of resources) | | 2 | | 88 | | + | | 20 | |
| ABILITY TO WORK TOWARDS SOLUTIONS (e.g., solution focused approach) | | 2 | | 88 | | + | | 20 | |
| UNDERSTANDING THAT SPEED AND LANGUAGE COMPETENCY AFFECT COMMUNICATION (e.g., awareness of how speed affects comprehension, understanding language differences and checking recipient comprehension, ability to use an interpreter) | | 2 | | 86 | | + | | 33 | |
| INCREASED AWARENESS OF/KNOWLEDGE ABOUT THE IMPORTANCE OF COMMUNITY PARTICIPATION IN HEALTH (e.g., understanding the community and social influences on health, the role of the community in health, public health and the importance of community work) | | 2 | | 86 | | + | | 33 | |
| ABILITY TO USE A BROADER RANGE OF CLINICAL SKILLS (e.g., enhancing existing skills and acquiring new clinical skills, greater all round competence) | | 2 | | 86 | | + | | 33 | |
| UNDERSTANDING THAT CHANGING BEHAVIOUR IS COMPLEX (e.g., understanding how to make small changes and not to force your perspective onto others,) | | 2 | | 86 | | + | | 33 | |
| ABILITY TO IMPROVE SERVICE (e.g., renewed enthusiasm for service improvement) | | 2 | | 86 | | + | | 33 | |
| INCREASED STAFF KNOWLEDGE AND SKILLS (e.g., increased staff knowledge of low cost healthcare, more knowledgeable staff able to cover more areas, to discover better ways of doing things and more aware of waste reduction) | | 2 | | 86 | | + | | 33 | |
| INCREASED AWARENESS OF/KNOWLEDGE ABOUT HOW CONTEXT AFFECTS COMMUNICATION (e.g., effectively conveying ideas in a contextually appropriate way) | | 2 | | 84 | | + | | 39 | |
| INCREASED AWARENESS OF/KNOWLEDGE ABOUT THE NEED FOR AND IMPORTANCE OF TRAINING (i.e., understanding how important effective training is in) | | 2 | | 84 | | + | | 39 | |
| IMPROVEMENT IN TEACHING SKILLS (e.g., learning new techniques, greater training delivery skills, lecturing skills and small group teaching skills) | | 2 | | 84 | | + | | 39 | |
| ABILITY TO DEAL WITH THE UNEXPECTED | | 2 | | 84 | | + | | 39 | |
| ABILITY TO MANAGE PROJECTS | | 3 | | 84 | | + | | 99 | |
| DEEPER ENGAGEMENT WITH ISSUES OF EQUALITY AND DIVERSITY | | 2 | | 83 | | + | | 43 | |
| ABILITY TO OVERCOME COMMUNICATION CHALLENGES (e.g., ability to communicate effectively in high pressure situations, engage in challenging conversations and liaise between groups) | | 2 | | 83 | | + | | 43 | |
| ABILITY TO BE INNOVATIVE WITH CLINICAL SKILLS (e.g., use of innovative techniques, finding new ways to approach a condition, new ways of working) | | 2 | | 83 | | + | | 43 | |
| APPRECIATION OF HAVING THE RIGHT TOOLS AND EQUIPMENT TO BE ABLE TO DO THE JOB (i.e., resources: technical equipment, disposal equipment, cleaning products and protective equipment) | | 2 | | 83 | | + | | 43 | |
| APPRECIATION OF EXCELLENT HUMAN RESOURCE IN THE NHS (e.g., multidisciplinary teams, HR structures, appreciation of own profession, understanding hierarchy and the importance of each person within it) | | 2 | | 83 | | + | | 43 | |
| IMPROVED EMOTIONAL INTELLIGENCE (e.g., changed engagement with self, knowledge and world) | | 2 | | 83 | | + | | 43 | |
| ABILITY TO IDENTIFY AND ANTICIPATE POTENTIAL PROBLEMS (e.g., identify problems when setting up a new project) | | 2 | | 83 | | + | | 43 | |
| INCREASED AWARENESS OF/KNOWLEDGE ABOUT APPROPRIATE CLINICAL BEHAVIOUR (e.g., knowing when to stop and when to move forward, when to ask for help and different populations needs) | | 2 | | 82 | | + | | 50 | |
| ABILITY TO MAKE INDEPENDENT CLINICAL DECISIONS (e.g., ability to make an urgent decision in an emergency, dealing with uncertain outcomes, evaluating risks to patients and self) | | 2 | | 81 | | + | | 51 | |
| UNDERSTANDING OWN POTENTIAL TO EMPOWER PEOPLE | | 2 | | 81 | | + | | 51 | |
| ABILITY TO WORK AS PART OF A TEAM (e.g., understanding team group norms, perception of roles within the group, managing personal objectives within a group) | | 2 | | 81 | | + | | 51 | |
| ABILITY TO BUILD A GLOBAL NETWORK | | 2 | | 81 | | + | | 51 | |
| ABILITY TO DISSEMINATE BEST PRACTICE GLOBALLY | | 2 | | 81 | | + | | 51 | |
| APPRECIATION OF FREE UNIVERSAL HEALTH (e.g., the NHS system of free healthcare for all, privilege and opportunity, the expectations that are placed on NHS by service users) | | 2 | | 81 | | + | | 51 | |
| IMPROVED SITUATIONAL AWARENESS (i.e., understanding your environment so you can understand what to do) | | 2 | | 81 | | + | | 51 | |
| INCREASED JOB SATISFACTION (e.g., increased motivation and morale within profession, renewed passion for work, sense of reward) | | 2 | | 81 | | + | | 51 | |
| PERSONAL SATISFACTION (e.g., personal achievements and challenges, new experiences, experiencing a different lifestyle, a holiday, appreciation of own life, personal fulfilment) | | 2 | | 81 | | + | | 51 | |
| CAN-DO ATTITUDE | | 3 | | 81 | | + | | 100 | |
| ABILITY TO CO-OPERATE (e.g., willingness to see another point of view) | | 2 | | 79 | | + | | 60 | |
| APPRECIATION OF CLINICAL GOVERNANCE PROCEDURES WITHIN NHS (e.g., waste disposal, audit, teamwork, education system, tests and investigations) | | 2 | | 79 | | + | | 60 | |
| APPRECIATION OF THE IMPORTANCE OF CARE AND COMPASSION (e.g., ability to compare compassion in both systems, empathy and fairness) | | 2 | | 79 | | + | | 60 | |
| ABILITY TO PROVIDE BETTER CARE (e.g., ability to integrate primary and secondary care, to provide multicultural care, to develop most effective approaches to care and taking responsibility for providing quality of care) | | 2 | | 79 | | + | | 60 | |
| INCREASED AWARENESS OF/KNOWLEDGE ABOUT THE POSITIVE IMPACT OF CLINICAL POLICIES AND GOVERNANCE (e.g., understanding the benefits of a comprehensive checklist) | | 3 | | 78 | | + | | 101 | |
| INCREASED AWARENESS OF/KNOWLEDGE ABOUT ETHICS (i.e., experiencing ethical dilemmas, understanding the importance of ethics) | | 2 | | 78 | | + | | 64 | |
| CHANGED PERCEPTION OF OTHERNESS (e.g., understanding importance of being a friendly stranger in UK, feeling like a foreigner) | | 2 | | 78 | | + | | 64 | |
| INTEGRITY | | 2 | | 78 | | + | | 64 | |
| INDEPENDENCE (e.g., lone working) | | 2 | | 78 | | + | | 64 | |
| ABILITY TO PLAN AND ORGANISE (e.g., ability to set direction, improved audit skills) | | 2 | | 78 | | + | | 64 | |
| ABILITY TO MAKE DECISIONS (e.g., understanding who the decision is for, taking action on decision, making judgements | | 2 | | 78 | | + | | 64 | |
| ABILITY TO MANAGE RISK (e.g., manage risk in advance, evaluation of environment, understanding the clinical importance of risk management and the wider implication of poorly managed risk) | | 2 | | 78 | | + | | 64 | |
| INCREASED PATIENT SATISFACTION (e.g., staff better able to respond to UK multicultural populations, staff able to compare how systems affect patient satisfaction, have greater relationships with multicultural population, more in tune with patients and more aware of individual needs of patients). | | 2 | | 77 | | + | | 71 | |
| ABILITY TO COMMUNICATE NON-VERBALLY | | 2 | | 76 | | + | | 72 | |
| ABILITY TO ESTABLISH COMMUNICATION SYSTEMS (e.g., formal and informal) | | 3 | | 76 | | + | | 102 | |
| INCREASED CLINICAL KNOWLEDGE IN RELATION TO OTHER PROFESSIONS (e.g., doctors understanding nurses and vice versa, multi-disciplinary awareness) | | 3 | | 76 | | + | | 102 | |
| ABILITY TO GET THE MOST OUT OF PEOPLE (e.g., encouraging people to work together, recognise their own strengths and to take possession of their own work/projects, ability to assess the capability of others) | | 2 | | 76 | | + | | 72 | |
| ABILITY TO MANAGE PEOPLE (e.g., able to allocate tasks and co-ordinate people, to deal with people with differing objectives, to negotiate with multiple stakeholders, to manage difficult people) | | 2 | | 76 | | + | | 72 | |
| ABILITY TO DEVELOP FRIENDSHIPS (e.g., relationship formation skills, developing new friendships) | | 2 | | 76 | | + | | 72 | |
| ABILITY TO MANAGE SELF (e.g., own expectations, self-reliance, self-management, self-assurance, reflexivity) | | 2 | | 76 | | + | | 72 | |
| CHANGED JUDGEMENT (e.g., non-judgemental attitude, changed self-judgement) | | 2 | | 76 | | + | | 72 | |
| DIPLOMACY | | 2 | | 76 | | + | | 72 | |
| ABILITY TO FIND FACTS TO SOLVE PROBLEMS | | 2 | | 76 | | + | | 72 | |
| DEVELOPING REDUNDANT OR BAD SKILLS/ATTITUDES (e.g., developing non-transferable skills, bad habits, deskilling, returning with overconfidence in own ability, poorer communication skills, loss of confidence) | | 3 | | 76 | | - | | 102 | |
| FINANCIAL LOSS (e.g., costs of getting involved, loss of earnings, pension or employment entitlement) | | 4 | | 76 | | + | | 112 | |
| REDUCTION IN NHS DROP OUTS (e.g., increased staff retention, when they volunteer and come back to NHS) | | 3 | | 75 | | + | | 105 | |
| ABILITY TO OBSERVE AND EXAMINE PATIENTS (e.g., increased intuitive knowledge of clinical signs and clinical judgement ability to make diagnosis without investigations) | | 2 | | 74 | | + | | 80 | |
| ABILITY TO WORK IN A PROFESSIONALLY COMPETENT WAY (e.g., having wider view of profession, intellectual development, reminder of professional responsibilities, stronger work ethic) | | 2 | | 74 | | + | | 80 | |
| INCREASED UNDERSTANDING OF HOW TO BE A GOOD TEACHER (e.g., allowing students to learn from mistakes, ability to suggest and acknowledge improvements in teaching, understanding how communication affects learning, how to target training most effectively and the importance of experiential learning) | | 2 | | 74 | | + | | 80 | |
| ACT AS A ROLE MODEL (e.g., lead by example) | | 2 | | 74 | | + | | 80 | |
| INFLUENCES CAREER PATHWAY (i.e., affects specialism choice, exploration of potential career pathways, pursuing careers in primary care, family practice, public service, sub-specialism in global health, teaching) | | 2 | | 74 | | + | | 80 | |
| ABILITY TO MANAGE TIME AND PRIORITISE (e.g., ability to respond quickly in an emergency, managing immediate need vs long term need, prioritisation of limited resources) | | 2 | | 74 | | + | | 80 | |
| INCREASED ABILITY TO CHANGE BEHAVIOUR IN COLLEAGUES OR PATIENTS (e.g., ability to implement behaviour change and to assess the impact of healthcare systems) | | 4 | | 73 | | + | | 113 | |
| ABILITY TO MANAGE TRAGEDIES | | 3 | | 73 | | + | | 106 | |
| REDUCTION IN STAFF COMPETENCE (e.g., brain drain reversal: NHS loss of competent staff to overseas placements, staff unable to cope with paperwork on return) | | 4 | | 73 | | - | | 113 | |
| EXPOSURE TO ETHICAL DILEMMAS (e.g., expected to work outside of competency, to do clinical work, little regulation, little supervision, too much responsibility) | | 3 | | 73 | | + | | 106 | |
| NO RECOGNITION OR ACCREDITATION UPON RETURN | | 4 | | 73 | | + | | 113 | |
| INCREASED INTERNATIONAL REPUTATION (of UK) | | 3 | | 73 | | + | | 106 | |
| INCREASED INTERNATIONAL REPUTATION OF NHS (e.g., greater fulfilment of social responsibility) | | 2 | | 73 | | + | | 86 | |
| ABILITY TO VERBALISE KNOWLEDGE (e.g., ability to verbalise core concepts and deep knowledge, ability to explain complex ideas to others) | | 2 | | 72 | | + | | 87 | |
| INCREASED AWARENESS OF/KNOWLEDGE ABOUT THE IMPORTANCE OF TRUST BETWEEN COLLEAGUES WITHIN HEALTHCARE SYSTEMS | | 2 | | 72 | | + | | 87 | |
| INCREASED AWARENESS OF AND KNOWLEDGE THE FUNCTIONING OF SYSTEMS (e.g., able to identify stakeholders and change agents, understanding influencing patterns of those in power, value systems and the difficulty of questioning organisations) | | 2 | | 72 | | + | | 87 | |
| REFRESHMENT AND REINVIGORATION (e.g., chance to take time away to become refreshed and feel reinvigorated to work upon return) | | 2 | | 72 | | + | | 87 | |
| INCREASED AWARENESS OF/KNOWLEDGE ABOUT THE IMPORTANCE OF CONSCIOUSLY MAKING AN EFFORT TO GET ON WITH COLLEAGUES (e.g., learning colleagueâ€™s names) | | 3 | | 71 | | + | | 109 | |
| ABILITY TO MANAGE HEALTHCARE ENVIRONMENTS (e.g., ability to manage wards and staff) | | 2 | | 71 | | + | | 91 | |
| INCREASED AWARENESS OF/KNOWLEDGE ABOUT THE COSTS OF HEALTHCARE | | 2 | | 71 | | + | | 91 | |
| ABILITY TO ACCEPT AND UNDERSTAND FAILURE (e.g., to continue with something that did not have desired outcome at first, learning to accept failure, thinking differently about failure, persistence) | | 2 | | 71 | | + | | 91 | |
| HUMILITY (including professional humility) | | 2 | | 71 | | + | | 91 | |
| ABILITY TO THINK THROUGH PROBLEMS IN A LOGICAL WAY (e.g., analytical/lateral thinking) | | 2 | | 71 | | + | | 91 | |
| ABILITY TO ENGAGE SENIOR PEOPLE | | 2 | | 70 | | + | | 96 | |
| LOSS OF INTEREST IN PROFESSION (e.g., not wanting to work in your profession when home) | | 4 | | 70 | | - | | 114 | |
| EXTREME NATIONALISM TOWARDS UK | | 3 | | 70 | | - | | 110 | |
| HEALTH CONSEQUENCES (e.g., animal bites, tropical diseases, STDâ€™s, injuries and transport accidents, infection, jet lag, skin disease) | | 2 | | 70 | | + | | 96 | |
| INCREASED WORKFORCE PRODUCTIVITY | | 3 | | 70 | | + | | 110 | |
| NHS BECOMES A MORE ATTRACTIVE EMPLOYER (e.g., an employer that offers staff the opportunity to volunteer) | | 2 | | 70 | | + | | 96 | |
| REINFORCED ETHNIC AND CULTURAL IDENTITY (e.g., understanding of own ethic and cultural identity) | | No Consensus | | 0 | | + | |  | |
| ABILITY TO LISTEN | | No Consensus | | 0 | | + | |  | |
| INCREASED AWARENESS OF/KNOWLEDGE ABOUT THE IMPORTANCE OF ASSESSING HEALTHCARE ON AN INDIVIDUAL BASIS (i.e. the uniqueness of each patient) | | No Consensus | | 0 | | + | |  | |
| ABILITY TO APPLY EVIDENCE BASED PRACTICE (e.g., understanding its importance (sometimes through being unable to apply it overseas), understanding how to apply it innovatively with limited resources) | | No Consensus | | 0 | | + | |  | |
| ABILITY TO GIVE AND ACCEPT PRAISE | | No Consensus | | 0 | | + | |  | |
| ABILITY TO ENCOURAGE OTHERS TO TAKE RESPONSIBILITY FOR OWN HEALTH | | No Consensus | | 0 | | + | |  | |
| ABILITY TO SPEAK THE HOST LANGUAGE | | No Consensus | | 0 | | + | |  | |
| ABILITY TO CHALLENGE BREACHES OF PRIVACY AND CONFIDENTIALITY (e.g., ability to stand up for patients/peoples rights if they are jeopardised, increased awareness of human rights, ability to respect regulatory standards of home and overseas regulatory bodies) | | No Consensus | | 0 | | + | |  | |
| AN UPPER HAND WHEN COMPETING FOR CAREERS | | No Consensus | | 0 | | + | |  | |
| SPIRITUAL DEVELOPMENT | | No Consensus | | 0 | | + | |  | |
| ESCAPISM (e.g., freedom from bureaucracy, space outside of regular routine to clarify objectives, escape from agendas and workload, a chance to take time out of training and practice) | | No Consensus | | 0 | | + | |  | |
| IMPROVED RESEARCH SKILLS (e.g., grant application skills, research design and implementation) | | No Consensus | | 0 | | + | |  | |
| ABILITY TO PRESENT WORK | | No Consensus | | 0 | | + | |  | |
| ABILITY TO WRITE REPORTS AND ACADEMIC PIECES | | No Consensus | | 0 | | + | |  | |
| COSTS TO BRITISH PATIENTS (e.g., staff desensitised, staff less tolerant and patient, staff bringing tropical illnesses to UK) | | No Consensus | | 0 | | + | |  | |
| LOSS OF TRAINED STAFF (e.g., utilisation of key staff time, financial cost of losing staff, having to find cover for staff) | | No Consensus | | 0 | | + | |  | |
| NEGATIVE PERCEPTIONS OF NHS (e.g., NHS reputation jeopardised if a health link is badly organised) | | No Consensus | | 0 | | + | |  | |
| DISTRACTED STAFF (e.g., staff going on international placements coming back disengaged with UK work and pre-occupied) | | No Consensus | | 0 | | + | |  | |
| DIFFICULTY GETTING THE JOB OR TRAINING POSITION THAT YOU WANT UPON RETURN (e.g., returning to work in a locum position, not having a permanent job upon return) | | No Consensus | | 0 | | + | |  | |
| REDUCED EXPERIENCE AND EXPOSURE TO UK PROCEDURES, PROTOCOLS AND RESEARCH (e.g., NHS procedures that donâ€™t exist in host country, missing out on formal training and conferences, chronic disease management over time, health conditions that are common in UK and not in host country, NHS protocol and updates, loss of professional networks and relationships) | | No Consensus | | 0 | | + | |  | |
| AFFECTS PROFESSIONAL PROGRESSION (e.g., lengthens training, less time to prepare for exams, time for professional readjustment upon return, career suicide, loss of partnerships) | | No Consensus | | 0 | | + | |  | |
| NEGATIVE COLLEAGUE PERCEPTIONS (e.g., colleagues think itâ€™s a holiday, colleagues have to cover) | | No Consensus | | 0 | | + | |  | |
| USE OF TIME (e.g., using annual leave to spend time on international placements, physically spending time on placements that could be spent in another way) | | No Consensus | | 0 | | + | |  | |
| PROFESSIONAL REVALIDATION ISSUES (e.g., gaps in consultants portfolio) | | No Consensus | | 0 | | + | |  | |
| LITIGATION (e.g., legal issues involving clinical/professional risk) | | No Consensus | | 0 | | + | |  | |
| SECURITY (e.g., exposure to aggression, violence and death, becoming a victim of crime, political unrest) | | No Consensus | | 0 | | + | |  | |
| CARBON FOOTPRINT | | No Consensus | | 0 | | + | |  | |
| CULTURE SHOCK | | No Consensus | | 0 | | + | |  | |
| ENVIRONMENTAL AND INFRASTRUCTURAL RISK (e.g., being in dangerous infrastructures and environments) | | No Consensus | | 0 | | + | |  | |
| EXPERIENCING NEGATIVE FEELINGS (e.g., feeling as though imposing on UK colleagues to provide cover, feeling failure, feeling out of depth, frustration, guilt and regret about death) | | No Consensus | | 0 | | + | |  | |
| PSYCHOLOGICAL CONSEQUENCES (e.g., depression, anxiety, stress, traumatisation and nervousness) | | No Consensus | | 0 | | + | |  | |
| COMPROMISES OF HEALTH AND SAFETY | | No Consensus | | 0 | | + | |  | |
| EXHAUSTION AND BURN OUT | | No Consensus | | 0 | | + | |  | |
| LONELINESS (e.g., lone working, isolation, social isolation, no or few friends in host country) | | No Consensus | | 0 | | + | |  | |
| MISSING THINGS AT HOME (e.g., missing home comforts, missing life in the UK, time away from family and friends) | | No Consensus | | 0 | | + | |  | |
| LOSS OF INTEREST IN GLOBAL HEALTH AND INTERNATIONAL PLACEMENTS (e.g., not wanting to do it again, negative perceptions) | | No Consensus | | 0 | | + | |  | |
| SOCIO-CULTURAL RISK (e.g., corruption, local resistance to western influence) | | No Consensus | | 0 | | + | |  | |
| BECOMING JUDGEMENTAL | | No Consensus | | 0 | | + | |  | |
| NEGATIVE FEELINGS TOWARDS THE NHS (e.g., questioning NHS, questioning the disposable culture of NHS, having a different system to compare to NHS) | | No Consensus | | 0 | | + | |  | |
| MEDICAL SCHOOL MORE ATTRACTIVE TO STUDENTS (e.g., if allows students to go abroad) | | No Consensus | | 0 | | + | |  | |
